# Spatiotemporal Dynamics of Dengue Risk in Bangladesh: A GIS Based Approach

**DOI:** 10.1029/2025GH001707

**Published:** 2026-07-21

**Authors:** Nusrat Zahan Jarin, Abinash Bhattachan, Obaidur Rahman

**Affiliations:** ^1^ Department of Geography University of California Santa Barbara Santa Barbara CA USA; ^2^ Department of Geosciences Texas Tech University Lubbock TX USA; ^3^ Department of Geography Oklahoma State University Stillwater OK USA

**Keywords:** dengue risk, spatiotemporal analysis, dengue hotspots, Bangladesh public health

## Abstract

Dengue, commonly known as breakbone fever, has been prevalent in Bangladesh since 2000. Monsoon conditions and warmer temperatures create suitable breeding environment for the vectors *Aedes aegypti* and *Aedes albopictus*. Although dengue outbreaks peak during the post‐monsoon months (September and October), 2023 showed an unusual shift, with cases peaking as early as June. The case fatality rate (0.54%) in 2023 was the highest in the past two decades. This study offers an exhaustive examination of the spatiotemporal dynamics of dengue incidence clusters and composite risk across Bangladesh using daily dengue case data from 2019 to 2024. Spatial autocorrelation analysis (Local Moran's *I*) revealed that the central and south coastal districts, especially Dhaka, Manikganj, and Barisal were the major dengue hotspots during the peak outbreak years (2019, 2023, and 2024). Jaccard Similarity Index (JSI) analysis further indicated strong seasonal and interannual reorganization of dengue hotspots, especially during the post‐monsoon season, with increasing hotspot concentration in coastal districts after 2020. To assess hazard, vulnerability and risk, we applied a GIS‐based multi‐criteria decision‐making model (MCDM) framework and compared both weighted (AHP‐based) and unweighted approaches. The weighted AHP model was more accurate (AUC = 0.75) than the unweighted model (AUC = 0.55) in predicting dengue risk zones. A negative binomial mixed‐effects regression showed that lagged temperature, precipitation and population density increased dengue incidence, while higher normalized difference built‐up index was associated with lower incidence. These findings emphasize the need for integrated dengue control strategies targeting high‐risk areas and equal healthcare access.

## Plain Language Summary

Dengue fever, transmitted by *Aedes* mosquitoes, is a major public health concern in Bangladesh. This study investigated dengue outbreaks from 2019 to 2024 to identify seasonal changes and risk factors driving the spread of the disease. Findings showed a significant rise in dengue cases in recent years, with 2023 recording the highest number of infections and deaths, along with an unusually early peak beginning in June. Central Dhaka and south coastal districts, particularly Barisal, consistently reported high dengue incidence. Dengue activity increased during the monsoon and post‐monsoon periods, likely due to higher rainfall and warmer temperatures that favor mosquito breeding. Statistical analysis showed that higher temperature, rainfall in the previous month and population density were associated with increased dengue incidence, while higher built‐up land intensity was linked to lower incidence. Using geospatial analysis and hotspot analysis techniques, this study identified both persistent and shifting dengue hotspots across Bangladesh. Coastal regions emerged as hotpots after 2020 placing additional pressure on public health systems. The findings also revealed that climatic, social and environmental factors contribute differently to dengue risk, underscoring the importance of targeted prevention.

## Key Points

- Dengue risk peaked during monsoon and post‐monsoon seasons, especially in Dhaka and coastal districts
- Higher temperature and rainfall in the previous month and population density were strongly linked to increased dengue incidence
- Dengue hotspots increasingly shifted to coastal districts south of Dhaka after 2020

## 1 Introduction

Dengue fever is an arboviral disease caused by the dengue virus (DENV) that affects about half of the global population (Haider et al., 2025; Kalimuddin et al., 2025; Messina et al., 2019). The primary vector of dengue fever are *Aedes aegypti* mosquitoes, predominantly found in tropical urban and peri‐urban areas of South Asia, Africa and Latin America (S. A. Ali & Ahmad, 2018; Engelthaler et al., 1997; Kalimuddin et al., 2025; Tabachnick & Powell, 1979; Telle et al., 2016). *Aedes albopictus*—common in rural areas, are also dengue vectors—but to a lower extent (Henry & Mendonça, 2020; Telle et al., 2016). DENV consists of four related but antigenically distinct serotypes, namely DENV‐1, DENV‐2, DENV‐3, and DENV‐4 (Chen & Vasilakis, 2011) and are diverse based on different geographic distributions (Araújo et al., 2009; Chen & Vasilakis, 2011; Rico‐Hesse, 1990; Twiddy et al., 2002; Villabona‐Arenas & de Andrade Zanotto, 2011) driven by climate variability, urbanization, and increased human mobility (Nakase et al., 2024; Paz‐Bailey et al., 2024). In 2024, dengue transmission reached unprecedented levels globally, with approximately 1.4 million cases in multiple regions (WHO, 2025).

In the past several decades, the incidence of DENV has experienced an abrupt and significant increase in the Americas (Dickin & Schuster‐Wallace, 2014; Henry & Mendonça, 2020), South‐East Asia (Jeefoo et al., 2011; Swami & Parthasarathy, 2021; Tsheten et al., 2021), with Asia accounting for more than 70% of the global disease burden (WHO, 2023). For example, the number of cases increased from 505,430 in 2000 to 5.2 million in 2019 (WHO, 2023). The year 2023 reported a historic high of over 6.5 million dengue cases with more than 7,000 dengue related deaths (WHO, 2023). The actual number of the cases could be much larger than WHO's estimates as Bhatt et al. (2013) estimated 390 million infections annually, of which 96 million were symptomatic resulting in 22,000 fatalities. Although incidence of DENV is increasing and expected to increase which during and in its wake causes economic and health care disruption in tropical, less developed countries, it is still considered as one of the neglected tropical diseases (WHO, 2012). Some research suggests an explosive increase in the geographic distribution of all four DENV serotypes is due to rapid urbanization coupled with global climate change (Messina et al., 2019).

Of all the DENV global hotspots, Bangladesh experienced a rapid increase in dengue cases in the recent decades (Haider et al., 2023; Sharmin et al., 2015). According to the WHO, the 2023 outbreak had DENV‐2 as the primary circulating serotype although it was reported that DENV‐3 in 2019 had become the dominant serotype (WHO, 2023). This heterogeneity of serotype caused second infection and increased the severity of this disease (WHO, 2023) as some research stated that areas with circulation of multiple serotypes of DENV tend to have more severe dengue incidence (Guzman et al., 2010; Kayesh et al., 2023; Lorono‐Pino et al., 1999). Following the 2023 epidemic, M. S. Hossain et al. (2023) and S. Hossain et al. (2023) reviewed dengue's history and future hazards in Bangladesh and observed that dengue risk has increased, and daily cases and deaths were primarily clustered around Dhaka, consistent with other studies (e.g., M. Ali et al., 2003; Banu et al., 2012; Kayesh et al., 2023). Recent research indicated that over 90% of yearly dengue infections occurred in August and September (Sharmin et al., 2018) after the monsoon season, however, dengue incidence started to rise at the end of the post monsoon season (October–November) in 2022 (Haider et al., 2023). Recent studies (e.g., Hasan et al., 2025; Kamal et al., 2023; Roy et al., 2024) have primarily only focused on Dhaka, short outbreak periods, or limited spatial scale, with relatively few analyses of district level dengue dynamics across the entire country. Understanding when and where regional dengue peaks will change is crucial for early warning, equity‐based distribution of resources and management purposes of the country. Even though Hossan et al. (2023) conducted an analysis to detect dengue incidence hotspots from January to October 2023 and found that Dhaka had the most clusters, their study did not investigate the seasonal and yearly shift of dengue hotspots and cold spots. Overall, limited attention has been given to seasonal and interannual variation in dengue hotspot distribution over a period of multiple years. In fact, studies that assess the risk zones along with climatic hazard and socially vulnerable zones for potential dengue incidence in district level scale in Bangladesh are still missing which does not allow for proper identification of priority zones for infectious disease mitigation planning. Hazard literature provides critical epidemiological evidence on the relationship between temperature, precipitation, elevation, and built environment, and their influence on mosquito breeding dynamics, vector survival, and viral replication. Previous studies by Harsha et al. (2023) and Tsheten et al. (2021) utilized hazard and vulnerability elements to define dengue risk in India and Bhutan respectively. This study aims to fill the existing gaps by answering the following research questions,

1. Were there any significant seasonal or annual shifts in dengue outbreak in Bangladesh over the last 6 years (2019–2024)?
2. How do seasonal clusters of dengue cases compare to seasonal dengue risks?
3. Which districts in Bangladesh are at high risk and are most vulnerable to dengue occurrence?

A better understanding of the seasonality and hazard‐vulnerability perspective of dengue risk as a public health disaster is needed for future preparedness. In our study, we utilized hazard literature to define how dengue transmission responds to climate, environment and built infrastructure. In data scarce regions such as Bangladesh, where long‐term high‐resolution dengue incidence data are limited and often affected by reporting inconsistencies, hazard literature offers a scientifically robust basis for identifying relevant indicators and structuring risk assessment. To this end, our study considers the climatic, environmental, and anthropogenic factors to determine areas of dengue risk with a special focus on seasonality. Recent studies indicate that the dengue peak season in Bangladesh is shifting and becoming more variable, with evidence of a shift from August to September during recent outbreaks, alongside interannual changes in peak timing and a gradual increase of the transmission season (M. Hossain et al., 2025; S. Hossain et al., 2025; Paul et al., 2021; Subarna & Al Saiyan, 2024). Based on these recent studies, this paper hypothesizes that there is a shift in seasonality in dengue incidence hotspots and risk zones throughout the 64 districts of Bangladesh. We investigate the relative contribution of social, climatic, and environmental factors in assessing overall dengue hazard, vulnerability, and risk. We created spatial and temporal distribution of dengue case clusters and composite risk in the 64 districts of Bangladesh from 2019 to 2024. In doing so, outcomes from our study will allow for proper representation of dengue risk zones coupled with actual dengue clusters aimed at serving as a guide for zonal prioritization, healthcare resource allocation and future epidemiological surveillance of dengue in Bangladesh.

## 2 Methods

### 2.1 Study Area

Bangladesh (Figure S1 in Supporting Information S1) is home to around 160 million people (2021 estimate), making it one of the most densely inhabited nations (BBS, 2022). The earliest documented cases of dengue occurred in the 1960s in Bangladesh, which was then known as East Pakistan, and the disease was commonly referred to as “Dacca fever” (WHO, 2023). Since 2010, dengue outbreaks have intensified during the rainy season, May–September (Y. H. Lai, 2018; Malik et al., 2017), coinciding with the months with warm temperature (Y. H. Lai, 2018; Ramachandran et al., 2016; Wangdi et al., 2018). Heavy rainfall, waterlogging, flooding, rising temperatures, and the abnormal changes in seasonality create favorable environment for dengue vectors (Salam et al., 2018; WHO, 2023). The predominantly low terrain and heavy rainfall during the monsoon season in the coastal areas of Bangladesh are prone to recurring floods and stagnant water almost every year (Alam & Ahamed, 2022; Diman & Tahir, 2012). Rapid urbanization, population growth, and inadequate drainage in unplanned urban areas further increase dengue risk by serving as mosquito breeding sites (Mehmood et al., 2021; Messina et al., 2019; Tsheten et al., 2021). District level dengue incidence data from 2019 to 2024 were analyzed and 2023 was identified as the peak year to generate dengue risk surface using corresponding environmental factors. It allowed us to compare the spatial variation of dengue case occurrences per 10,000 population and the dengue risk surface during the highest dengue occurrence year of 2023.

### 2.2 Data and Dengue Risk Components

Nine environmental data sets (Table S1 in Supporting Information S1) were considered to assess dengue hazard, vulnerability, and risk at seasonal and annual levels. All data sets were converted into raster format and standardized using the maxima‐minima method, suggested by previous studies (Gbetibouo et al., 2010; Patnaik & Narayanan, 2010; Ravindranath et al., 2011; Wiréhn et al., 2015). The maxima‐minima method was employed to maintain a common spatial extent because each data set had a different spatial extent. The raster layers were further standardized and reclassified from 0 to 1 scale to associate with low to high categories according to their functional relationship with dengue hazard, vulnerability and risk.

#### 2.2.1 Temperature

Temperature regulates mosquito population since higher temperatures accelerate the extrinsic incubation period of DENV in *Aedes* mosquitos and promote viral transmission (Ramachandran et al., 2016; Tsheten et al., 2021; Wangdi et al., 2018). Furthermore, warmer temperatures (around 35°C) speed up the blood meal's digestion, which improves mosquitoes' eating habits (Scott & Morrison, 2010; Tahir et al., 2023). The monthly average of daily maximum temperature data (from December 2022 to November 2023) for 95 meteorological stations in Bangladesh were collected from BMD (https://dataportal.bmd.gov.bd). BMD temperature data have been previously used in several dengue related studies in Bangladesh (e.g., Alam et al., 2025; M. Hossain et al., 2025; M. S. Hossain et al., 2023; S. Hossain et al., 2023, 2025). The BMD data set was bias corrected, and quality controlled during the internal data processing procedures. BMD provided a preprocessed data set in which missing values had already been addressed through their internal quality control procedures. To assess the seasonal temperature dynamics, monthly data were aggregated to derive seasonal average maximum temperature (°C) for winter (December, January, February), pre‐monsoon (March, April, May), monsoon (June, July, August) and post‐monsoon (September, October, November) seasons in 2023. The geocoded monthly temperature points were interpolated at 30‐m spatial resolution using IDW (inverse distance weighting) method in the spatial analyst toolset in ArcGIS pro (Version 3.1.0, Redwood, CA) and monthly rasters were aggregated into seasonal temperature layers (Figure S2 in Supporting Information S1).

#### 2.2.2 Precipitation

Total monthly rainfall can be indicative of increased possibility of stagnant water. The functional relationship of dengue risk with precipitation is assumed to be positive and linear in this study which is supported by previous studies (Chadee et al., 2005; Cheong et al., 2013; Das et al., 2014; Goh et al., 1987; Gould et al., 1970; Tsheten et al., 2021) which showed that increased precipitation was the prime facilitator of *Aedes* vector population. Other studies suggested that this relationship may be nonlinear (Sugeno et al., 2023), lagged (Lowe et al., 2018) and threshold based (Hashizume et al., 2012) under certain environmental conditions. Some even suggest that heavy rainfall can reduce mosquito survival (Sugeno et al., 2023; Zheng et al., 2024) and dengue risk becomes the highest within specific rainfall range (90–360 mm) (Hashizume et al., 2012). A linear positive relationship is adopted here as a first order approximation to capture the overall positive influence of rainfall on dengue risk. The bias corrected total monthly precipitation data (from December 2022 to November 2023) was collected from BMD for all stations. The data set was complete and missing values were addressed through BMD's internal quality control procedures. The precipitation raster was also interpolated from the geocoded station point data set using IDW and then aggregated into seasonal (winter, pre‐monsoon, monsoon, and post‐monsoon) precipitation using a raster calculator (Figures S3–S5 in Supporting Information S1).

#### 2.2.3 Population Density

High population density can increase the spread of dengue fever more quickly due to proximity of potential human hosts (Gubler, 1998; Hsueh et al., 2012). High population density can also result in more artificial water containers, greater human‐mosquito interaction, and increased population movement, which together can elevate the risk and impact of a dengue outbreak. Population density data, originally available at the district level, were collected from BBS (2022) census and were converted into raster format by assigning district level values to all pixels within each administrative boundary (Figure S6a in Supporting Information S1). The rasterization was performed using a consistent spatial resolution and extent aligned with other predictor variables to ensure the compatibility of the model. Population density has a positive linear relationship with dengue risk (Tsheten et al., 2021; Yue et al., 2018).

#### 2.2.4 Distance to Hospital

Proximity to a hospital or a healthcare facility was considered as a key factor in assessing the vulnerability to dengue outbreaks. M. Ali et al. (2003) found that dengue clusters were less prominent in areas far from major hospitals in Dhaka, suggesting proximity to hospitals as a major indicator in dengue diagnosis. Populations that live far from hospitals might face reduced access to prompt medical treatment and delayed diagnosis, increasing the potential loss and differential incapability to adapt with the outbreaks which are two major indicators to define vulnerability, according to Cutter (1996) and Dow (1992). While greater distance from healthcare facilities may increase vulnerability due to delayed treatment and limited access to care, it may also lead to underreporting of dengue cases, introducing potential detection bias. A list of hospitals was obtained from the facility registry of the Directorate General of Health Services (DGHS) (https://hrm.dghs.gov.bd/public/facility‐registry) and the geographic coordinates of each hospital was collected using Google Earth Pro. Coordinates were converted into point features, and reprojected to the WGS 1984 Web Mercator coordinate system to ensure consistency across all spatial layers using ArcGIS Pro. Using the nationwide hospital point data (*n* = 1,828) from DGHS, a rasterized layer was prepared to assess the Euclidean (shortest linear) distance (Esri, 2024) to the hospital (Figure S6b in Supporting Information S1). Greater distance was considered to have lower adaptive capacity and higher dengue vulnerability.

#### 2.2.5 Distance to Major Roads

Major roads and highways often are associated with urban landscapes (Tsheten et al., 2021) and greater human mobility (Qi et al., 2015) both of which are great facilitators of dengue vulnerability. The dengue incidence cluster declined after 1 km (Hsueh et al., 2012) and concentrated within 500 m (Li et al., 2018) of road network. The road network data set was obtained from the Bangladesh Roads and Highways Department (RHD, 2022) (Figure S7 in Supporting Information S1) (https://gis.rhd.gov.bd/portal/home/item.html?id=e7e196a285bf4d789bb9d7dcbca7b0c1). Major cities are situated adjacent to important transit hubs, increasing human accessibility and thus increasing the possibility of mosquito‐human interaction. The road network data set was reprojected to the WGS 1984 Web Mercator coordinate. The Euclidean distance to major roads (m) was calculated and reclassified from 0 to 1 in Figure S6c in Supporting Information S1 where values closer to 0 represent areas nearer to major roads and thus higher dengue vulnerability, while values closer to 1 represent areas farther from major roads and lower vulnerability.

#### 2.2.6 Normalized Difference Built‐Up Index (NDBI)

NDBI identifies built up areas (Zha et al., 2003) using reflectance values from the short‐wave infrared 1 (1.57–1.65 μm) and near infrared (0.85–0.88 μm) bands. NDBI ranges from −1 to +1 where high values indicate built up surfaces and negative values correspond to vegetation and waterbodies because of high NIR reflectance (Kshetri, 2018; Xu, 2007). Studies have linked densely urbanized areas with high dengue risk (Cox et al., 2007; Kularatne & Dalugama, 2022; Tsheten et al., 2021). A map was created using median NDBI from Landsat 8 OLI imagery (30 m resolution, USGS, 2023) from January‐December, 2023 with a 10% cloud coverage threshold (Figure S6d in Supporting Information S1). The following equation was used to calculate the NDBI which we adopted from Zha et al. (2003),

(1) $$\text{NDBI}=\frac{\text{SWIR}-\text{NIR}}{\text{SWIR}+\text{NIR}}$$

where:

SWIR = Surface reflectance in the shortwave infrared band
NIR = Surface reflectance in the near‐infrared band

#### 2.2.7 Distance to Open Water

Proximity to open water bodies promotes local humidity, enhancing *Aedes* mosquito and longevity (Abdullah et al., 2022; Monintja et al., 2021; Ridha et al., 2019). *Aedes* mosquitoes' respiratory systems lack regulatory mechanisms, leading to death when the humidity is low (<60%) as their bodily fluid quickly evaporates (Abdullah et al., 2022; Costa et al., 2010; Monintja et al., 2021). Areas within 1.25–1.5 km of freshwater sources have higher likelihood of human‐mosquito contact rates and greater dengue risk (Hsueh et al., 2012). Waterbody shapefiles (rivers, lakes, ponds, canals) were collected from the Local Government Engineering Department (Local Government Engineering Department, 2018) (Figure S8 in Supporting Information S1). Euclidean distance method in ArcGIS was used to generate a raster surface representing the shortest distance (m) from each pixel to the nearest waterbody (Figure S6e in Supporting Information S1). Dengue risk generally decreases with increasing distance from open waterbodies up to a maximum of 500 m (Tsheten et al., 2021) to 1.5 km (Hsueh et al., 2012). However, this relationship is not universally constant. In many urban settings, *Aedes* mosquitoes may breed in artificial containers such as household water storage, discarded tires, and small stagnant water sources rather than large open waterbodies (Barrera et al., 2011). Therefore, the proximity to natural waterbodies was used as a proxy for environmental conditions that may indirectly support *Aedes* habitats.

#### 2.2.8 Elevation

Elevation has a negative relationship with *Aedes aegypti* habitats (Lozano‐Fuentes et al., 2012). *Aedes aegypti* and *Aedes albopictus* typically thrive in warm lowland environments, therefore as elevation increases, temperature decreases, and the elevated cooler locations are unsuitable for vector breeding. Beyond disease ecology, elevation was considered a vulnerability element in hazard mapping (Gao et al., 2020; Sami et al., 2024) as it reflects differential adaptive capacity of people (Dow, 1992). Although elevation change is time invariant, its impact on overall risk is not direct like temperature, precipitation and stagnant waterbodies and it also works as an exposure factor (element of vulnerability) that refers to being at risk by potential hazards (Ahmed, 2021). The elevation raster was taken from NASA SRTM (Figure S6f in Supporting Information S1). Although Bangladesh is predominantly low‐lying, regions such as the Chittagong Hill tracts in southeast Bangladesh and Sylhet region in the northeast exhibit higher elevations (Figure S6f in Supporting Information S1), which may have distinct microclimate and accessibility characteristics that influence dengue transmission dynamics.

#### 2.2.9 Female Literacy

Educated women are more likely to have information about dengue risks and can be used as a variable to improve health care for their child and families (Zafar et al., 2021). Women in Bangladesh are primary care givers of the dependent members of the family (older adults and children). Female literacy can affect people's differential ability to cope with outbreaks. Zafar et al. (2021) found positive relationship between female literacy and adaptive capacity to dengue risk. Areas with lower female literacy rates may have less capacity to manage dengue outbreaks, which may cause potential loss to the community. Female literacy data was obtained from the BBS (2022) census (Figure S9 in Supporting Information S1) and rasterized in similar spatial extent and resolution with other predictors. Our study considered female literacy rate to have a negative functional relationship to dengue vulnerability (Figure S6g in Supporting Information S1) to conceptualize female literacy as an adaptive capacity factor as suggested by Zafar et al. (2021).

#### 2.2.10 Reported Dengue Incidence per 10,000 Population

Daily reported cases of dengue, deaths and number of hospitalizations were obtained from Directorate General Health Services (DGHS) from 2019 August to 2024 December. The daily cases were tabulated and processed to translate into district level data which was standardized into dengue cases per 10,000 population. Three temporal scales were used to process the dengue data which were monthly, seasonal, and annual. The district‐wise dengue incidence per 10,000 population was then used for spatial autocorrelation, hotspot analysis and as a validation layer for dengue risk surface.

### 2.3 Analytical Approach

#### 2.3.1 Spatial Autocorrelation and Dengue Hotspot Analysis

Spatial hotspots and cluster analysis along with spatial autocorrelation were conducted to model the spatial pattern (clustering, random or dispersed) of dengue cases in Bangladesh. Spatial clusters of dengue have been assessed using similar methods in other south Asian countries.

The data on dengue incidence per 10,000 population were spatially joined with district boundaries to analyze both the seasonal (winter, pre‐monsoon, monsoon, post‐monsoon) and annual (2019–2024) dengue hotspots. Global Moran's *I*, incremental spatial autocorrelation, Anselin Local Moran's *I* were employed to quantify spatial dependence and detect shifts in hotspot patterns over time. We used inverse distance as the spatial weight matrix for our analysis where neighboring districts exert greater influence than distant ones. We determined the distance threshold using incremental spatial autocorrelation to capture the peak clustering distance, ensuring that each district had at least one neighbor. The number of neighbors varied spatially based on district configuration. We identified hotspots where districts with high dengue incidence were surrounded by neighboring districts with similarly high values (Getis & Ord, 1992; Mitchell, 1999, 2005; Ord & Getis, 1995). Workflow and equations of spatial autocorrelation and hotspot analysis are in the supplementary document's method section (Figure S10 in Supporting Information S1).

To understand the seasonal and spatial shifts in dengue hotspots, we computed the Jaccard Similarity Index (JSI) (Jaccard, 1912; Nowak et al., 2022; Platt et al., 2018) to compare the seasonal hotspots between consecutive years across the study period (2019–2024). Districts were classified as hotspot (1) or non‐hotspot (0) based on statistically significant Getis‐Ord Gi* results (Gi_Bin > 0). The JSI was calculated as the number of districts classified as hotspots in consecutive years divided by the number of districts classified as hotspots in either year, for each seasonal pair across consecutive years. A JSI of 1 indicates identical hotspot geography between the 2 years, while a JSI of 0 indicates complete spatial turnover with no shared hotspot districts. This approach controls for seasonal variation by comparing the same season across years rather than seasons that follow one another (e.g., pre‐monsoon, monsoon) within a year. To further characterize the direction of hotspot reorganization, we quantified the proportion of hotspot‐seasons contributed by each geographic region (coastal/south; *n* = 17; south central delta; *n* = 7, central/peri Dhaka; *n* = 5, western; *n* = 11, eastern; *n* = 16) across the years.

### 2.4 Production of Weighted Dengue Risk Index (Analytical Hierarchy Process)

Analytical Hierarchy Process (AHP) was used to make optimal decisions regarding composite dengue hazard, vulnerability, and risk based on weighted priorities (Saaty, 1990). AHP has been utilized previously by numerous studies in the context of disease outbreak prediction such as dengue (S. A. Ali & Ahmad, 2018, 2019; Harsha et al., 2023; Tsheten et al., 2021), Diphtheria distribution (Fariza et al., 2021), COVID‐19 (Badillo‐Rivera et al., 2020; Mahato et al., 2020). The process involved creating pairwise matrices (according to Saaty (1990) in Table S2 in Supporting Information S1) for both themes (Figure S11 in Supporting Information S1), normalizing the comparison matrix using the normalized column sum method, and calculating weights for each criterion, preferences for alternatives, and overall priorities (Mu & Rojas, 2017). A consistency ratio was calculated using the consistency index and random index. Relative importance scores were based on previous AHP and dengue risk studies (Dom et al., 2016; Harsha et al., 2023; Jeefoo & Tripathi, 2011; Tsheten et al., 2021). Opinions of four national level health experts were taken to determine the final AHP weights. The four experts were chosen based on their professional experience in dengue related public health, epidemiology, and disease management in Bangladesh. Individual pairwise comparison matrices were aggregated using the geometric mean to obtain a consensus based weighting scheme. To ensure the reliability of their judgments in the AHP process, we evaluated the pairwise comparisons using the consistency ratio (CR), and only those within acceptable limits were considered in the final weighting. In addition, the variability among expert judgments was low, indicating a reasonable level of agreement. Details of the AHP calculation method are in the supplementary document's method section.

#### 2.4.1 Dengue Hazard Index (DHI)

Following Cutter's (1996) framework, we positioned hazard as a threat that has the potential to overwhelm people, property, and the environment. We used four components—temperature, precipitation, NDBI and distance to the waterbody as hazards. The random index (RI) was 0.90 (Table S3 in Supporting Information S1) because we employed four hazard components. Random index is a value that reflects the average consistency index of many randomly generated matrices of the same size. All subsequent calculations were done in Python Google colab environment with pyDecisions library (Pereira, 2024). The calculated consistency ratio (CR) for hazard was 0.05, which is within the acceptable threshold of 0.10, meaning the calculation was consistent. We follow the methodologies employed by Tsheten et al. (2021) and Harsha et al. (2023) for the pairwise matrix score (Table S4 in Supporting Information S1). The following equation was used to calculate the composite dengue hazard index (DHI) in ArcGIS pro raster calculator,

(2) $$\text{DHI}=\left(T\times W_{t}\right)+\left(P\times W_{p}\right)+\left(\text{NDBI}\times W_{b}\right)+\left(D\times W_{d}\right)$$

where:

*T* = Temperature (°C)
*P* = Precipitation (mm)
NDBI = Normalized Difference Built‐up Index
*D* = Distance to nearest waterbody (m)
$W_{t},W_{p},W_{b},W_{d}$ = Weights for temperature, precipitation, built‐up index, and distance to waterbody.

#### 2.4.2 Dengue Vulnerability Index (DVI)

According to Dow (1992), vulnerability to a hazard is due to the differential incapacity to deal with hazards, based on the position of groups and individuals within both physical and social spaces. Five factors were used as dengue vulnerability components, that is population density, distance to hospital, elevation, female literacy rate, and distance to major roads. The pairwise comparison matrix for the dengue vulnerability components was constructed (Table S5 in Supporting Information S1) and CR was calculated using an RI value 1.12 (see Table S2 in Supporting Information S1). As the CR value was less than 0.10, the pairwise comparison was considered consistent (Jeefoo & Tripathi, 2011; Tsheten et al., 2021).

The dengue vulnerability index (DVI) was calculated in ArcGIS pro as,

(3) $$\text{DVI}=\left(PD\times W_{pd}\right)+\left(HD\times W_{hd}\right)+\left(E\times W_{e}\right)+\left(FL\times W_{fl}\right)+\left(RD\times W_{rd}\right)$$

where:

*PD* = Population density
*HD* = Distance to nearest hospital
*E* = Elevation
*FL* = Female literacy rate
*RD* = Distance to major roads
$W_{pd},W_{hd},W_{e},W_{fl},W_{rd}$ = Weights for population density, distance to nearest hospital, elevation, female literacy rate, and distance to major roads.

#### 2.4.3 Dengue Risk Index

We conceptualized risk as a product of hazard and vulnerability. More specifically, it can be viewed as the probability of occurrence or the degree of loss of a specified element expected from a specific hazard (Dewan, 2013; Schneiderbauer & Ehrlich, 2004). Dengue risk index (DRI) was calculated from the following formula according to the definition by several studies (e.g., Blaikie et al., 2014; Jia et al., 2024; UN/ISDR, 2004; Wisner et al., 2004, 2012),

(4) DRI=DHI×DVI

where:

DHI = Dengue Hazard Index
DVI = Dengue Vulnerability Index

The AHP derived dengue hazard and dengue vulnerability layers were multiplied in raster calculator and the final risk map was prepared. There were four seasonal risk maps and one annual risk map. The value of the risk index was in a continuous scale from 0 to 1 and the risk areas were classified from “very low risk” to “very high risk” zones using equal interval.

#### 2.4.4 Unweighted Dengue Risk

Previous studies (e.g., Brooks et al., 2005; Patnaik & Narayanan, 2010; Ravindranath et al., 2011) used an unweighted method to assess dengue hazard and vulnerability. To avoid subjective bias inherent with AHP, we also created a dengue risk surface with equal weights for each component. This approach aimed to determine if components contributed equally to dengue outbreaks and risk in 2023. The final risk layer was created by combining rasterized layers with equal weights. Proper validation was needed to identify the best model for depicting dengue risk in 2023.

### 2.5 Association Between Dengue Incidence and Variables

To examine the adjusted associations between meteorological, environmental variables and dengue incidence, monthly dengue case counts across 64 districts were modeled using a negative binomial mixed‐effects regression (Hilbe, 2011; Ver Hoef & Boveng, 2007). The adopted model accounted for overdispersion in the count outcome and included a district‐level random intercept to capture the unobserved differences between the districts. Climate variables were lagged by 1 month and the analysis covered the period from August 2019 to December 2024, resulting in 4,160 district‐month observations. District population was included as an offset term using the natural logarithm of population, allowing estimates to be interpreted as incidence rate ratios (IRR). Each IRR compared the district level dengue incidence while holding all other variables in the model constant. Values above 1 indicate higher dengue incidence, and values below 1 indicate lower dengue incidence. The main predictors were 1‐month lagged temperature and 1‐month lagged precipitation. Both climatic variables and continuous district‐level covariates were standardized before model fitting. The model was adjusted for each season (lagged temperature and precipitation, linear time trend, NDBI, elevation, distance to water, distance to road, female literacy, and population density). For seasonal variables, winter was used as the reference because dengue case counts are at their lowest during this period. For standardized continuous variables, the IRR represented the expected change in the dengue incidence for a one standard deviation increase in the predictor. Multicollinearity among predictors was assessed using generalized variance inflation factors (GVIFs) (Table S8 in Supporting Information S1). Sensitivity analyses were performed because hospital distance and road distance were found to be highly correlated. Poisson and zero‐inflated negative binomial models were also additionally assessed for comparison. Models excluding each predictor separately were compared using AIC, and the model excluding hospital distance was selected as the final model.

### 2.6 Validation

We computed the receiver operating characteristic (ROC) and area under the curve (AUC) to assess the weighted and unweighted models' classification and diagnostic capacity to predict dengue risk. ROC analysis was performed using two binary classification thresholds. First, dengue incidence was dichotomized (diseased or non‐diseased) (Green & Swets, 1966; Metz, 1986) using the median value to evaluate the general discriminatory ability of the risk indices. Second, the 75th percentile was used to identify the extreme areas with the highest dengue risk. The true positive rate (TPR) and false positive rate (FPR) were plotted at various thresholds with AUC providing the summary measure of model performance (Metz, 1978). All analyses were conducted in Google Colab using Python's “sklearn.matrics” library. Model with higher AUC value was the most accurate for dengue risk in Bangladesh.

## 3 Results

### 3.1 Distribution of Dengue Cases in Bangladesh

In 2023, Bangladesh reported the highest dengue cases of all time (319,941 total cases), followed by the outbreaks of 2024 (147,425 total cases), 2022 (62,082 total cases) and 2019 (61,799 total cases). The long‐term average was approximately 2.5 dengue cases per 10,000 population between 2008 and 2024, which was considered a baseline for comparing annual anomalies (DGHS, 2024; Haider et al., 2021; M. S. Hossain et al., 2023; S. Hossain et al., 2023; IEDCR, 2019; Figure S12 in Supporting Information S1). While most years since 2008 had dengue cases below the long‐term average, the recent years, especially 2019, 2022, 2023, and 2024 had large spikes. Therefore, we can consider these 4 years as a dengue epidemic in the last 16 years. The number of cases in 2023 and 2024 have a higher positive anomaly than 2019 and 2022. For example, the number of cases in 2023 and 2024 was 8 times and 4 times higher respectively than the long‐term average (Figure S12 in Supporting Information S1). In 2023, dengue incidences peaked earlier than the other years starting from the onset of monsoon (1.76 cases per 10,000 population in July, 3.51 in August, 4.53 in September, and 4.41 in October) (Figures S13–S15 in Supporting Information S1).

**Figure 1:**
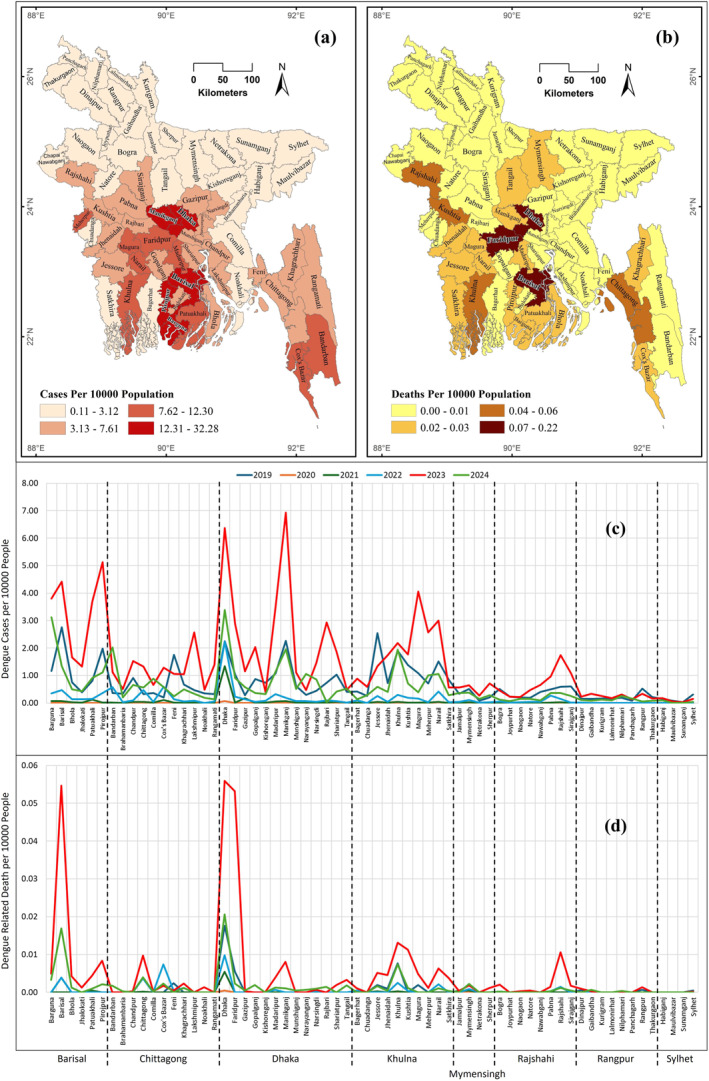
(a) Average annual dengue cases per 10,000 people, (b) Average annual dengue related deaths per 10,000 people in Bangladesh, (c) District wise monthly average dengue cases per 10,000 people, (d) Dengue related deaths per 10,000 people. The data range from 2019 to 2024.

The spatial distribution of average annual dengue cases in the past 6 years (2019–2024) was the highest in Dhaka (approximately 32.28 cases per 10,000 population) and Manikganj (22.73 cases per 10,000 population) districts, both within Dhaka division (Figure 1a). These districts recorded approximately 23 times higher average dengue cases per 10,000 population than the northern districts in the last 6 years. Compared to 2019, more than 4 people per 10,000 population were infected by dengue in both these districts in 2023, as shown in (Figure 1c). Southern coastal divisions such as Barisal (districts: Pirojpur, Barguna and Barisal) and Khulna (districts: Magura, Narail, Khulna) also showed elevated cases, peaking in 2023 before declining in 2024. By 2024, Dhaka's rate fell to 3.39 cases per 10,000 and Manikganj dropped from nearly 7 in 2023 to about 2 per 10,000 population in 2024. Out of the 8 divisions, districts of Rajshahi and Sylhet divisions on the northwest and northeast respectively had the least number of cases in 2019, 2023, and 2024 outbreaks.

Dengue related deaths were concentrated in Dhaka, Faridpur, and Barisal throughout all the years (Figure 1b). Barisal ranked second in 2023 in terms of dengue related deaths, but along with Dhaka, showed a threefold reduction in 2024. Other northwestern districts (Rajshahi, Kushtia) and southern districts (Khulna, Faridpur, Chittagong) (Figures 1b and 1d) also saw increased fatalities in 2023, that declined in 2024 noticeably. The scenario was similar in case fatality rate (%) for 2023 and 2024 (Figure S16 in Supporting Information S1). Overall death rates were the highest during 2023 post monsoon (Figure S17 in Supporting Information S1).

### 3.2 Global Moran's *I* and Overall Dengue Pattern

The overall annual dengue case pattern showed the strongest clustering (<1% chance of the result of random choices) during 2019 and 2023 with positive Moran's *I* close to 0.29 (*p* = 0.001) and 0.43 (*p* = 0.0002) respectively. Moderate clustering (Moran's *I*: 0.22; *p* = 0.01) was observed during 2024. Weak clustering (Moran's *I* = 0.04–0.11, *p* = 0.05) was observed in 2021 and 2022, meaning there was some spatial structure, but the clustering strength was marginal and only significant at the 10% level. Seasonally, clustering appeared during the 2019 post monsoon, and 2021 pre‐monsoon, whereas 2021 monsoon to 2022 post monsoon exhibited random patterns. Despite high dengue incidences in 2023, only winter season showed significant clustering (Moran's *I* = 0.18, *p* = 0.015). The strongest seasonal cluster occurred in 2024 winter (Moran's *I* = 0.43, *p* = 0.000004). The detailed seasonal and yearly results of Moran's *I*, *p* value and *Z* score are shown in Tables S6 and S7 in Supporting Information S1.

### 3.3 Local Moran's *I* Cluster and Outlier Analysis (Seasonal Dengue Hotspots and Coldspots)

**Figure 2:**
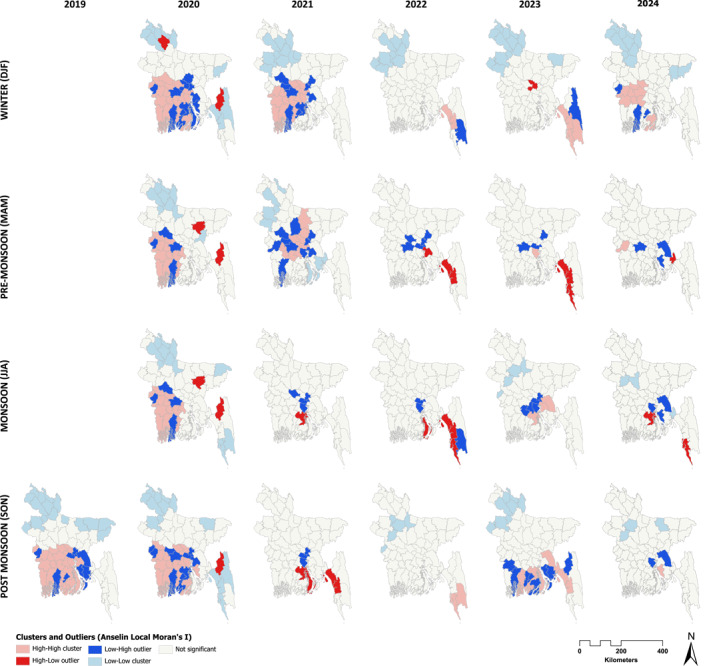
Analysis of seasonal (winter, pre‐monsoon, monsoon, post‐monsoon) dengue clusters and outliers.

Local Moran's *I* spatial autocorrelation was used to determine the statistically significant spatial cluster (high‐high; low‐low) and outliers (high‐low; low‐high) of dengue cases throughout the 64 districts of Bangladesh. Pink areas in Figure 2 symbolize high‐high clusters, indicating districts with high dengue cases that are surrounded by districts with high dengue cases. Light blue areas denote low‐low clusters or cold spots, where low dengue case regions are surrounded by districts with low dengue cases. Red areas indicate high‐low outliers (high dengue cases surrounded by districts with low dengue cases), while dark blue areas represent low‐high outliers (low dengue cases surrounded by districts with high dengue cases).

*Winter (December, January, and February)*: Each winter season from 2020 to 2023 showed a substantial shift of dengue hotspots from southwest coastal areas to south‐east districts of Chittagong and Cox's Bazar, except for 2024 when the high‐high clusters reemerged to southwest districts such as Jhenaidah, Gopalganj, Magura, Faridpur, and Patuakhali. The prevalence of hotspots in southwest mangrove and island districts was also prominent in 2020 and 2021, whereas the southern and southeastern districts became major hotspots for dengue active cases in the recent years of 2022–2024. Although the winter months in 2020 and 2021 showed more hotspots (high‐high) clusters, several districts on the southwest coastal region turned into statistically non‐significant hotspot in 2022, 2023 and 2024. The Getis Ord hotspot analysis shows persistent hotspots in Dhaka Barisal corridor throughout the winter seasons from 2019 to 2024 with 2023 showing a wintertime resurgence (Figures S18 and S20 in Supporting Information S1). The months of December and January showed significant increase of hotspots in the south coastal districts out of the three‐winter designated months over the last 6 years (Figure S20 in Supporting Information S1).

*Pre monsoon (March, April, and May)*: Dengue cases during the pre‐monsoon season showed a spatial shift from the hotspots from southwest coastal areas to central and southeast districts, similar to the winter season. There is a significant increase of high‐low outlier in Chittagong and Cox's Bazar during this season starting from 2022 as they turned into new hotspots. Dhaka ‐ Chittagong corridor is the primary zone for pre monsoon dengue clustering as evident from 2022 to 2024 seasonal dengue clusters, acting as early warning phase preceding larger monsoon outbreaks. The Getis Ord hotspot analysis (Figures S18 and S21 in Supporting Information S1) found more than 95% of statistically significant hotspots in the central Dhaka district and its surrounding districts such as Shariatpur during 2022 and 2023. Pre monsoon season in 2023 marked a major shift from a central to dual core structure (Barisal‐Chittagong), whereas in 2024 the clustered patterns were prominent persistently in the central districts. The northern districts constantly remained cold spots during pre‐monsoon months. March and April of 2023 showed the most significant southern shift of dengue hotspots (Figure S21 in Supporting Information S1).

*Monsoon (June, July, and August)*: The monsoon season in 2020 had the highest number of significant hotspots or high‐high clusters in the southwest districts of Satkhira, Khulna, Narail, Jessore. The low‐low cluster or cold spots were more prominent in northwest Dinajpur, Nilphamari, Rangpur, Jamalpur and Bogra districts. Starting from 2022, the reconcentration of dengue hotspots in southeastern region can be noticed as Bhola, Barisal, Chittagong, Bandarban and Cox's Bazar experienced higher dengue cases being surrounded by districts with low dengue cases. The cluster and outlier analysis shows Comilla, Chandpur and Barisal to have significant high‐high clusters in 2023 and the hotspots analysis include the associated districts Dhaka, Munshiganj and Narayanganj having dengue hotspots with 90% confidence level. Among the monsoon months, July and August had the highest increase dengue hotspots (Figure S22 in Supporting Information S1). Compared to July 2023, July 2024 had fewer dengue hotspots (7 districts in 2024 vs. 22 districts in 2023) in the central‐southeastern corridor (Figures S18 and S22 in Supporting Information S1).

*Post‐monsoon* (*September, October, and November*): The central districts (Dhaka, Manikganj, Rajbari, Faridpur, Shariatpur, Madaripur) and the southwestern districts (Satkhira, Khulna, Pirojpur, Patuakhali, Barguna and Bhola) had significant high‐high dengue clusters in both 2019 and 2020. In 2023, the number of significant peak dengue clusters increased compared to the previous two years. Bagerhat, Chittagong, Comilla districts which were previously outliers or non‐significant turned into statistically significant high‐high dengue hotspots. Overall, dengue clusters were prevalent in 13 districts during 2023 post monsoon compared to 4, 8, and 1 district in 2022, 2021, and 2024 post monsoon respectively. All three months of September, October, and November had significant increase of dengue hotspots in 2023 (Figures S18 and S23 in Supporting Information S1).

### 3.4 Seasonal Shift of Dengue Hotspots

The JSI analysis revealed that the geography of dengue hotspots was spatially dynamic across all four seasons (JSI range 0–1, Table S9 in Supporting Information S1). The post‐monsoon season showed the most pronounced reorganization: the similarity was high between 2019 and 2020 (JSI = 0.83, 25 shared districts) but declined sharply and did not recover in subsequent years (JSI = 0.21, 0, 0.06, and 0.11 for 2020–2021 through 2023–2024), with 13 new districts emerging as hotspots in 2022–2023 compared to only one shared districts. Regional analysis confirmed that the coastal district share of post monsoon hotspot seasons increased from 39% in 2019 to 92% in 2023, while western districts, which accounted for 21%–29% of post‐monsoon hotspot in 2019–2020, contributed zero hotspot seasons from 2021 onwards (Table S10 in Supporting Information S1). Winter season showed a similar but less reliable pattern, with coastal districts comprising of 100% of hotspot districts in 2022 and 2023, although the total district counts were low. Pre‐monsoon and monsoon season showed limited coastal shift, instead both seasons exhibited complete disappearance of hotspots in the western districts after 2020 and redistribution toward central and peri‐urban districts surrounding Dhaka and south‐central delta districts.

### 3.5 Yearly Shift of Dengue Hotspots and Coldspots

The local Moran's *I* result for the yearly overall dengue clusters (Figure S24 in Supporting Information S1) in Bangladesh has shown that there was a slight positive spatial autocorrelation in 2019 and *R* ^2^ value of 0.29. In 2020 and 2021, the local Moran's *I* result indicated an absence of significant spatial autocorrelation, as reflected by a very low *R* ^2^ value (0.01). Even though an outbreak of dengue occurred in 2022, there was little indication of rising spatial autocorrelation (*R* ^2^: 0.09) compared to the previous two years. There is a return of positive spatial correlation in 2023 which was stronger than the 2019 dengue outbreak (*R* ^2^: 0.32). Compared to 2019 and 2023, 2024 as an epidemic year showed weak positive spatial autocorrelation (*R* ^2^: 0.16).

**Figure 3:**
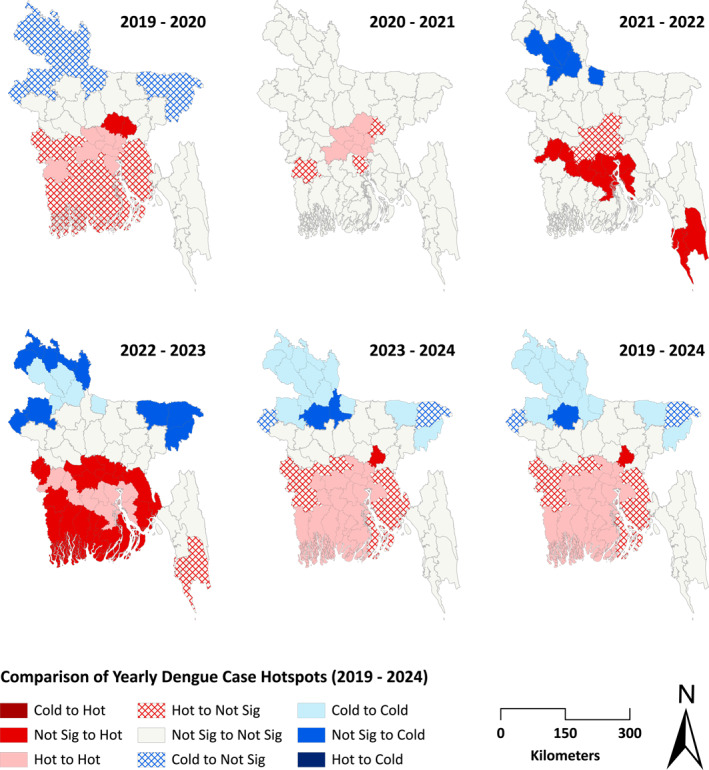
Yearly comparison of dengue hotspot.

From 2019 to 2020, there was a strong contraction of hotspots as majority of the southern coastal districts (Khulna, Bagerhat, Satkhira, Pirojpur, Bhola) turned into statistically non‐significant areas with no clusters from significant hotspot zones (99% confidence) in 2019 (Figure 3). Only two districts surrounding Dhaka turned into hotspots in 2020 while most of the districts turned into non‐significant hotspots. In 2020 and 2021 significant spatial shifts in the dengue hotspots were not observed. Early re‐emergence of dengue hotspots occurred in 2022 as 11 new districts became dengue hotspots, compared to the 6 hotspots in 2021. Among them, three districts (Jhenaidah, Magura, Narail) belong to southwest Bangladesh, four districts (Gopalganj, Madaripur, Shariatpur, and Chandpur) in central Bangladesh, 2 districts in southcentral (Barisal and Lakshmipur) and two in southeastern region (Cox's Bazar, and Bandarban). In 2023, 17 coastal districts such as Meherpur, Chuadanga, Rajbari, Faridpur, Dhaka, Munshiganj, Narayanganj, Comilla, Noakhali, Bhola, Patuakhali, Barguna, Pirojpur, Bagerhat, Khulna, Satkhira, and Jessore became new hotspots in 2023. A gradual stabilization of hotspots was observed in central and southern districts from 2019 to 2024. Hot‐to‐not significant transitions are prominent in districts such as Meherpur, Manikganj, Chuadanga, Jessore, Bhola, Comilla, and Noakhali, indicating a decline in dengue clusters in these areas. The northern districts remained cold‐to cold and cold‐to‐not significant. When we compared 2019 to 2024, there were persistent hotspots in 2024 in Dhaka and Barisal with an addition of Narsingdi district turned into a hotspot from being non‐significant while northern and northwestern districts showed persistent cold spots. It is evident from this analysis that no substantial improvement occurred in the last 5 years in those previously affected dengue hotspots in the central and south coastal districts. Yearly Getis‐ord Gi* dengue hotspots from 2019 to 2024 are shown in Figure S19 in Supporting Information S1.

### 3.6 Dengue Risk Composites (Weighted and Unweighted)

#### 3.6.1 Weighted Dengue Hazard, Vulnerability and Risk

**Figure 4:**
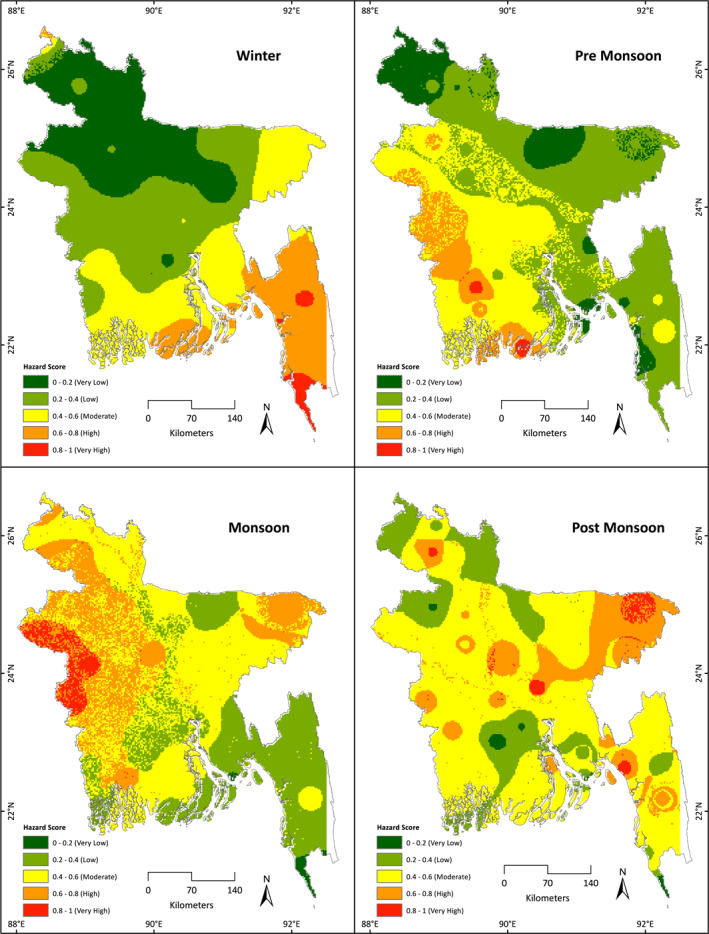
Dengue hazard during winter, pre‐monsoon, monsoon and post‐monsoon seasons as determined by the analytical hierarchy process weighted method.

*Dengue Hazard (DH)*: While the high and very high zones were predominantly found in the southeast Chittagong Hill tracts during winter, the higher hazard zones shifted toward the southwest and central districts in pre‐monsoon (Figure 4). Monsoon season had the highest number of districts that were in the moderate to very high dengue zones encompassing the central, western, and northern districts.

**Figure 5:**
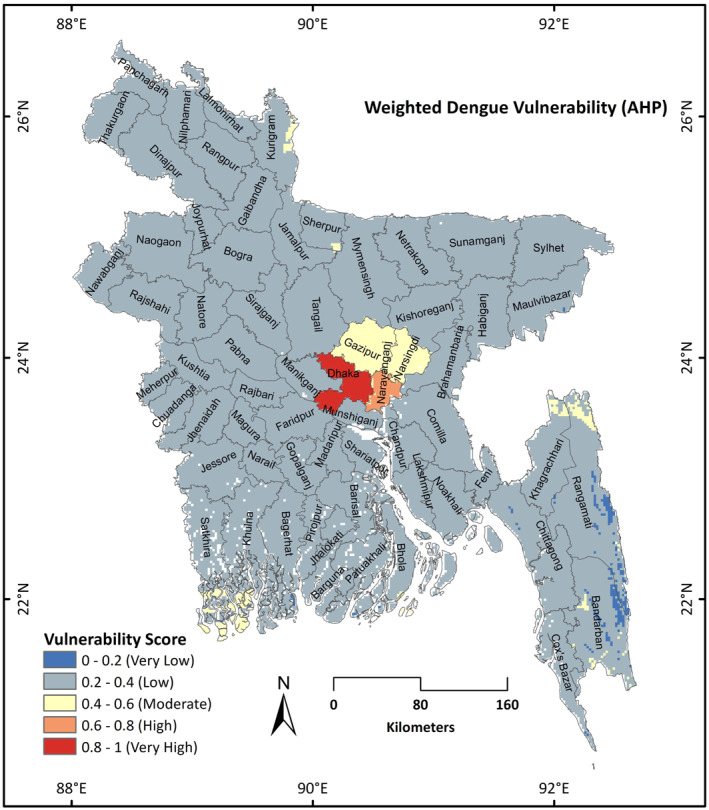
Dengue vulnerability across the districts as determined by the analytical hierarchy process weighted method.

*Dengue Vulnerability (DV)*: The weighted dengue vulnerability map showed the central district of Dhaka to have the highest vulnerability score (0.8–1), taking both the social and indirect factors such as population density, female literacy rate, elevation, distance to hospital, distance to hospital into account (Figure 5).

**Figure 6:**
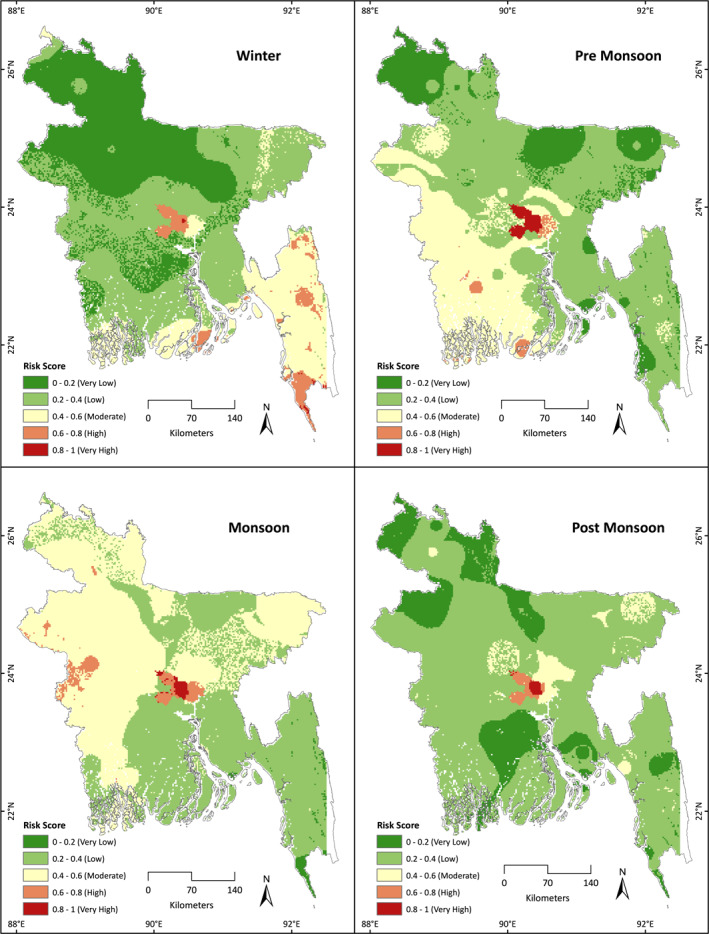
Seasonal dengue risk across all districts as determined by the analytical hierarchy process method.

*Seasonal Risk of Dengue*: During the winter season, the dengue risk index (DRI) showed a generally low risk pattern across most of the country in more than 30 districts, especially in the northwestern districts such as Naogaon, Rajshahi and Dinajpur. Yet, 15 districts in total were categorized as high and very high dengue risk zones during the winter (Figure 6). Among these districts, Cox's Bazar, Rangamati and Dhaka were in the top three districts with very high dengue risk according to the risk score assigned. More than 1,350 km^2^ in Dhaka district (approximately 92.5% of the total land area in Dhaka), 1,056 km^2^ in Rangamati and approximately 1,100 km^2^ in Cox's Bazar were in high dengue risk zones (Figure S25 in Supporting Information S1).

The transition from winter to pre‐monsoon season led to an increase in risk, with more districts in the west going from low dengue risk zone to moderate dengue risk zone (Figure S26 in Supporting Information S1). A total of 18 districts were in the high and very high‐risk zones. Among them, Dhaka had the most extensive area (approximately 1,390 km^2^) as very high risk and 25 km^2^ as high‐risk zone. The neighboring district Narayanganj exhibited high risk (433 km^2^) and very high risk (9.49 km^2^) (Figure S27 in Supporting Information S1). Furthermore, the south coastal districts of Khulna, Patukhali, and Barguna had broader areas in high‐risk zone. The hilly districts of Chittagong, Bandarban, Rangamati and Cox's Bazar transitioned into low‐risk zones during pre‐monsoon from moderate to high risk during winter. Conversely, Mymensingh, Dinajpur, Netrokona, Sylhet and Thakurgaon were the top five districts with significantly low dengue risk.

During the monsoon season, the risk increased significantly compared to the winter and pre‐monsoon seasons with 22 districts in the high and very high‐risk zones. The districts with extensive areas in the high‐risk zone were Dhaka, Narayanganj, Kushtia, Meherpur, Pabna, and Natore. Approximately 800 km^2^ in Dhaka was in high‐risk zone and 600 km^2^ in very high‐risk zone (Figure S28 in Supporting Information S1). The neighboring districts of Dhaka transitioned into moderate to high‐risk zones from low risk during pre‐monsoon. Monsoon season had the highest areas in moderate to high‐risk zone, making this season the riskiest season for dengue. Districts of the northeast Sylhet division turned into moderate risk zone.

The post‐monsoon season exhibited a sharp decrease in dengue risk levels compared to the monsoon season. Fewer number of districts were in the moderate (yellow) risk zones than before, however the risk remained elevated in Central Dhaka and its neighboring districts. A total of 7 districts including Dhaka, Narayanganj, and Manikganj exhibited high dengue risk. More particularly, 1,020 km^2^ (69.6%) in Dhaka was identified as a high‐risk zone and approximately 370 km^2^ (25.2%) was in very high‐risk zone (Figure S29 in Supporting Information S1).

#### 3.6.2 Unweighted Dengue Hazard, Vulnerability, and Risk (Equal Weight Model)

The unweighted dengue hazard, vulnerability and risk maps were created using equal priority for all the social, environmental, and climatic factors to look at possible dengue risk without any bias. The unweighted model generated similar patterns of dengue hazard, vulnerability and risk surface that are more widespread (Figures S30–S32 in Supporting Information S1).

#### 3.6.3 Validation and Comparison of Weighted and Unweighted Models

When median threshold was considered, the AUC value for the AHP weighted dengue risk model was 0.75 which means that the model can distinguish between two classes, risk versus no risk (Figure S33 in Supporting Information S1). On the other hand, the unweighted dengue risk model's AUC value, represented by blue curve, was only 0.55 which is slightly better than the random model to discriminate between risk or no risk areas. The curve is much closer to the diagonal, and the model likely fails to correctly predict the risk areas compared to the weighted AHP model.

When the 75th quantile threshold was considered, the AHP weighted risk model's AUC value decreased to 0.64. For the unweighted model, it further decreased to 0.50. It indicates that the risk indices were better at distinguishing relatively higher‐risk districts overall than isolating only the most extreme areas with the highest risk.

### 3.7 Association Between Dengue Counts and Seasonality

The dengue counts were highly over dispersed, with a variance to mean ratio of 7530.9, supporting the use of a negative binomial model. The negative binomial model provided a substantially better fit (based on AIC) than the Poisson model and slightly outperformed the zero inflated negative binomial model (Table S11 in Supporting Information S1). Therefore, the final model was the negative binomial mixed effects model which did not include the distance to hospital as a variable. Previous‐month temperature was strongly and positively associated with dengue incidence. A one standard deviation increase in the lagged temperature was associated with four (IRR = 4.168, *p* value <0.001) fold higher dengue incidence. Previous‐month precipitation was also positively associated with dengue incidence, with a one standard deviation increase in lagged precipitation corresponding to approximately two‐fold higher dengue incidence (IRR = 1.621, *p* value <0.001). Population density was positively associated (IRR = 1.77, *p* value = 0.001) with dengue incidence, while the COVID‐19 period was associated with substantially lower reported dengue incidence (Table S12 in Supporting Information S1). During the pandemic (the COVID‐19 period), dengue incidence was 97% lower than the latter years after the pandemic (IRR = 0.03, *p* < 0.001). One standard deviation increase in NDBI was associated with lower dengue incidence (IRR = 0.620, *p* = 0.012). The association with other covariates such as elevation, distance to water, distance to road, female literacy were not statistically significant (Figure S34 and Table S12 in Supporting Information S1).

The seasonal analysis of the model (Table S12 in Supporting Information S1) showed that dengue incidence during the pre‐monsoon season was much lower (IRR = 0.004, *p* < 0.001) than in winter after controlling for climate, population, district effects, COVID‐19 period, and other covariates. Dengue incidence during the monsoon season was also about 47.3% lower than in the winter season (IRR = 0.527, *p* = 0.002). The post‐monsoon dengue incidence was about 31.9% higher than the winter season however this result was not statistically significant (*p* = 0.139). Overall, the negative binomial fixed effect model captured the broad dengue pattern reasonably well, with an observed predicted correlation of about 0.71 (Figure S35 in Supporting Information S1).

## 4 Discussion

Bangladesh has seen a sharp increase in dengue infections in 2023, with cases surpassing 2019's record. Compared to the 2019–2022 period, the nationwide dengue peak occurred much earlier in 2023 (around June) with fatalities peaking in September and October. Our study assessed the spatiotemporal dynamics of dengue hotspots and dengue risk, to compare the nature and seasonality of risk and actual dengue clusters. Our results have shown that Dhaka and Manikganj had the highest annual average dengue cases per 10,000 people in the past 5 years. Both districts have high population density, urban land and low terrain facilitating elevated *Aedes* vector breeding behavior. Our results confirm that temperature and rainfall in the previous month are important predictors of subsequent dengue burden at the district month scale and consistent with previous studies (e.g., Cheong et al., 2013; Y. H. Lai, 2018; Malik et al., 2017; Ramachandran et al., 2016; Wangdi et al., 2018) that reported stronger climatic influences on dengue transmission. Although some studies (Focks et al., 2000; Ramachandran et al., 2016) have suggested that there is a thermal threshold of 34°C as high temperature dehydrate mosquitoes, shorten their lifespan and reduce egg viability (Tun‐lin et al., 2000), the number of cases in Dhaka were higher in 2023 even though the temperature during pre‐monsoon and monsoon season exceeded 34°C. These patterns likely reflect localized influences rather than strong linear relationships at the annual scale.

Our results also found population density to be positively associated with dengue incidence, consistent with increased transmission potential in densely populated areas, as also discussed in previous dengue studies (Gubler, 1998; Hsueh et al., 2012; Tsheten et al., 2021; Yue et al., 2018). This could be the reason why Dhaka is consistently a hotspot for dengue throughout the years and seasons. In contrast, higher NDBI values common in densely populated cities were associated with lower dengue incidence. Although dengue is commonly considered an urban associated disease, this inverse relationship could be due to differences in urban infrastructure, drainage systems, vector control practices or environmental conditions across districts. More densely built‐up areas may have fewer suitable mosquito breeding habitats compared with rapidly expanding peri‐urban environments where water accumulation and environmental management are less controlled.

There was a massive dengue surge in 2019 and extension of dengue hotspots in non‐endemic regions. The CFR was one of the lowest—0.17% (M. S. Hossain et al., 2023; S. Hossain et al., 2023) in 2019 compared to 0.54% CFR in 2013 (highest in the past 20 years). The difference in district level CFR could be attributed to accessibility to health care and high female literacy rate in Dhaka. Understanding the climatic and social components in these districts can also explain the differential severity. The possible contributing factor behind higher CFR values in southern coastal districts and some northern districts could be inaccessibility to hospitals, fewer hospitals and service providers, lower elevation and higher proximity to wetlands compared to other districts in the north which poses the risk of flooding, all of which afford these districts a lower adaptability to dengue fever. In 2024, Dhaka's CFR decreased to 0.60% possibly due to improved medical facilities and awareness after the highest epidemic in 2023.

Across seasons, post‐monsoon exhibited the greatest and most directional spatial shift (mean JSI = 0.24). The consistently low post monsoon hotspot similarity across four consecutive year pairs after 2020 suggests a genuine geographic reorganization rather than typical year to year fluctuation. The coastal shift is evident from the higher share of coastal districts in producing hotspots (92% by 2023 from 39% in 2019). Based on the pre‐monsoon's stable hotspot geography across consecutive years (mean JSI = 0.49), these hotspot districts might be more consistently determined by fixed environmental or structural factor. In the winter season between 2020 and 2022, the hotspot geography underwent geographic contraction. From 28 districts spread across southwestern coastal and western Bangladesh in 2020, hotspots collapsed to just three exclusively southeastern coastal districts; Chittagong, Cox's Bazar, and Bandarban in 2022, with Rangamati added in 2023 (JSI = 0.75 between 2022 and 2023, indicating these southeastern districts were stable across both years). A possible reason for this could be the increasing trend in winter temperature in Chittagong and Cox's Bazar in the recent years. For example, the average monthly temperature rose by about 2.22°C during winter months in 2023 relative to 2019's winter average monthly temperature (BMD, 2023). A new development of dengue hotspot during winter months could also be attributed to other factors such as increasing Rohingya settlement in Cox's Bazar. These remote indigenous communities are typically less prepared during sharp increase in dengue infections (Brierley et al., 2014; Sarker et al., 2024) combined with the limited accessibility to nearest health care centers in those settlements. In 2024, the southeastern coastal cluster disappeared, replaced by south‐central delta districts, suggesting continued geographic dynamism rather than stabilization of hotspots. The shift of dengue hotspots toward coastal regions is attributed to increased persistence of heatwaves (Koons, 2023; Montu, 2024). According to a recent report, more than 95% of the dengue cases in June 2025 were reported outside of Dhaka, predominantly in the southern coastal region of Barishal, Barguna, Khulna, and Chittagong (The Daily Star, 2025). The seasonal shift that was detected in our result aligns with the reported pattern of dengue hotspot in 2025 and necessitates a shift of focus to coastal districts of Bangladesh for dengue mitigation.

In terms of both yearly and seasonal hotspot analysis, Dhaka is the persistent urban dengue hotspot which was also shown by previous studies (Bhowmik et al., 2023; Hossan et al., 2023; Kayesh et al., 2023). Dhaka's constant dengue clusters can be explained by the massive heatwave in 2023 that was most prominent in Dhaka with average day time temperatures between April to June increased by approximately 2.74°C in the past two decades (Koons, 2023). Other drivers could be poor drainage condition and stagnant water. This spatial and seasonal variability supports our earlier finding that climatic influences on dengue are not strongly captured through simple annual correlations but may operate at finer temporal and regional scale.

The lower caseloads during 2020 and 2021 could be the effect of COVID‐19 restrictions. The COVID‐19 surge placed most districts in lockdown, which induced social trauma and public's tendency to avoid hospitals. Due to some overlap of symptoms between dengue fever and COVID‐19 (fever, headache, nausea, and fatigue), the under‐counting of reported dengue cases cannot be ruled out. This is perhaps why dengue fatalities and infections started ticking upward in 2022. Following the return to post‐COVID normalcy, building and construction activities resumed, which could have contributed to a mosquito population peak in 2023 by providing more mosquito breeding grounds in the abandoned sites (Alam, 2020; Haider et al., 2023).

The northeast Sylhet region and nearby districts were consistently showing low dengue cases during the monsoon and post‐monsoon seasons. However, the risk prediction layer for both these seasons presents Sylhet division as moderate risk area, which might be due to the increased precipitation pattern in these regions during these seasons. More scrutiny is needed to specifically assess Sylhet region and why it had fewer dengue cases even after being in the moderate risk zone in monsoon and post monsoon. Varying reasons such as proper preparedness, good drainage condition, good accessibility to health care and under‐reporting of dengue cases could be the reason why actual cases are fewer than expected. Even though the seasonal analysis of dengue risk showed higher risk during the monsoon, the adjusted model estimated lower IRR for pre‐monsoon and monsoon relative to the winter season.

Although our study provides a comprehensive district wise dengue risk scenarios with actual seasonal shift of dengue clusters, there are several limitations. Our research data is coarse scale district level spatial data which might not capture the finer scale variation of dengue hotspots. There is a lack of finer scale hospital data at the upazila and city level. In addition, DGHS dengue data has an underrepresentation of the public and private hospitals for each district. The reported dengue cases represent more around 70 hospitals in the country, however, there are more than 5,000 healthcare facilities (public and private) hospitals in Bangladesh (Rannan‐Eliya, 2012). On top of that, hospitalizations are not reported consistently every day, making it difficult to analyze temporal dengue hotspots for a particular location. These inconsistencies in the geocoded hospital data set may mask out actual magnitude of dengue fever cases. There is reporting bias in addition to these inconsistencies as most cases are likely to be reported in Dhaka or urban areas as they have better access to healthcare and surveillance systems are more likely to detect and report cases. In the risk maps (Figure 6), remote regions in Rangamati and Bandarban districts show no data value. That's because the two Landsat 8 collection images we used to calculate the NDBI in 2023 had “no data value” in those regions. Again, interpolation and raster‐based processing of variables may introduce uncertainty in the spatial representation of predictors. Furthermore, this research did not investigate dengue hemorrhagic fever or dengue shock syndrome because of the lack of data regarding specific dengue fever type. Even though DGHS publishes dengue data everyday but there are some temporal inconsistencies such as unavailability of 24‐hr dengue cases and deaths before August 2019. Although the surveillance system for dengue started way earlier in 2000, it only included serologically confirmed cases and only were Dhaka city based till 2019 (Sharmin et al., 2015). A robust analysis of the seasonal shift of dengue hotspots would have been only possible with additional years of data and we acknowledge that 6 years of data are insufficient to establish a long‐term trend, and the anomalously high case burden in 2023 may have influenced the extent of southern coastal hotspots in that year. Yet we show that the shift away from western districts and toward coastal districts began as early as 2021, two years before the anomalous 2023 transmission peak, indicating that the geographic reorganization was already underway independent of that anomalous year. Therefore, access to dengue case data is imperative for the researchers to properly illustrate the historical and future outbreaks for continued surveillance beyond the current study period. Temporally consistent dengue vector surveillance data will aid in diffusion based Aedes transmission modeling in future research. Furthermore, although our negative binomial mixed effect regression model captured dengue patterns reasonably well, it overestimated total case counts, suggesting over prediction. Finally, this research is based on the dengue case and death reports tabulated by the DGHS and can work as a guide to understand the broader picture of dengue hotspots and risk at the community scale.

Major cities in Bangladesh such as Dhaka, Chittagong and Khulna need more support and resources because of their growing population density, sewer system management which could have other public health challenges. Although the United States Federal Drug Administration has authorized Dengvaxia, a tetravalent dengue vaccine which is expected to be licensed in Bangladesh (Kumar et al., 2023), it is necessary to properly plan the equitable allocation of these vaccines among all classes of people in every districts. Organizations such as UNICEF have mobilized about 4.1 million USD towards dengue emergency response, but still a funding gap of 1.5 million USD persists (UNICEF, 2023). Thus, proper collaboration amongst public health stakeholders, government‐non government health care facilities, and climate scientists are needed to predict the seasonal outbreaks and manage it through vaccination and mobile emergency care. A major contribution of our study is the district level assessment of dengue dynamics across all 64 districts of Bangladesh over multiple transmission seasons, extending beyond the predominantly Dhaka focused analyses in previous studies. The broader spatial and temporal coverage enabled evaluation of regional variability in dengue burden as well as seasonal and interannual variation in hotspot distribution. Although the observed hotspot changes are only apparent in recent years after 2023, the findings suggest that dengue activity is not restricted to Dhaka and may increasingly affect climate‐sensitive coastal districts. This research presented the monthly, seasonal, and annual hotspots to induce proper prioritization of the current dengue risk areas. These current patterns are supported by the dengue risk zones to understand how risk surfaces can compare to the actual scenario. This holistic approach presented in our study is going to guide the future policy implementation and work as a background tool for coming epidemiological risk assessment research on a finer spatial scale.

## Conflict of Interest

The authors declare no conflicts of interest relevant to this study.

## Supporting information

Supporting Information S1

## Data Availability

The study utilized publicly available data sets to generate derived spatial products, dengue hotspots and dengue risk maps utilizing ArcGIS Pro (https://www.esri.com/en‐us/arcgis/products/arcgis‐pro/overview). Dengue daily case data was obtained from the Directorate General Health Services (https://old.dghs.gov.bd/index.php/bd/home/5200‐daily‐dengue‐status‐report). Shuttle Radar Topography Mission (SRTM) digital elevation model (DEM) was downloaded from USGS 1 Arc‐Second Global data set (USGS, Earth Resources Observation and Science (EROS) Center, 2018). The elevation data can be searched, previewed, and downloaded using the USGS Earth Explorer. The SRTM collections can be found under the Digital Elevation category. Road network data were obtained from the Roads and Highways Department (RHD) Bangladesh GIS portal (https://gis.rhd.gov.bd/portal/apps/webappviewer/index.html?id=e7e196a285bf4d789bb9d7dcbca7b0c1). The road network line data set can be previewed, downloaded, and extracted in ArcGIS Pro by going to the “Layer List,” then selecting the “Show Item Details” option of layer “RHD Road.” Climate variables (Maximum average temperature & total precipitation) were collected from Bangladesh Meteorological Department Data porta (https://dataportal.bmd.gov.bd/unk/). The step‐by‐step process of acquiring the data sets involves selecting surface meteorological data as data type with time range. (a) Choosing rainfall and temperature option from the variable drop down menu (b) selecting the stations (for our study, all stations were selected). This allows the user to calculate the price and submit data request to BMD by providing required reason for data requests. Population density and female literacy data were retrieved from Bangladesh Bureau of Statistics (https://bbs.gov.bd/pages/static‐pages/6922e0ff933eb65569e297dc). The data can be extracted from the Population & Housing Census report. Hospital facility locations came from the DGHS facility registry (https://hrm.dghs.gov.bd/public/facility‐registry). The list of the hospitals can be collected directly from this webpage in accordance with the data query. Open waterbody data were drawn from the Local Government Engineering Department (LGED) (https://data.humdata.org/dataset/bangladesh‐water‐courses). The shapefile of the waterbody can be directly downloaded from the Humanitarian Data Exchange (HDX) webpage. The processed geospatial and epidemiological data sets used for dengue risk modeling and hotspot analysis in this study are available at Zenodo (Jarin, 2025) with open access under a Creative Commons Attribution (CC BY 4.0) license. The *R* and Python code used for data preprocessing, modeling, and visualization are also archived at Zenodo (Jarin, 2025) with open access under a permissive open‐source license, and are mirrored on GitHub at https://github.com/njaringeo/denguepaper (njaringeo, 2026) for development and version control. All source data sets and software used are cited on the paper.
