# Supplementary material for: Spatiotemporal Dynamics of Dengue Risk in Bangladesh: A GIS Based Approach
Source: Geohealth. 2026 Jul 21;10(7):e2025GH001707. doi: 10.1029/2025GH001707 (PMC13387740; doi:10.1029/2025GH001707)
Supplement: Supplementary file 1 — Supporting Information S1 [file GH2-10-e2025GH001707-s001.docx]

***GeoHealth***

***Supporting Information for***

**Spatiotemporal dynamics of dengue risk in Bangladesh: A GIS based approach**

Nusrat Zahan Jarin^1, 2, *^, Abinash Bhattachan^2^, Obaidur Rahman^3^

^1^Department of Geography, University of California Santa Barbara, Santa Barbara, California

^2^ Department of Geosciences, Texas Tech University, Lubbock, Texas

^3^ Department of Geography, Oklahoma State University, Stillwater, Oklahoma

Corresponding Author: [njarin@ucsb.edu](mailto:njarin@ucsb.edu)

**Contents of this file**

Figure S1-S35
Table S1-S12

**Introduction**

This supporting document contains additional maps, graphs and tables of data and result sections. The study area and data section includes the study area and the epidemiological, social, environmental, and climatic variables that have been used to construct the overall risk of dengue for Bangladesh.

The result section is further divided into dengue case and distribution, dengue hotspots and spatial autocorrelation and dengue unweighted risk with area percentage for different districts.

# **Study Area and Data**


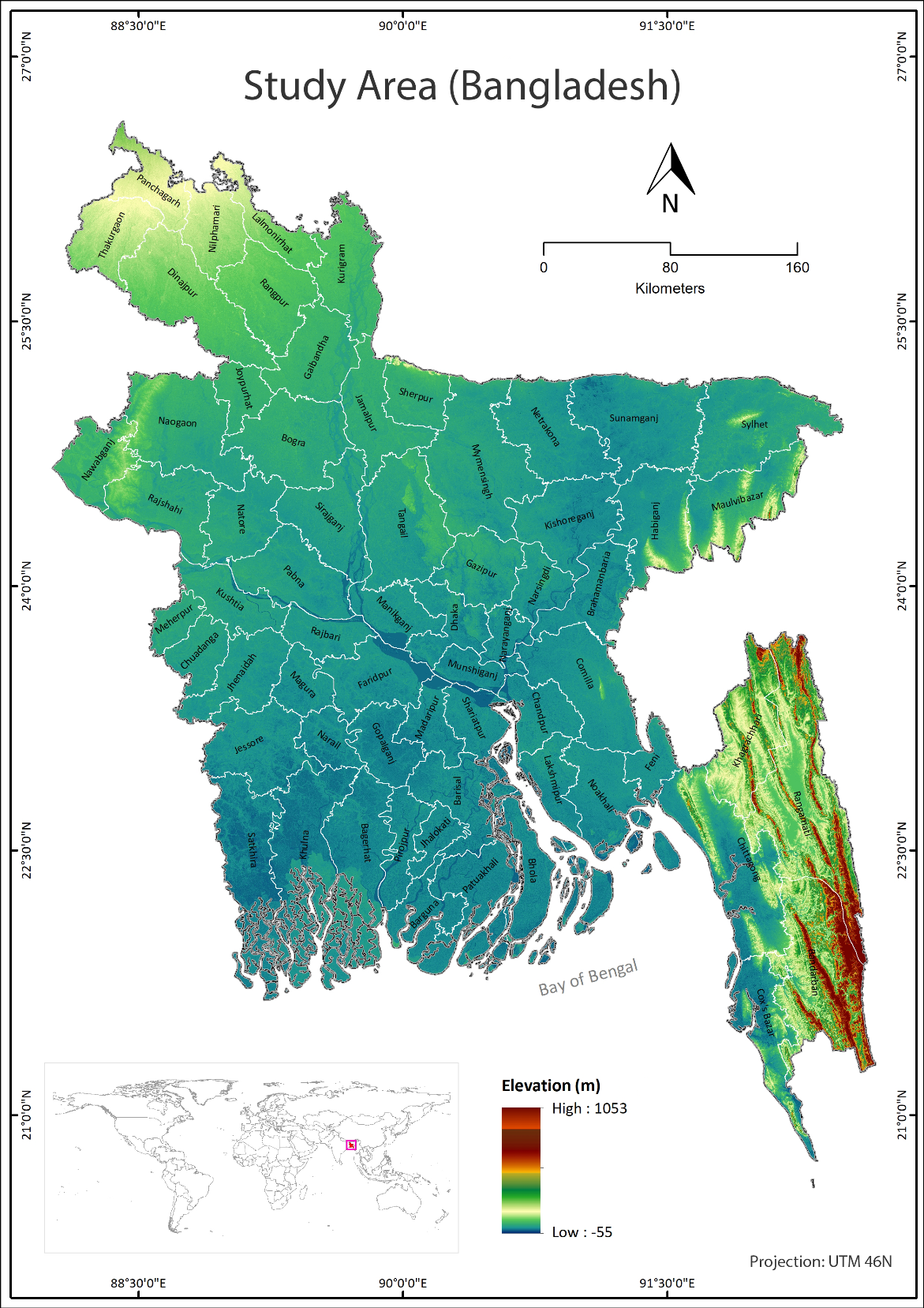


**Figure S1: Study area map of Bangladesh (Data source: NASA SRTM)**


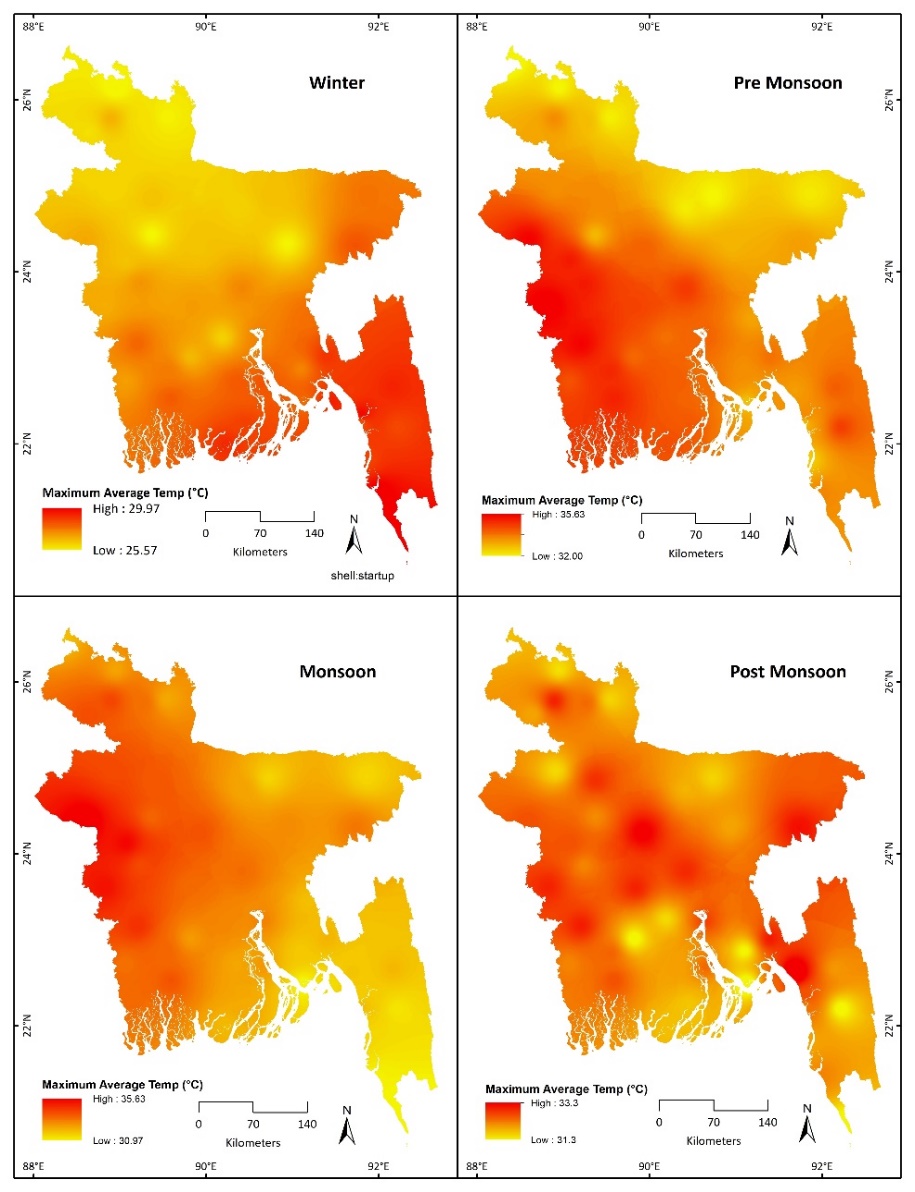


**Figure S2: Maximum monthly average temperature in 2023 (Seasonal) (BMD, 2023)**

***
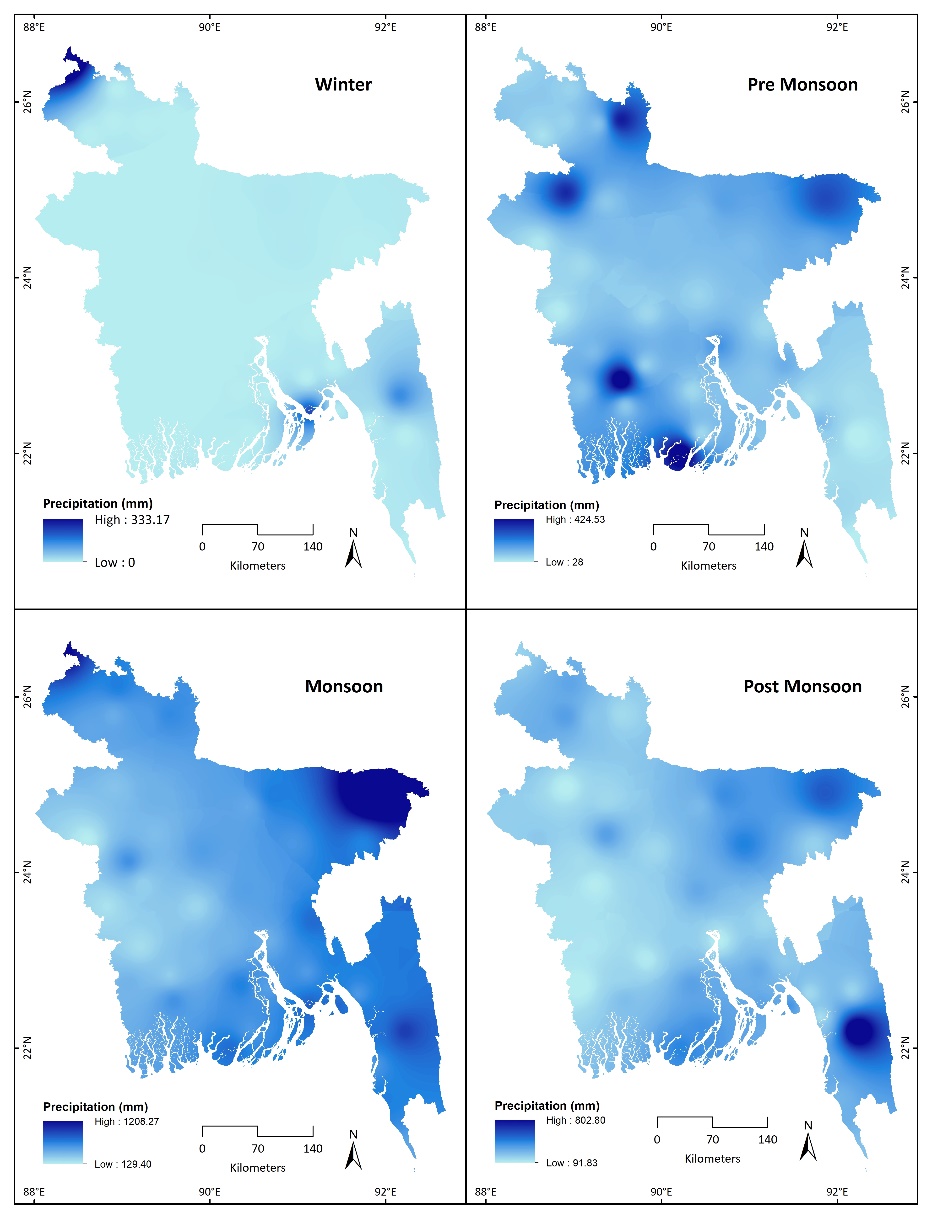
***

**Figure S3: Seasonal average precipitation in 2023 (Source: BMD, 2023)**


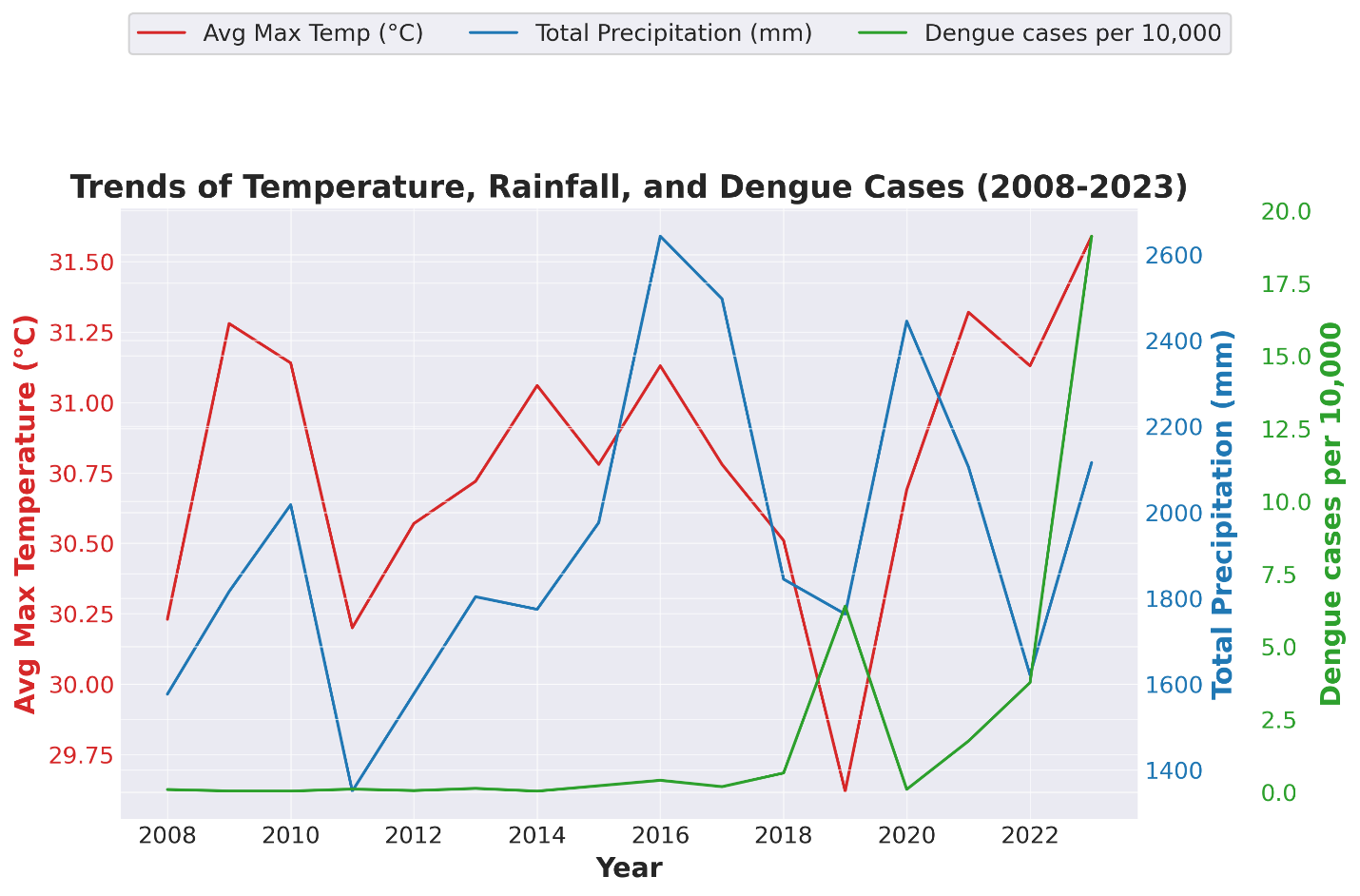

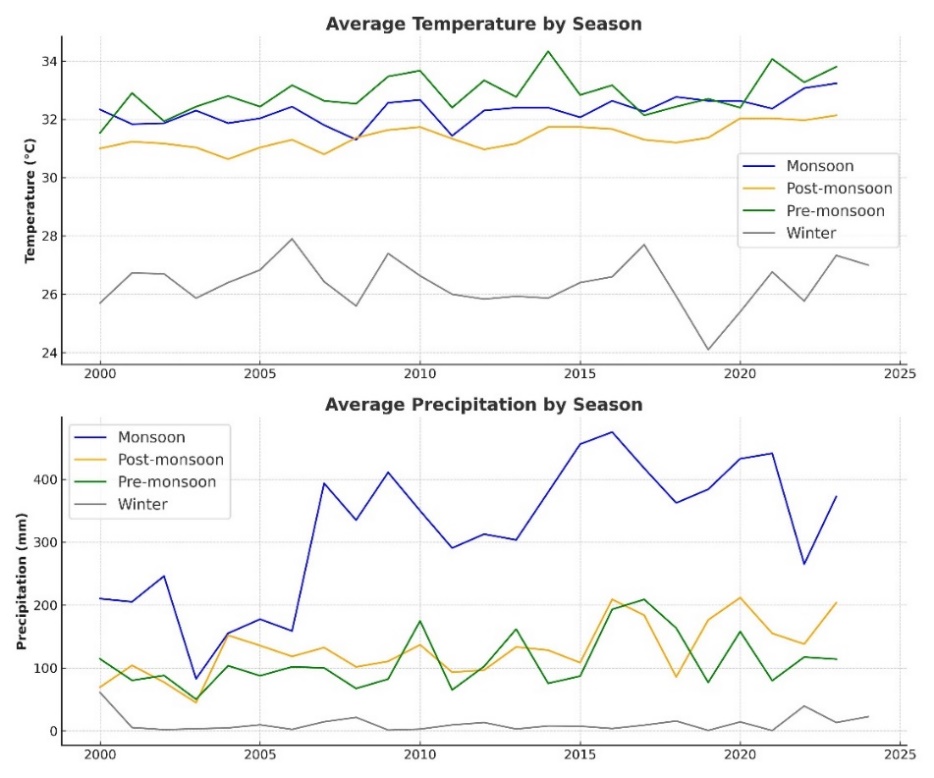


**Figure S4: Average temperature and precipitation trend 2000-2023 (BMD)**

**Figure S5: Trends of temperature, rainfall and dengue cases (2008-2023) (Datasource: BMD, 2023; DGHS, 2023)**


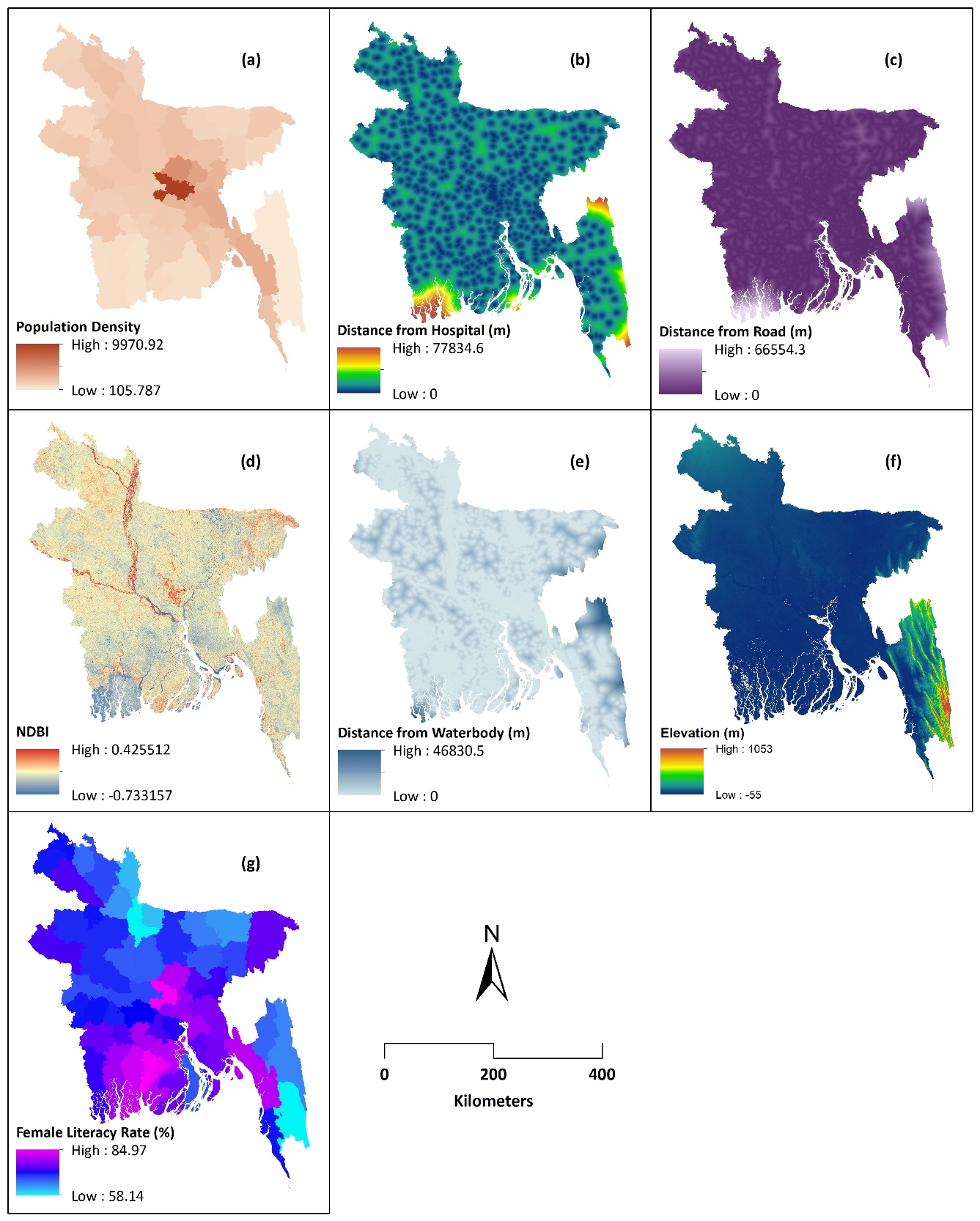


**Figure S6: Hazard and vulnerability components for dengue risk processed and analyzed in ArcPro**

***
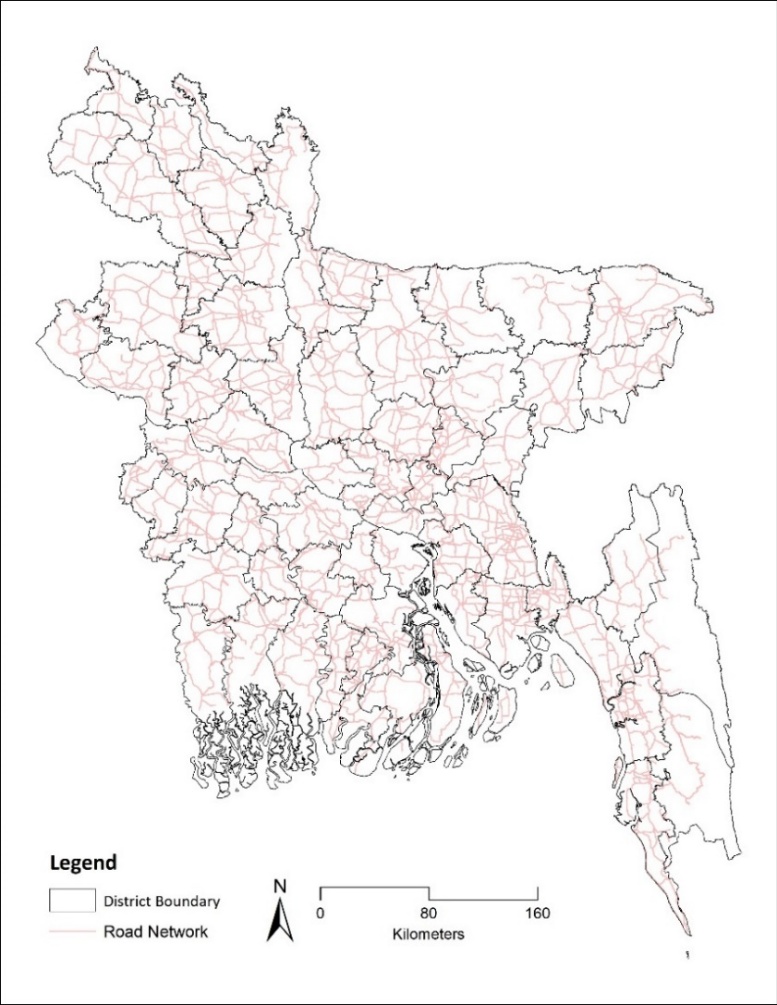
***

**Figure S7: Distribution of road network in Bangladesh (Source: RHD, processed and analyzed in ArcPro)**

*
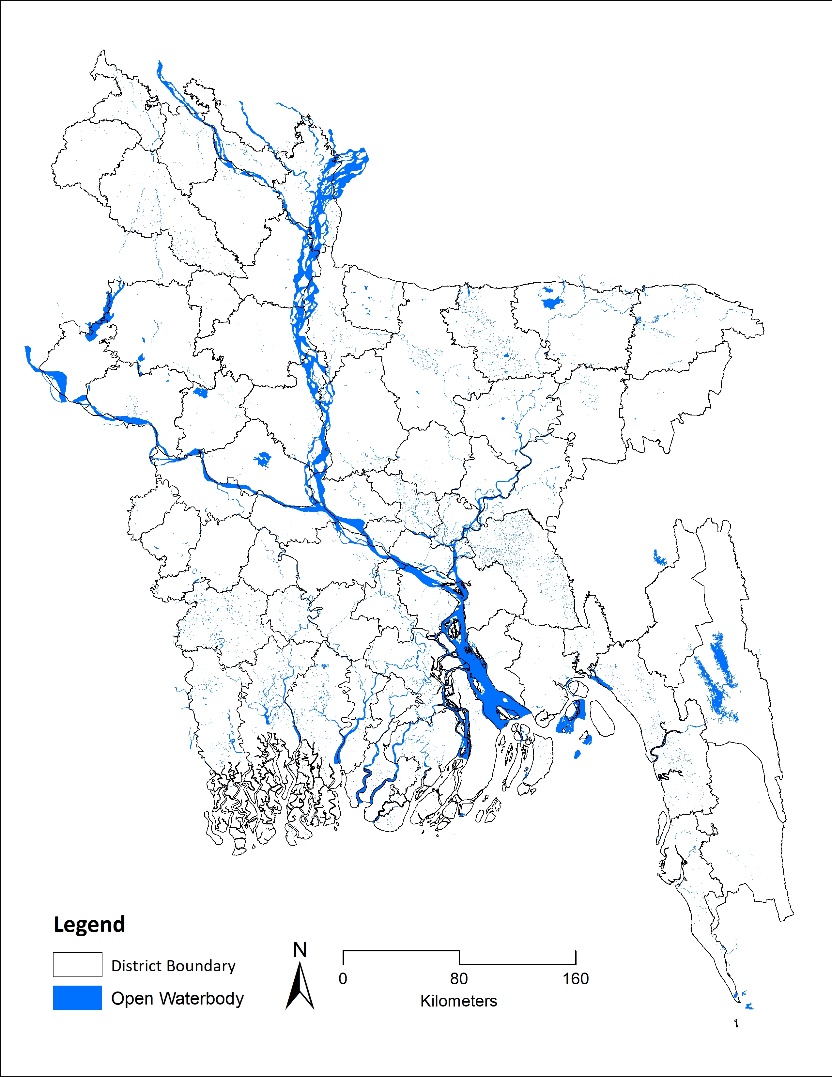
*

**Figure S8: Distribution of open waterbodies in Bangladesh (Source: BBS)**

**Figure S9: Average Female Literacy (%) (Source: BBS, 2021, processed and analyzed in ArcPro)**

# **Methods (Spatial Autocorrelation, Hotspots and Analytical Hierarchy Process)**

**Spatial Autocorrelation and Hotspot analysis**


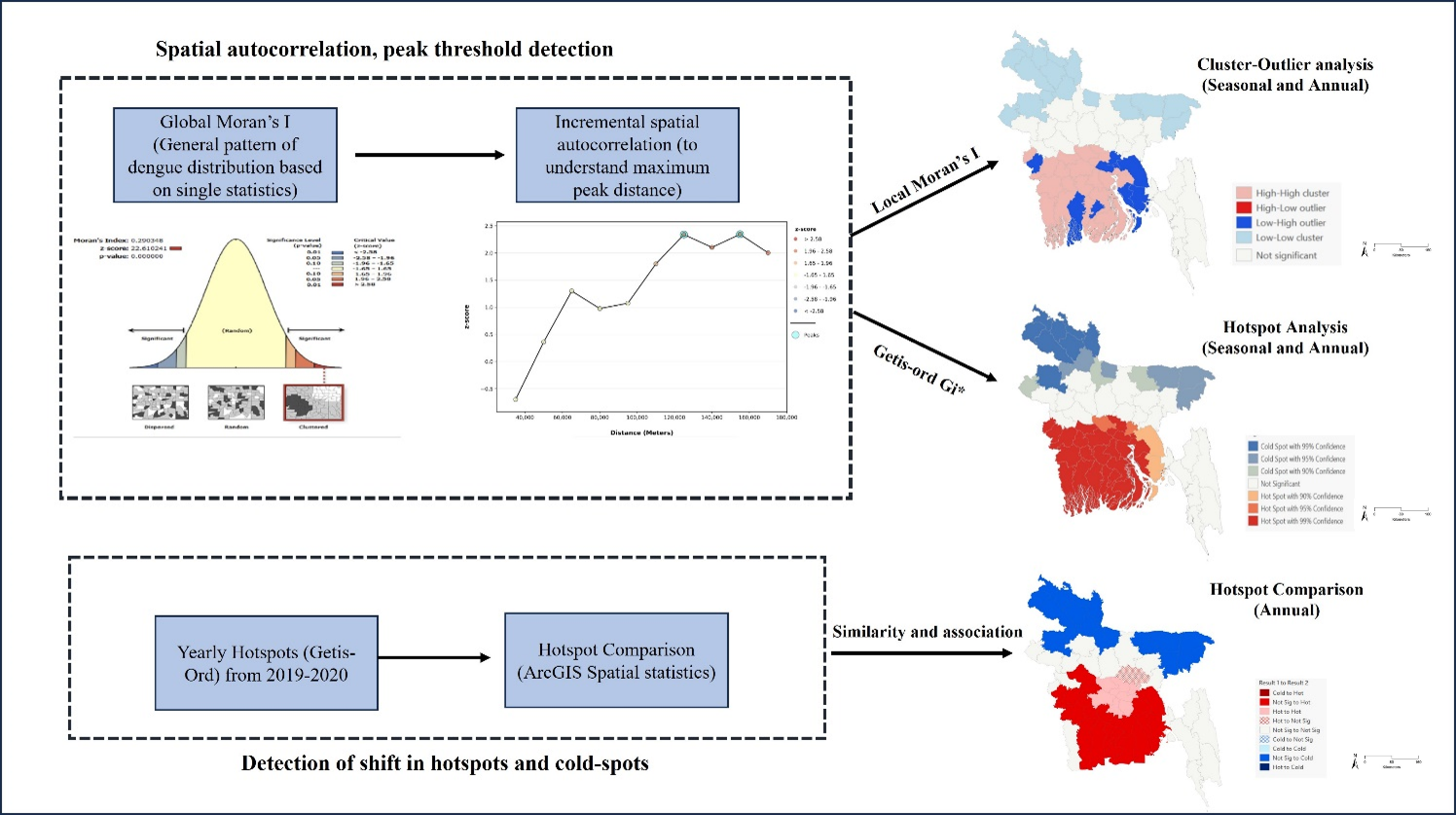


***Figure S10: Workflow for spatial autocorrelation and dengue hotspot analysis***

While global Moran’s I worked as a crucial tool for understanding the overall data pattern, it was still not sufficient to understand the spatial heterogeneity and local dengue pattern in Bangladesh. Local Moran’s I cluster and outlier analysis was used to understand the statistically significant dengue clusters and outliers. According to Anselin (1995); Mitchell (2005) in ArcGIS pro documentation, local Moran’s I statistics can be calculated as,

$$\begin{aligned} I_{i}=\frac{x_{i}-\overline{X}}{S_{i}^{2}}\sum_{j=1,j\neq i}^{n} w_{i,j}\left( x_{j}-\overline{X} \right)\#\left( 1 \right) \end{aligned}$$

where, Xi is the attribute for feature i, $\overline{X}$ is the mean of corresponding attribute, $w_{i,j}$ is the spatial weight between i and j. Si is calculated as,

$$\begin{aligned} S_{i}^{2}=\frac{\sum_{j=1,j\neq i}^{n} \left( x_{j}-\overline{X} \right)^{2}}{n-1} \#\left( 2 \right) \end{aligned}$$

where, n is the total number of features (districts). To calculate the Z score, equation (4) is used;

$$\begin{aligned} z_{I_{i}}=\frac{I_{i}-E\left[ I_{i} \right]}{\sqrt{V\left[ I_{i} \right]}} \#\left( 3 \right) \end{aligned}$$

and,

$$\begin{aligned} E\left[ I_{i} \right]=\frac{\sum_{j=1,j\neq i}^{n} w_{i,j}}{n-1} \#\left( 4 \right) \end{aligned}$$

$$\begin{aligned} V\left[ I_{i} \right]=E\left[ I_{i}^{2} \right]-E\left[ I_{i} \right]^{2} \#\left( 5 \right) \end{aligned}$$

The Getis-Ord local statistic is given as (Ord & Getis, 1995):

$$\begin{aligned} G_{i}^{*}=\frac{\left( \sum_{j=1}^{n} w_{i,j}x_{j} \right)-\bar{X}\left( \sum_{j=1}^{n} w_{i,j} \right)}{S\sqrt{\frac{n\sum_{j=1}^{n} w_{i,j}^{2}-\left( \sum_{j=1}^{n} w_{i,j} \right)^{2}}{n-1}}} \#\left( 6 \right) \end{aligned}$$

where, x_j_ is the attribute value for feature j, the spatial weight between feature j and i is denoted by w_i,j_, n is the total number of features (Pallathadka et al., 2022) and:

$$\begin{aligned} \bar{X}=\frac{\sum_{j=1}^{n} x_{j}}{n} \#\left( 7 \right) \end{aligned}$$

$$\begin{aligned} S=\sqrt{\frac{\sum_{j=1}^{n} x_{j}^{2}}{n}-\left( \bar{X} \right)^{2}} \#\left( 8 \right) \end{aligned}$$

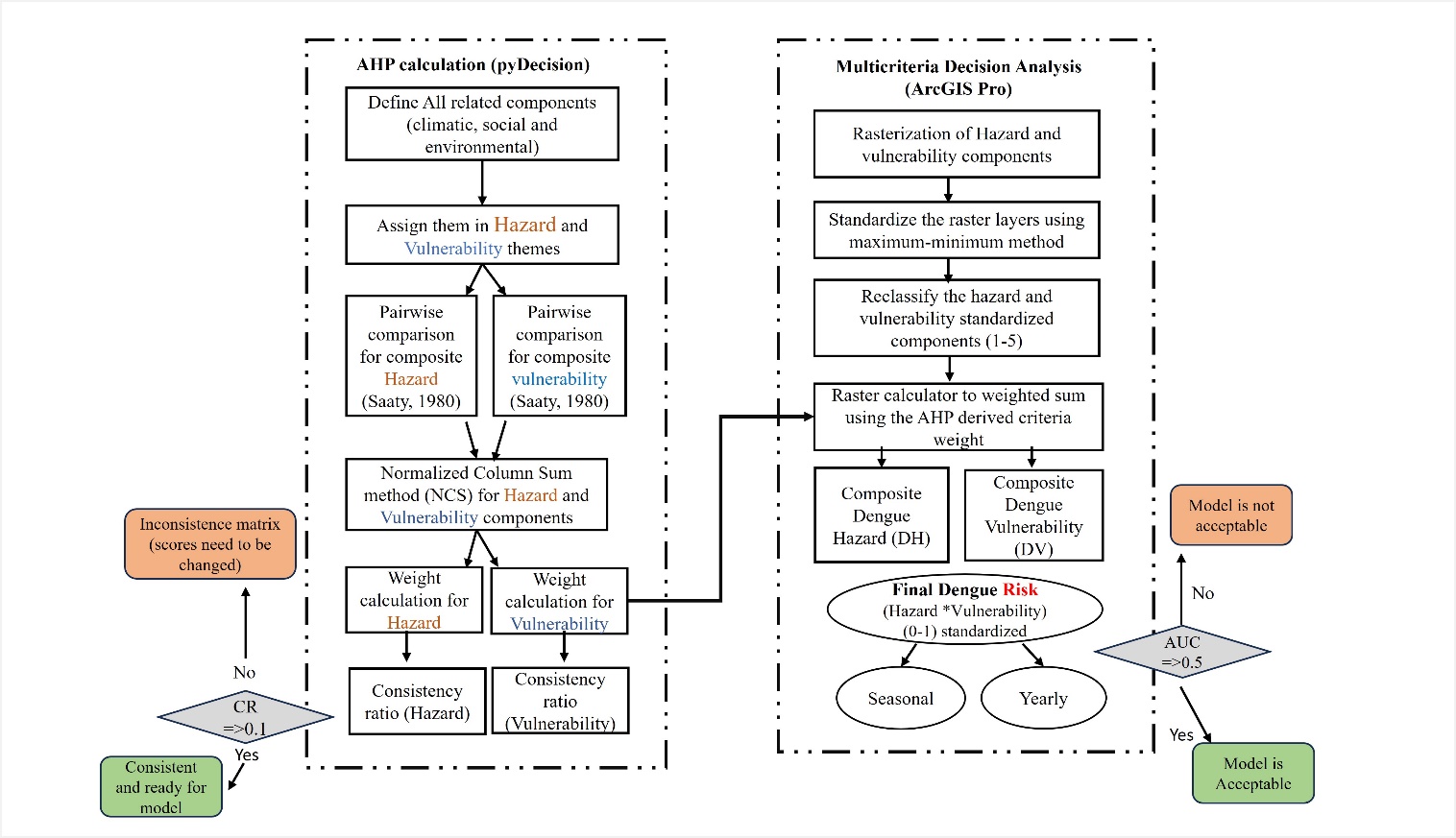
**Analytical Hierarchy Process**

***Figure S1: Workflow for AHP***

**Priority matrix and Eigen value**

Pairwise comparison matrix (PCM) was constructed following Saaty’s pairwise comparison table (in Table 1), mentioned in Ali & Ahmed (2018).

The rank of significance of one alternative to its next level was considered as inversely proportional, meaning that a rank of 1 or 3 resulted in a reciprocal weight of ½ or 1/3 respectively (Ali & Ahmed, 2018). The ranking priorities were prepared using the following formula in equation 10 (Ali & Ahmed, 2018):

Ax= λ_max_ X (9)

where, Ax is the comparison matrix of size (n x n), for n component (or called priority matrix), X is the Eigenvector of size n x 1 (or called priority vector) and λ_max_ is the Eigenvalue. This equation was used to derive the priority vector from the comparison matrix.

**Normalized Column Sum (NCS) method**

The NCS method was used for normalizing the comparison matrix. The equation is as follows:

$$\begin{aligned} NCS=\frac{A}{\sum a} \#\left( 10 \right) \end{aligned}$$

In equation 11, A represents the individual matrix value and ∑a is the sum of the matrix values in a column. So, this equation was performed for column wise normalization.

**Weight calculation**

$$\begin{aligned} W=\frac{A_{sc1}+A_{sc2}+\ldots+A_{scn}}{N} \#\left( 11 \right) \end{aligned}$$

where, W is the calculated weight, $A_{scn}$ is the NCS of every component, N is the number of components.

The NCS value of every criterion was checked to see if it was 1. For each column J in A,

$$\begin{aligned} \sum_{i=1}^{n} NCS_{ij}=1 \#\left( 12 \right) \end{aligned}$$

**Consistency ratio calculation**

The consistency ratio was calculated using the following formula devised by (Saaty, 1990)

$$\begin{aligned} CR=\frac{CI}{RI} \#\left( 13 \right) \end{aligned}$$

Here, CR is the consistency ratio, CI is the consistency index and RI is the random index. Consistency index usually is a measure calculated from the eigenvalue and the size of the matrix. The RI represents a value that reflects the average consistency index of many randomly generated matrices of same size. The values of RI were taken from the Saaty’s RI table (Table S2).

$$\begin{aligned} CI=\frac{\lambda\max-n}{n-1} \#\left( 14 \right) \end{aligned}$$

The CI value was calculated with equation 14

**Negative Binomial Mixed Effect Model**

Monthly dengue case counts were modeled using mixed effects count regression approaches with a population offset to estimate adjusted incidence rate ratios. Poisson, negative binomial, and zero inflated negative binomial model specifications were compared using Akaike Information Criterion (AIC). Because the dengue counts exhibited substantial overdispersion, the negative binomial mixed-effects model demonstrated the best overall fit and was selected as the final model.

District level random intercepts were included to account for unobserved heterogeneity between districts. Sensitivity analyses were additionally conducted because hospital distance and road distance were highly correlated; so the final model excluded hospital distance based on improved AIC and model stability.

Model diagnostics included;

1. assessment of multicollinearity using generalized variance inflation factors (GVIFs),
2. evaluation of residual patterns,
3. and comparison of observed versus fitted dengue counts.

Diagnostic summaries indicated the model captured broad temporal and spatial dengue patterns reasonably well, although fitted totals exceeded observed totals. It suggests imperfect calibration. As a result, the final model outputs were interpreted primarily as adjusted association rather than precise forecasting predictions.

# **Results (Dengue status and Distribution)**


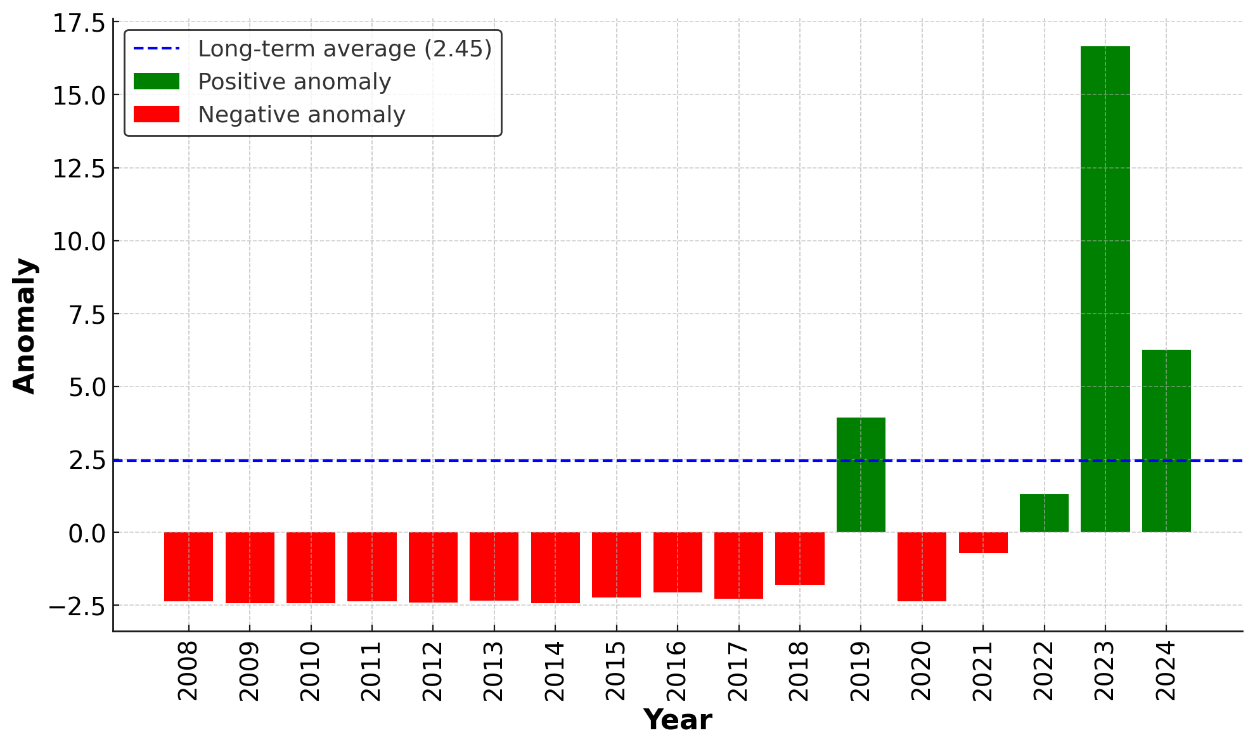


**Figure S12: Dengue cases per 10,000 population anomaly from 2008-2023 (source: IEDCR, 2019; DGHS,,2023)**


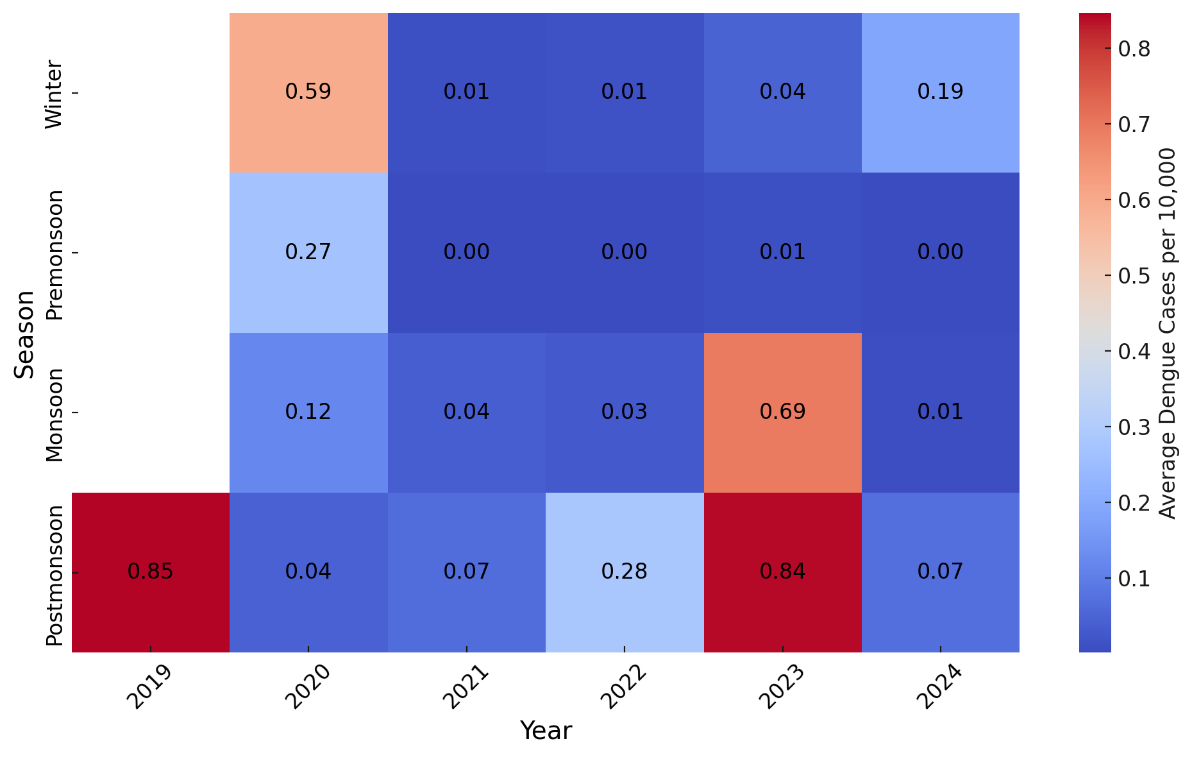


**Figure S13: Dengue case heatmap for seasons and years shows early emergence of dengue peak in 2023 during monsoon**


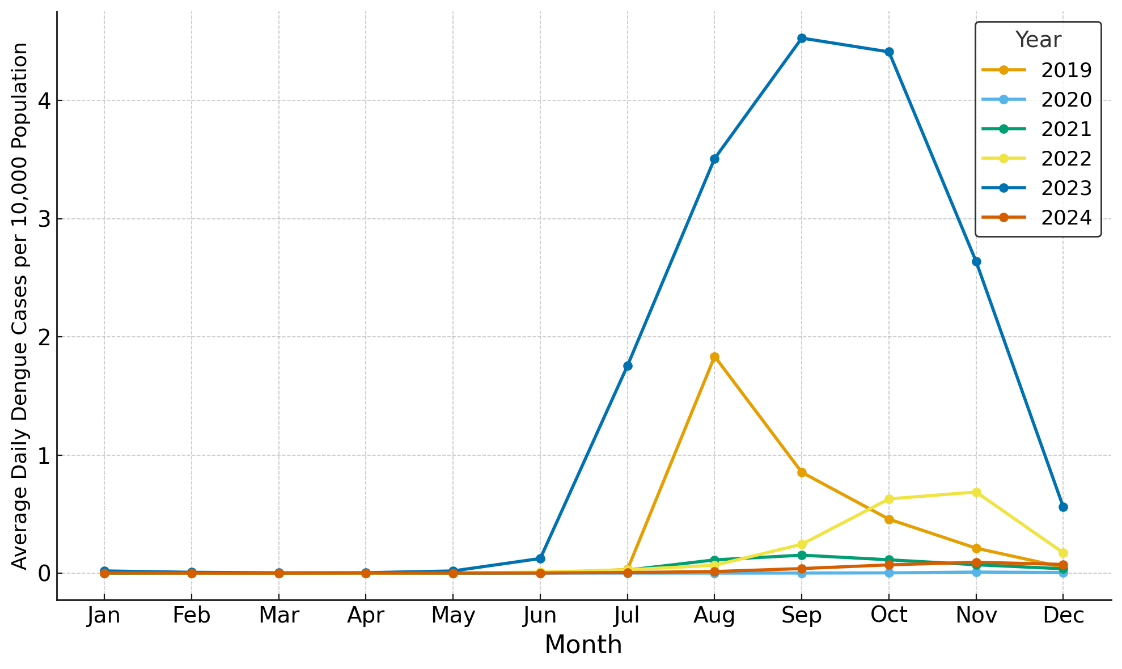


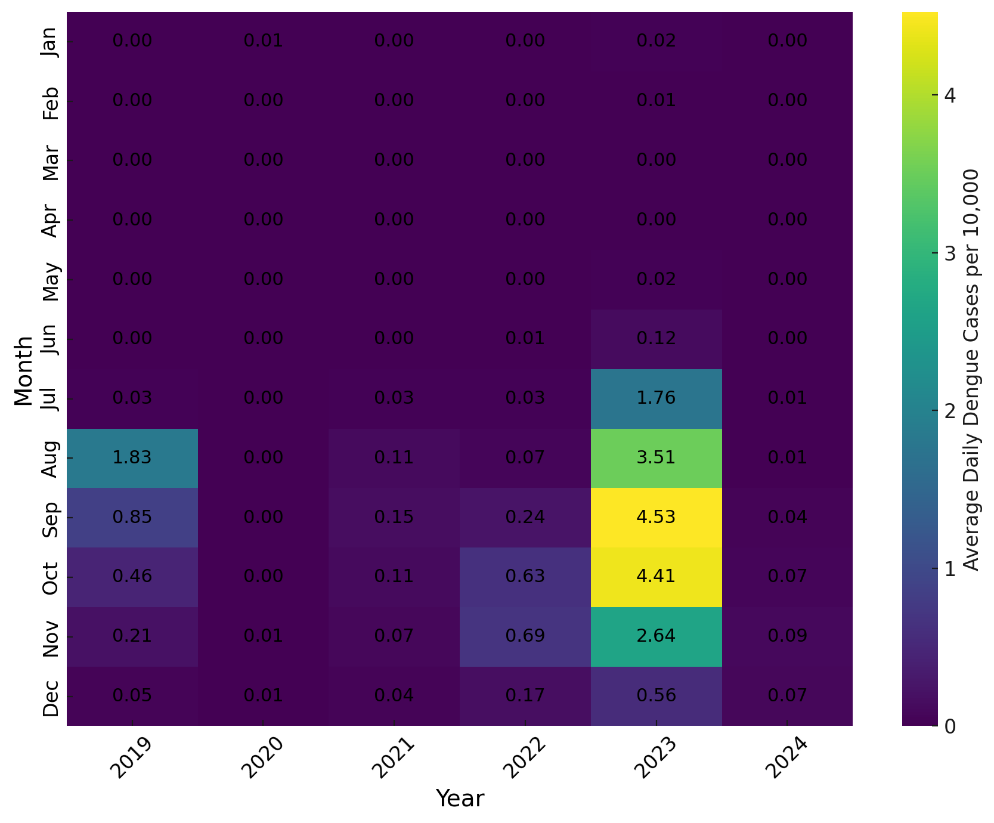


**Figure S15: Dengue case heatmap with month and year showing highest dengue month to be July, August, September, November and December**

**Figure S14: Monthly dengue trend and peak shift in 2023 which started early in June**

**
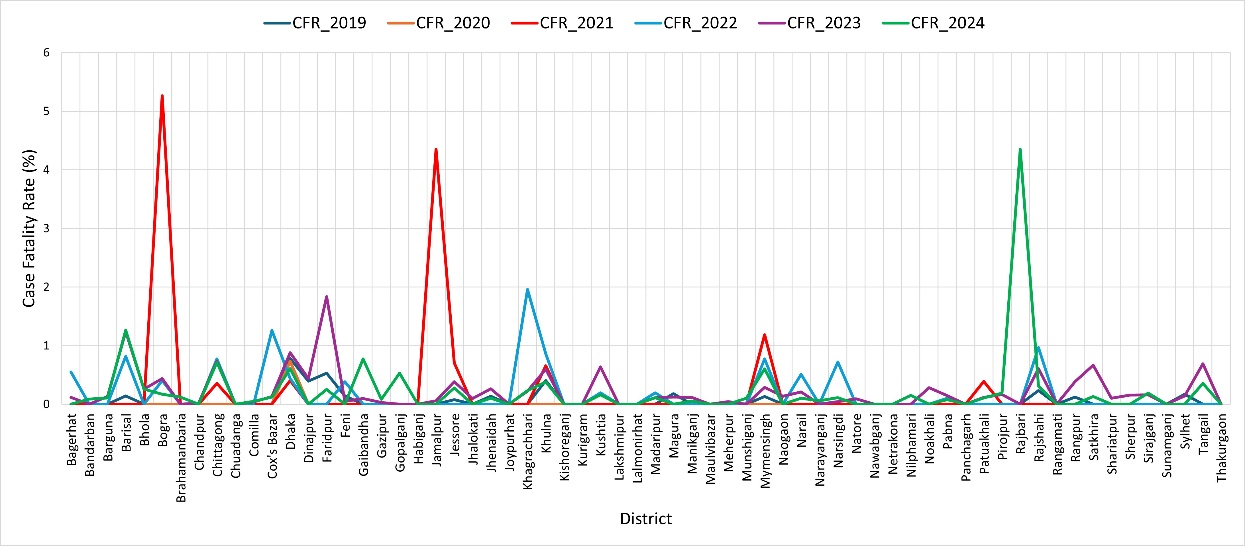
**

**Figure S16: District wise case fatality rate (CFR) showing Barisal and Faridpur having the highest CFR in 2023**


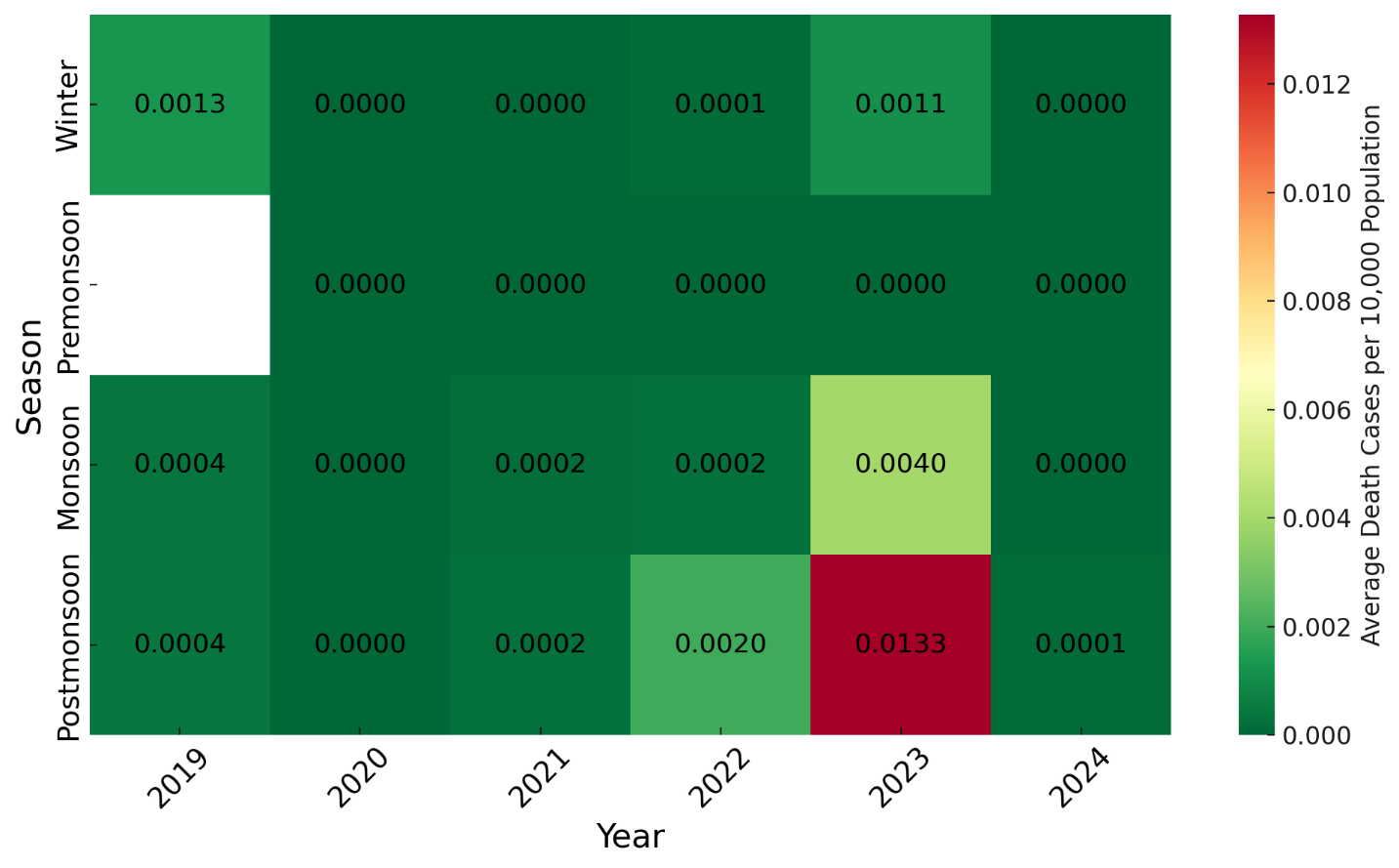


**Figure S17: Dengue death heatmap showing highest death rates during post-monsoon**

# **Results (Spatial autocorrelation and Hotspots)**

**
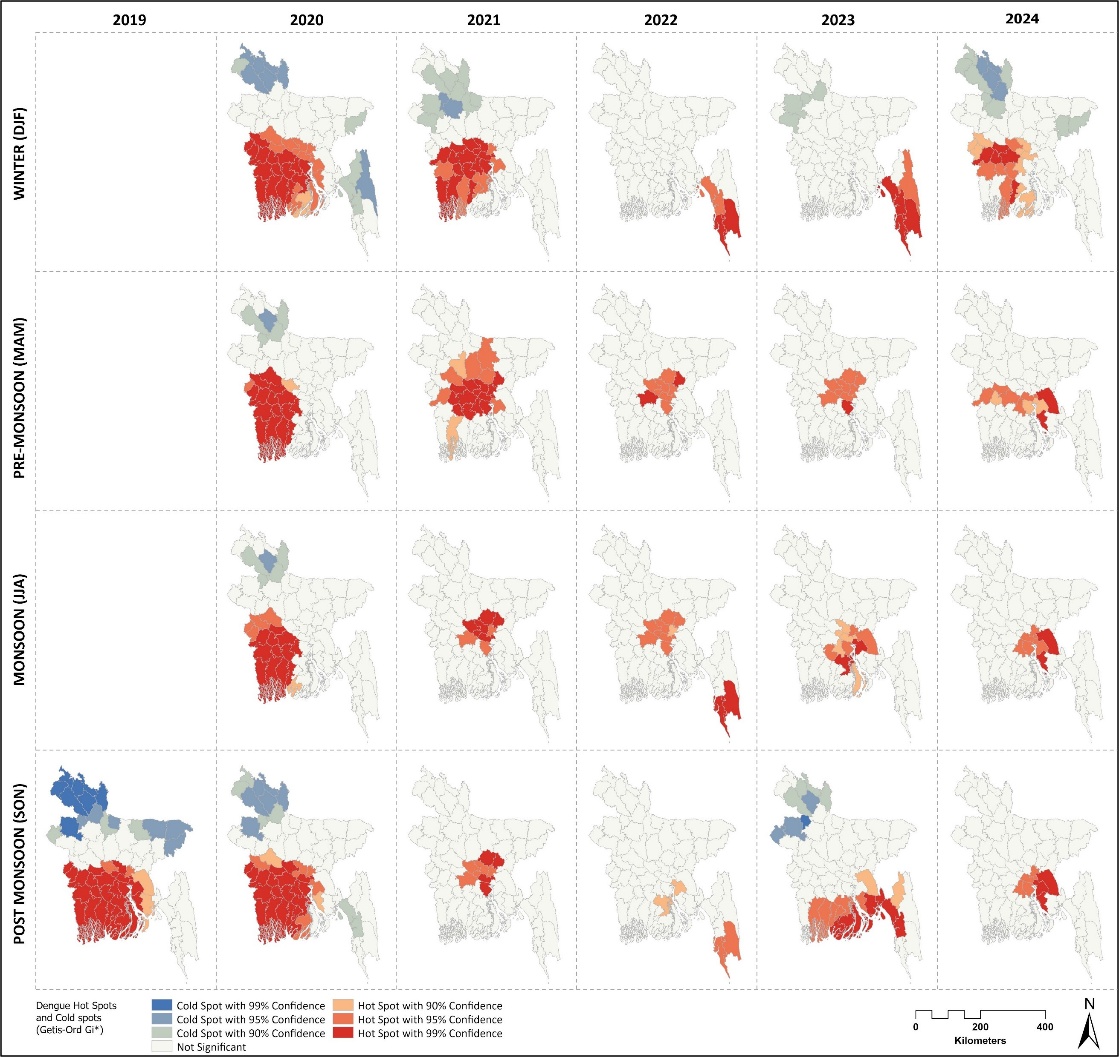
**

**Figure S18: Getis-ord G* dengue seasonal hotspot**

**
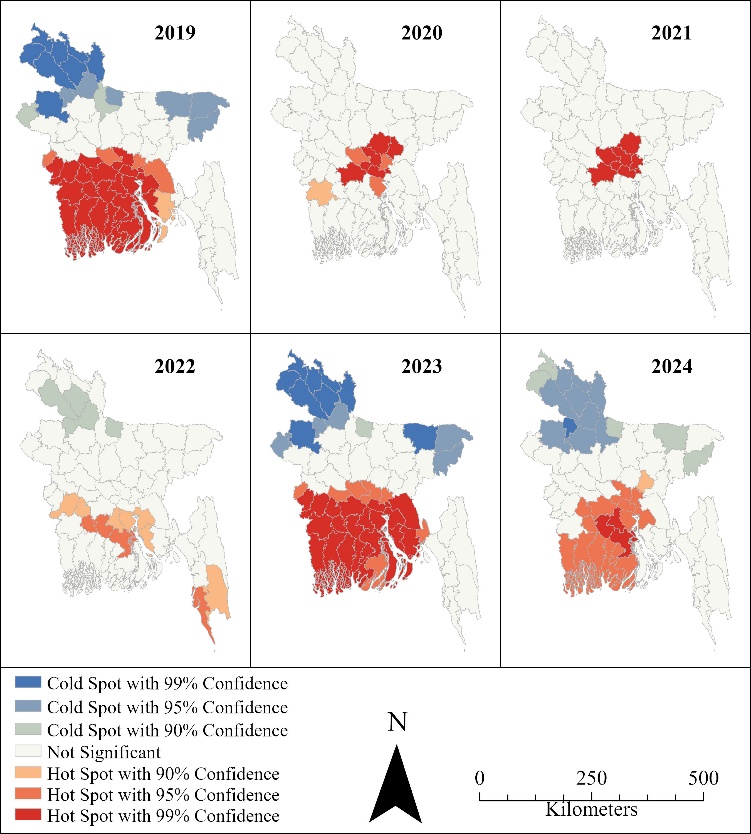
**

**Figure S19: Getis-ord Gi* annual dengue hotspot (2019-2024)**


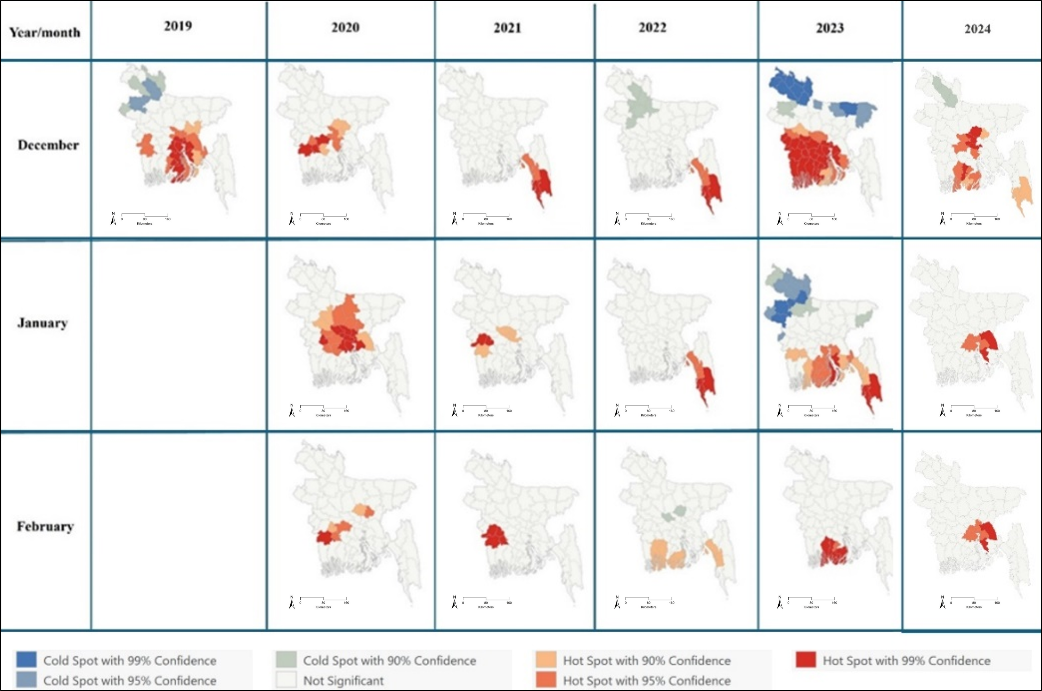


**Figure S20: Getis-Ord Gi* hotspot monthly dengue hotspot (winter)**


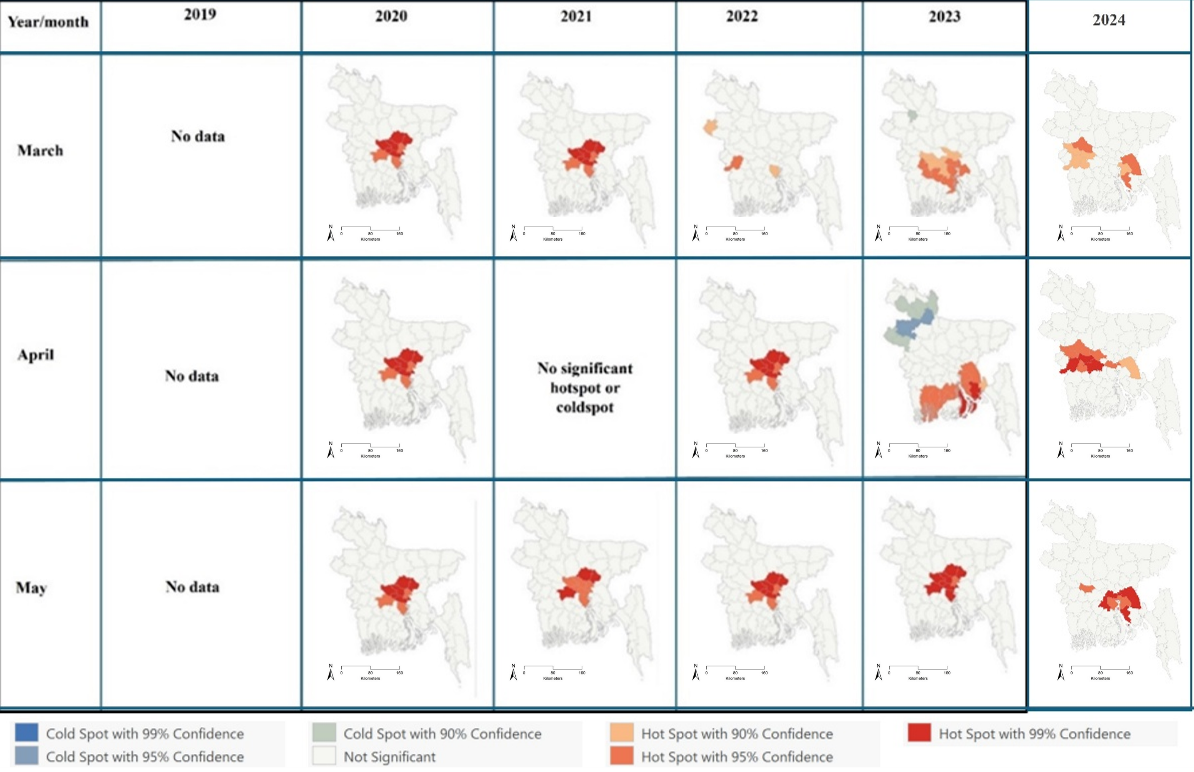


**Figure S21: Getis-Ord Gi* monthly dengue hotspot (pre-monsoon)**


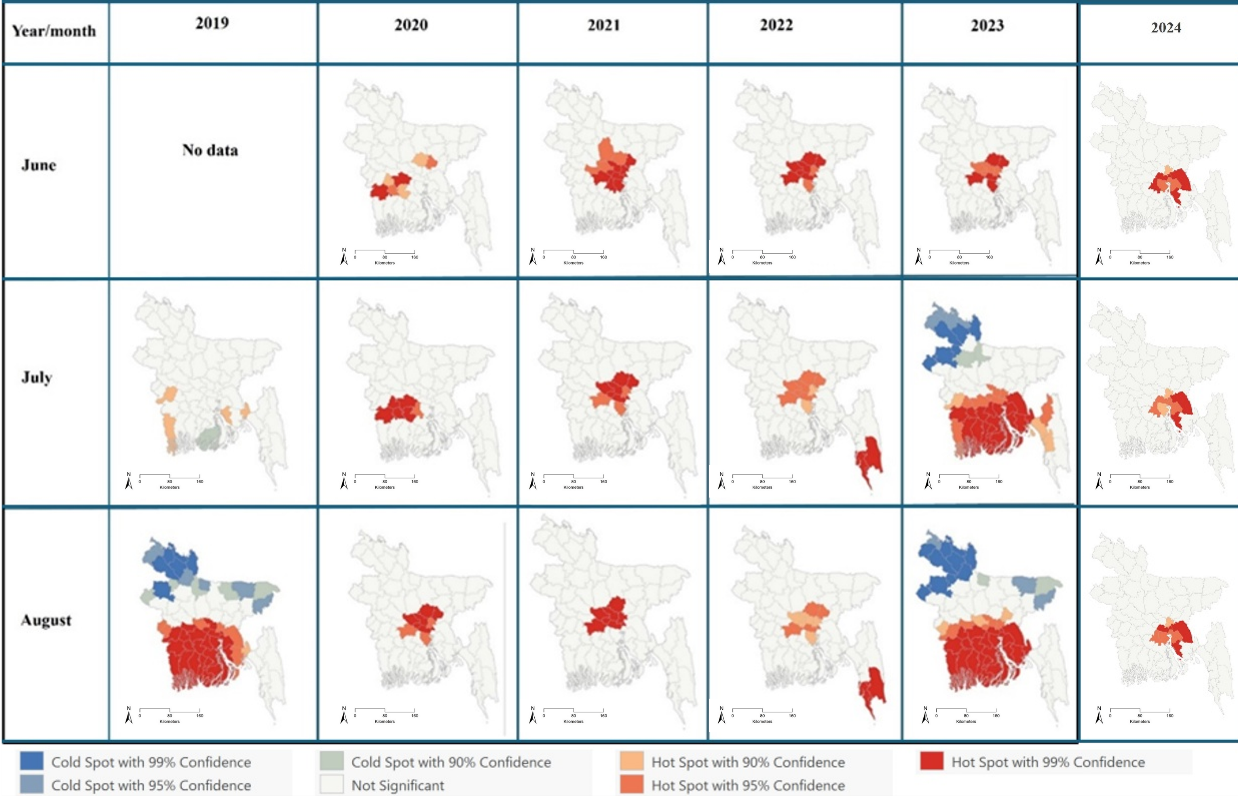


**Figure S22: Getis-Ord Gi* monthly dengue hotspot (monsoon)**


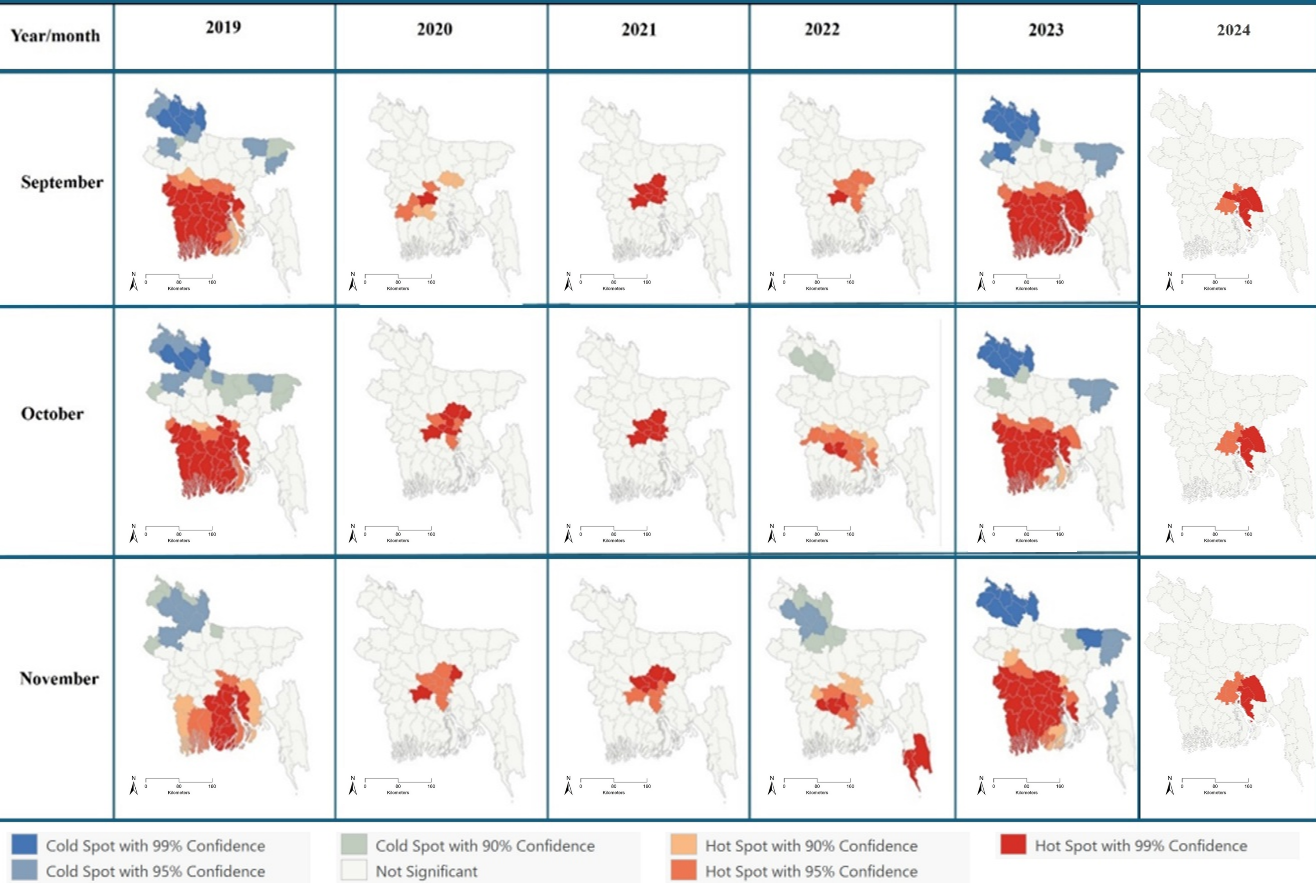


**Figure S23: Getis-Ord Gi* monthly dengue hotspot (post-monsoon)**


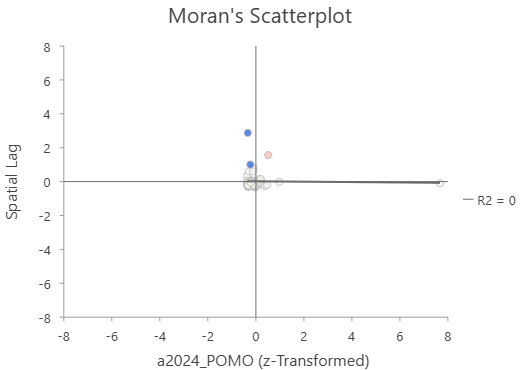

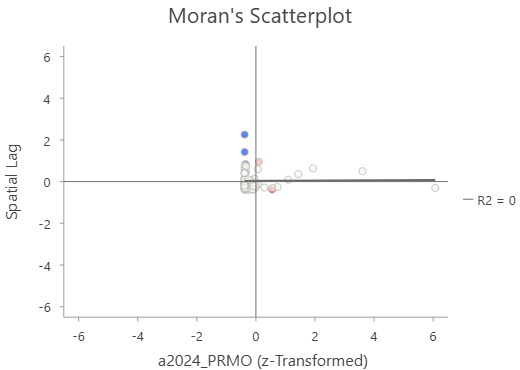


**Figure S 35: Seasonal Local Moran's I for 2021**

Figure A 3: Seasonal Local Moran's I for 2021

***Yearly Local Moran’s I***


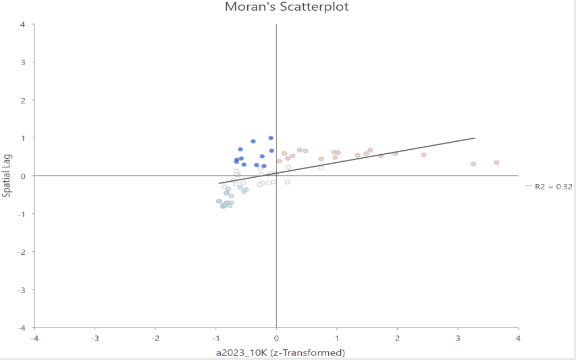

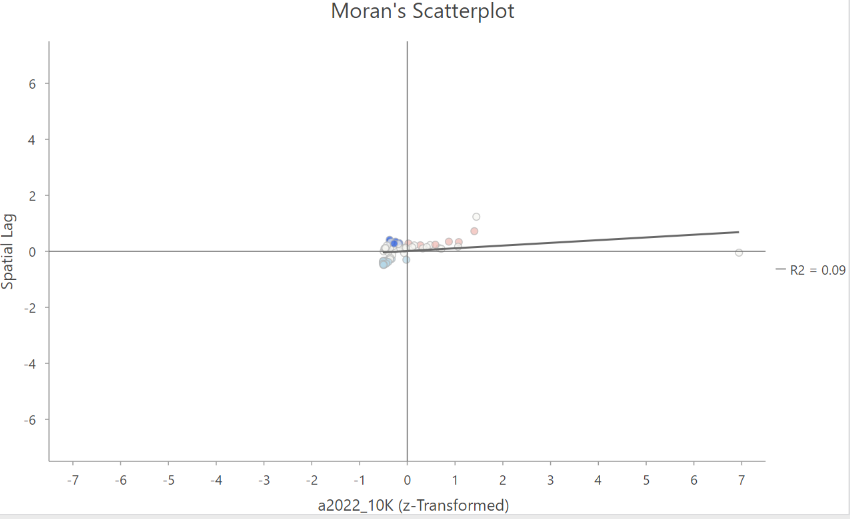

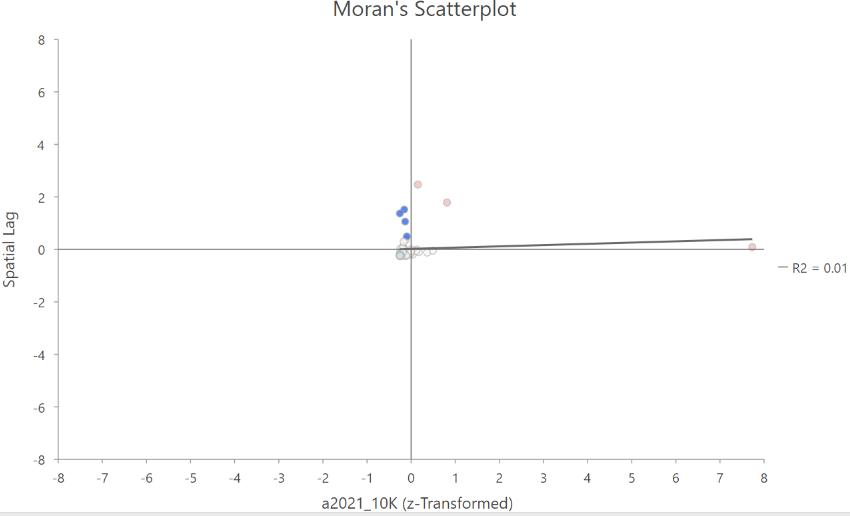

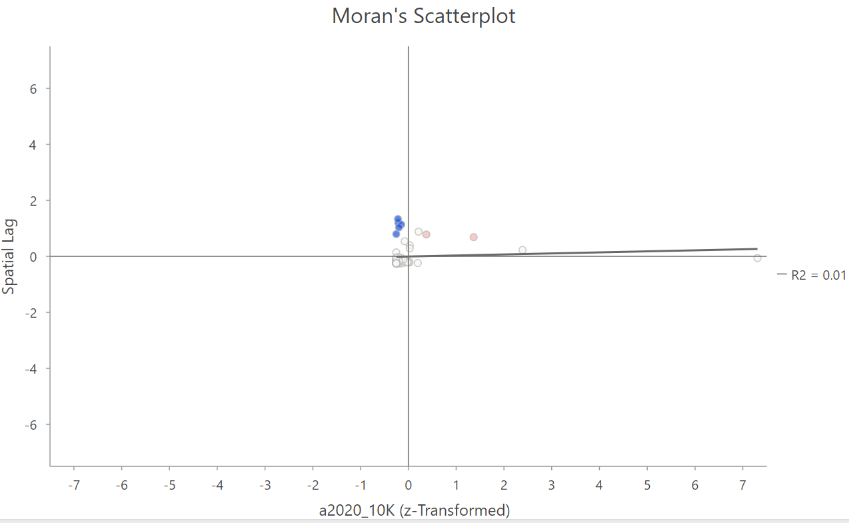

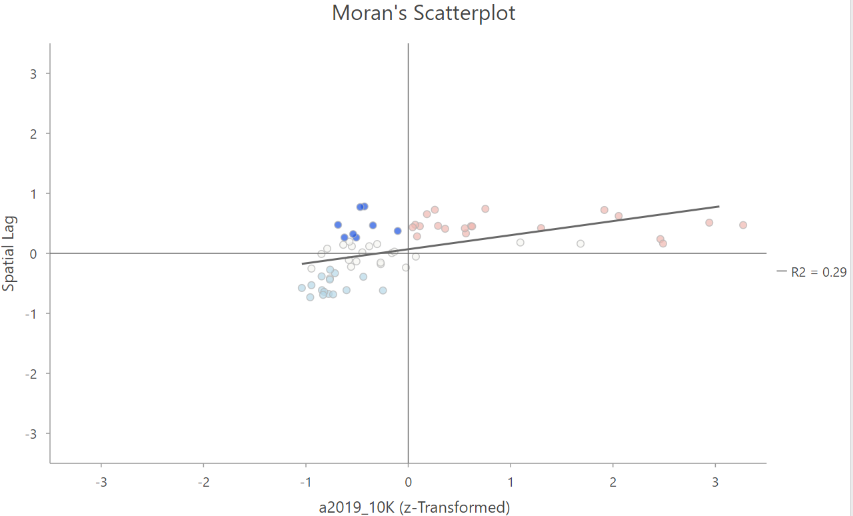


**2022**

**2022**

**2022**

**2022**

**2021**

**2021**

**2021**

**2021**

**2020**

**2020**

**2020**

**2020**

**2019**

**2019**

**2019**

**2019**

**2023**

**2023**

**2023**

**2023**


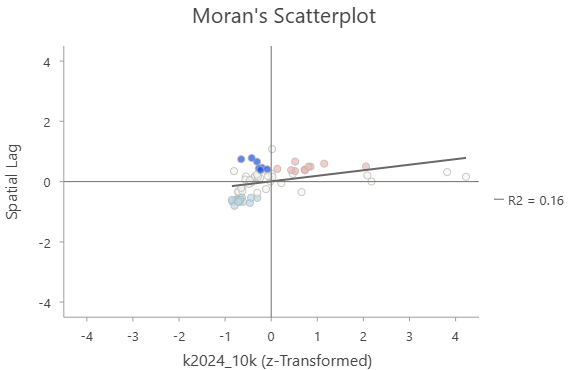


**2024**

**2023**

**2023**

**2023**

**Figure S24: Yearly Local Moran's I Scatterplot for dengue cases**

# **Results (Dengue risk indicators, overall risk, correlation, Validation)**

**Figure S25: Districts with high and low dengue risk (area basis) during December-February 2023**


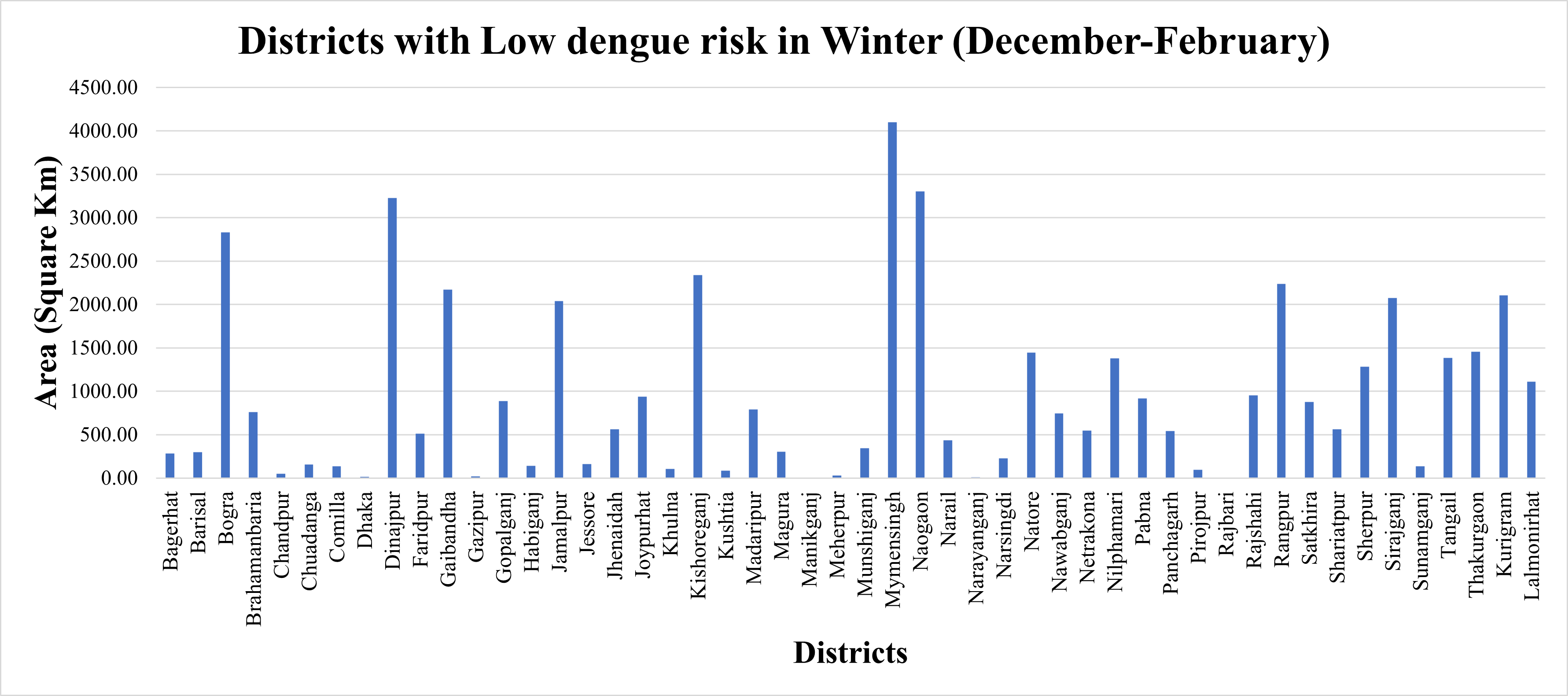

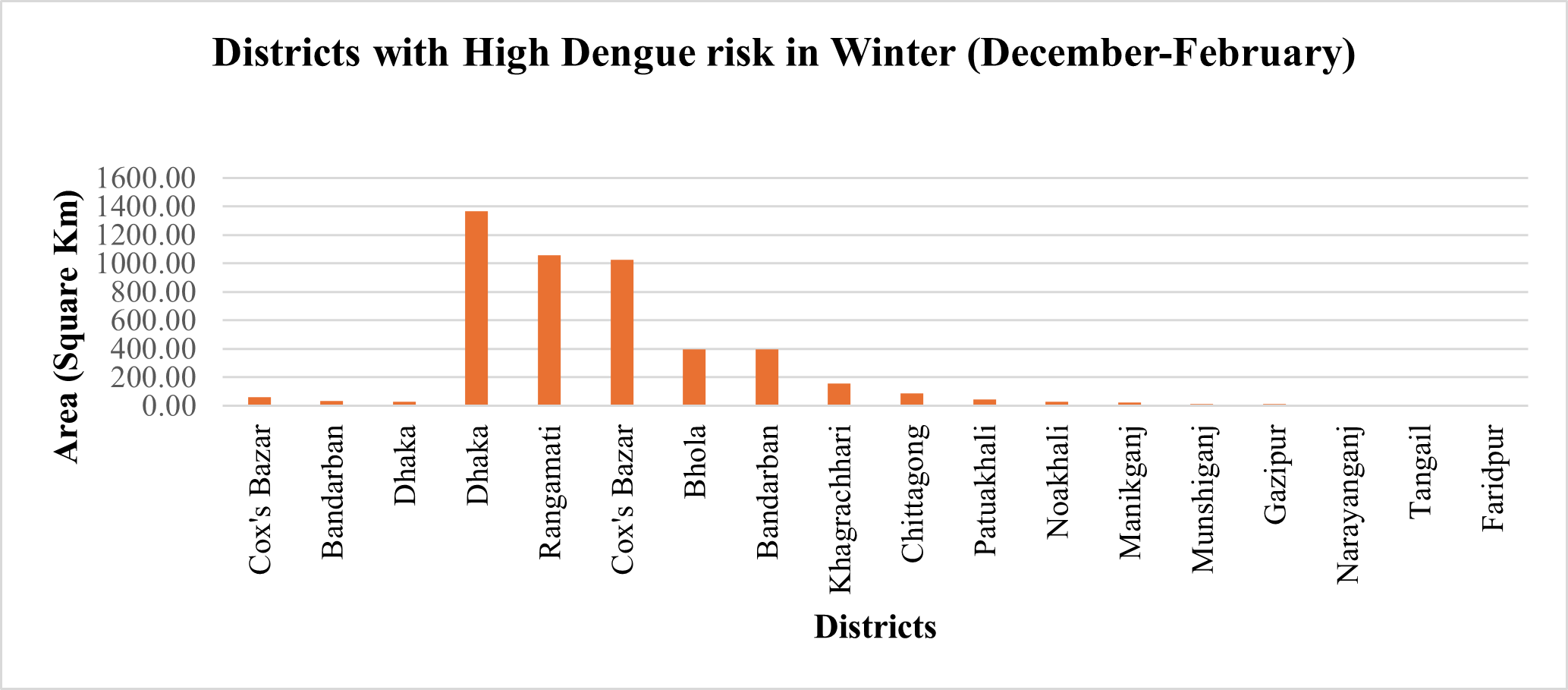


**
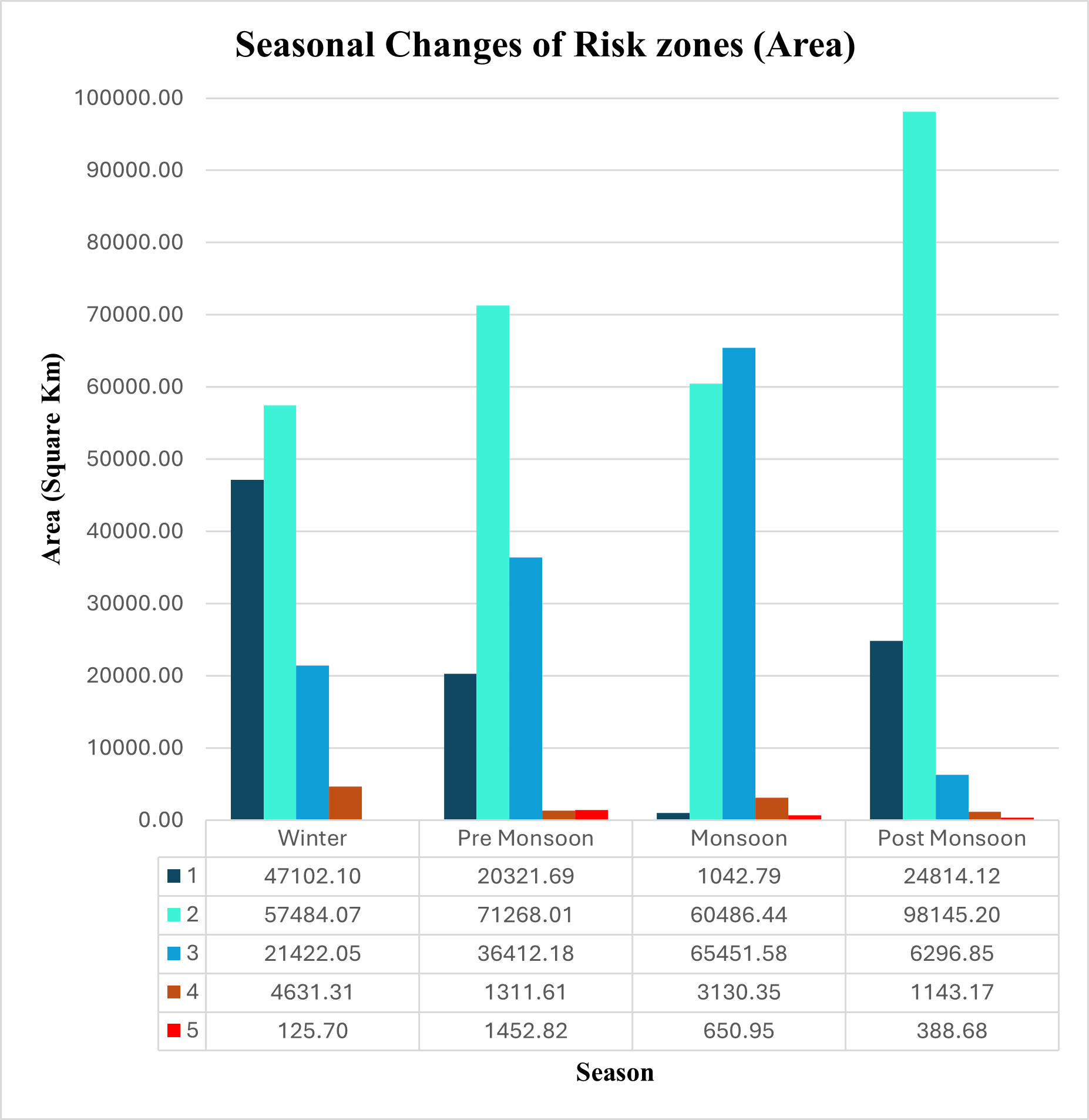
**

**Figure S26: Seasonal changes of risk area in Bangladesh during 2023 (according to risk score)**

**Figure S27: Districts with high and low dengue risk (area basis) during March-May 2023**


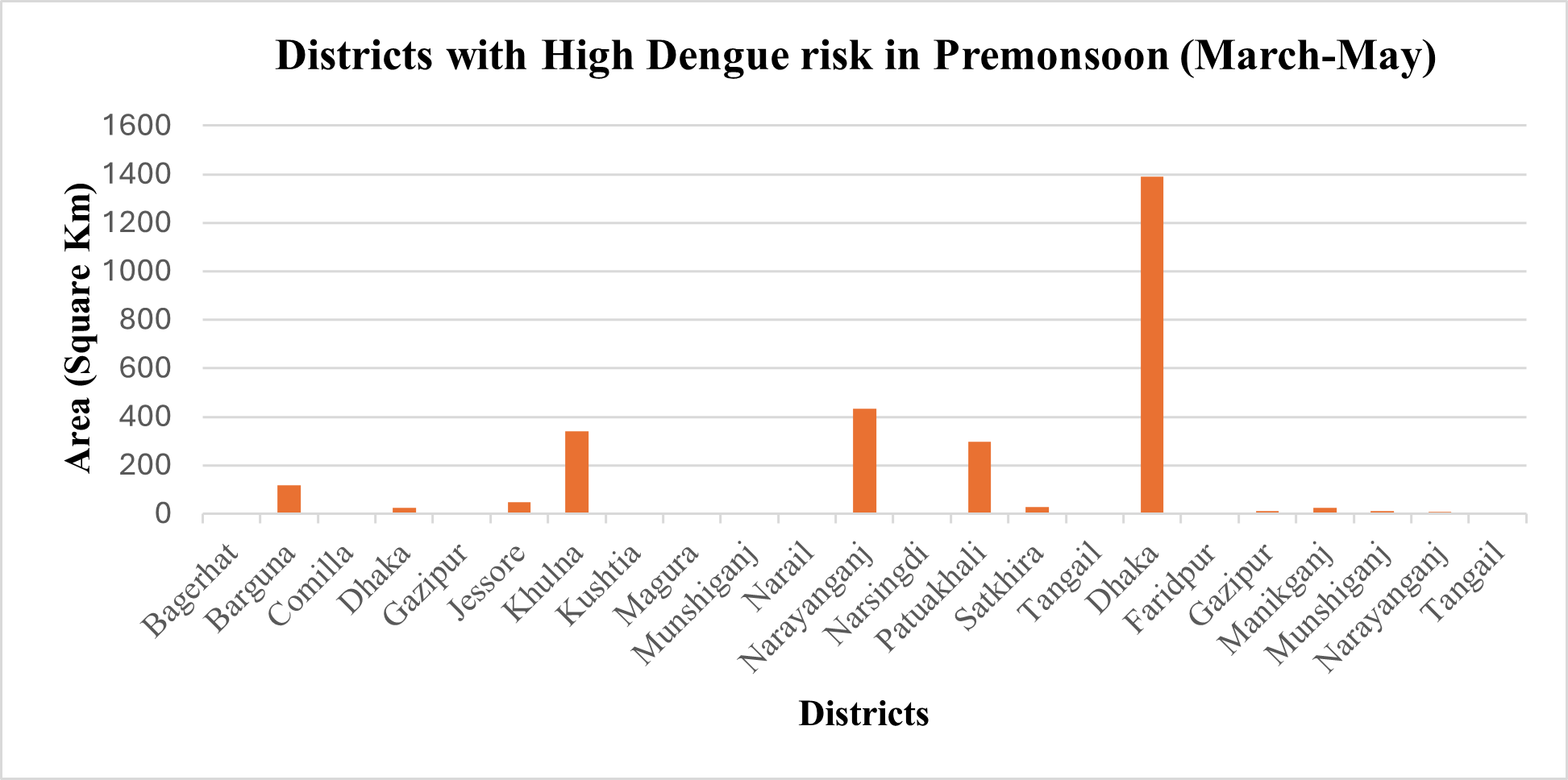

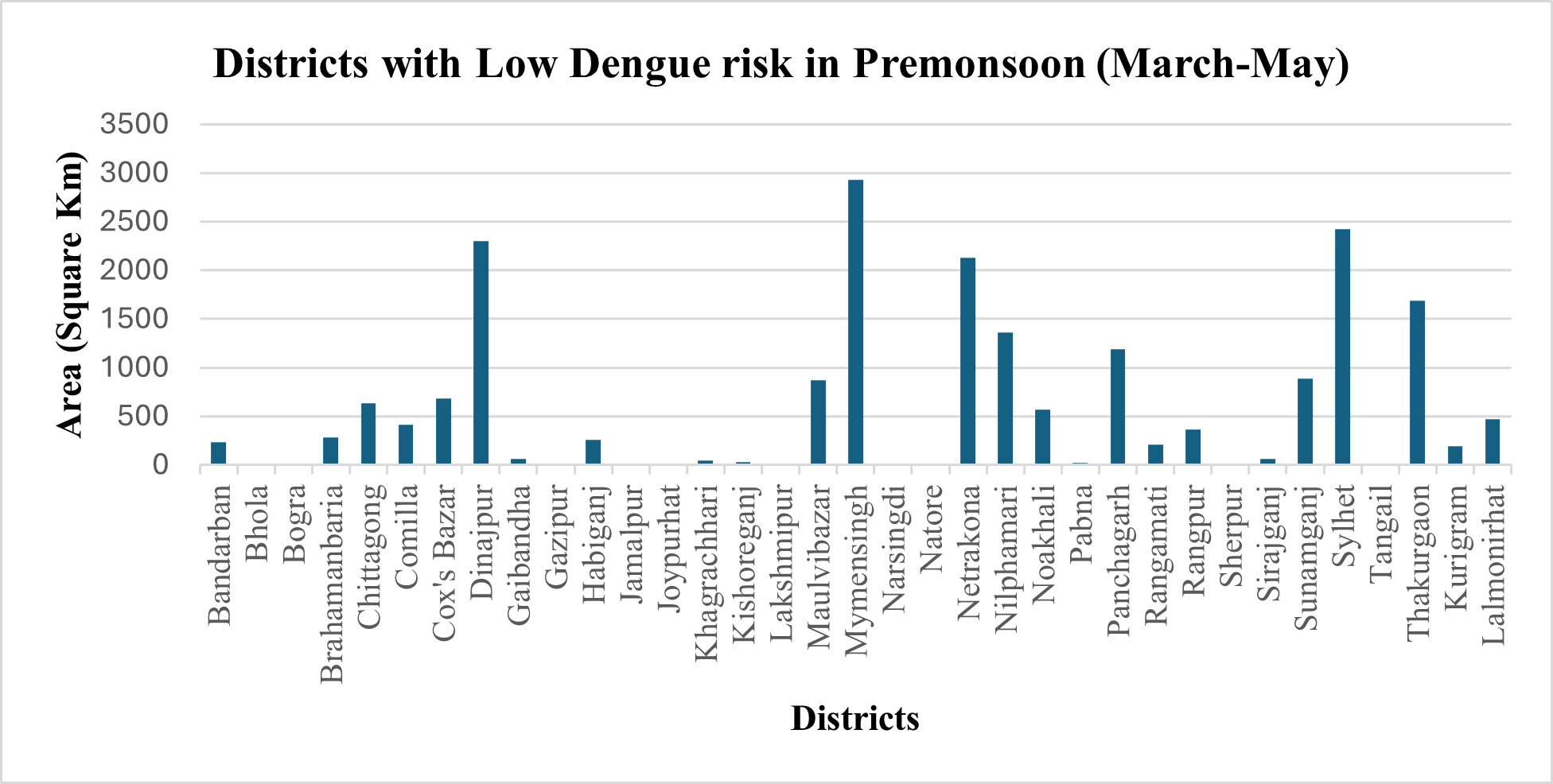


**Figure S28: Districts with high and low dengue risk (area basis) during June-August 2023**


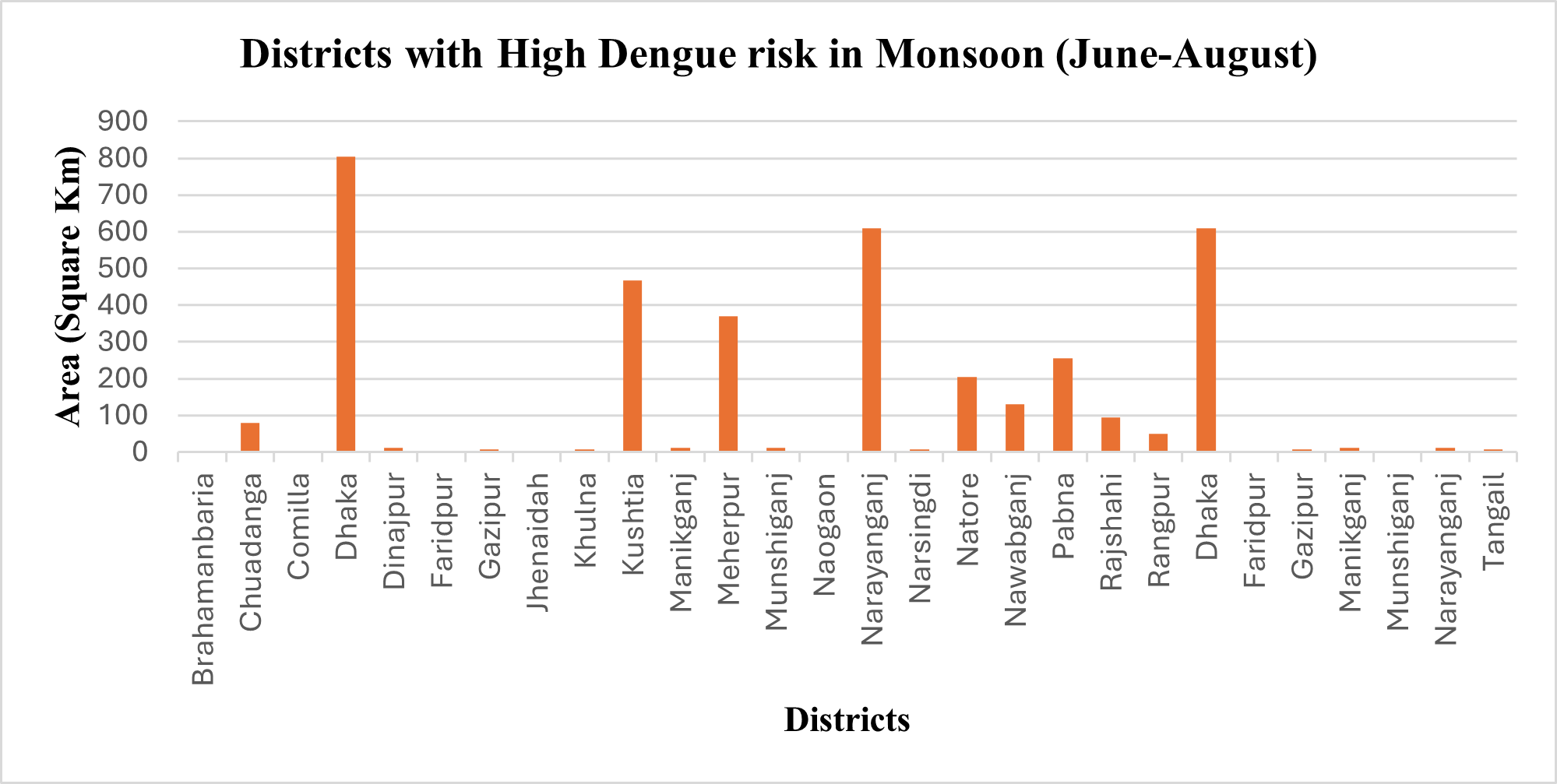

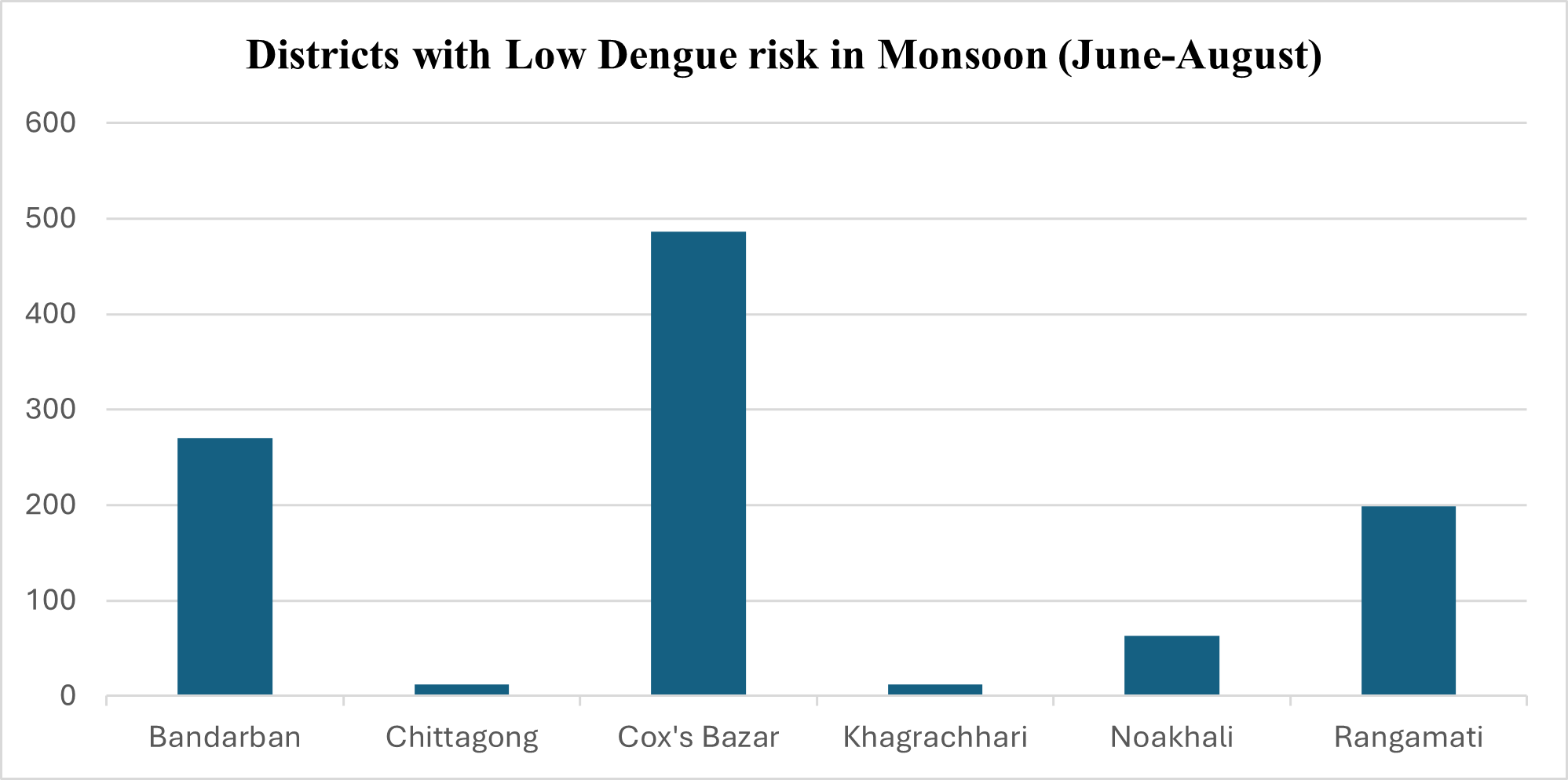


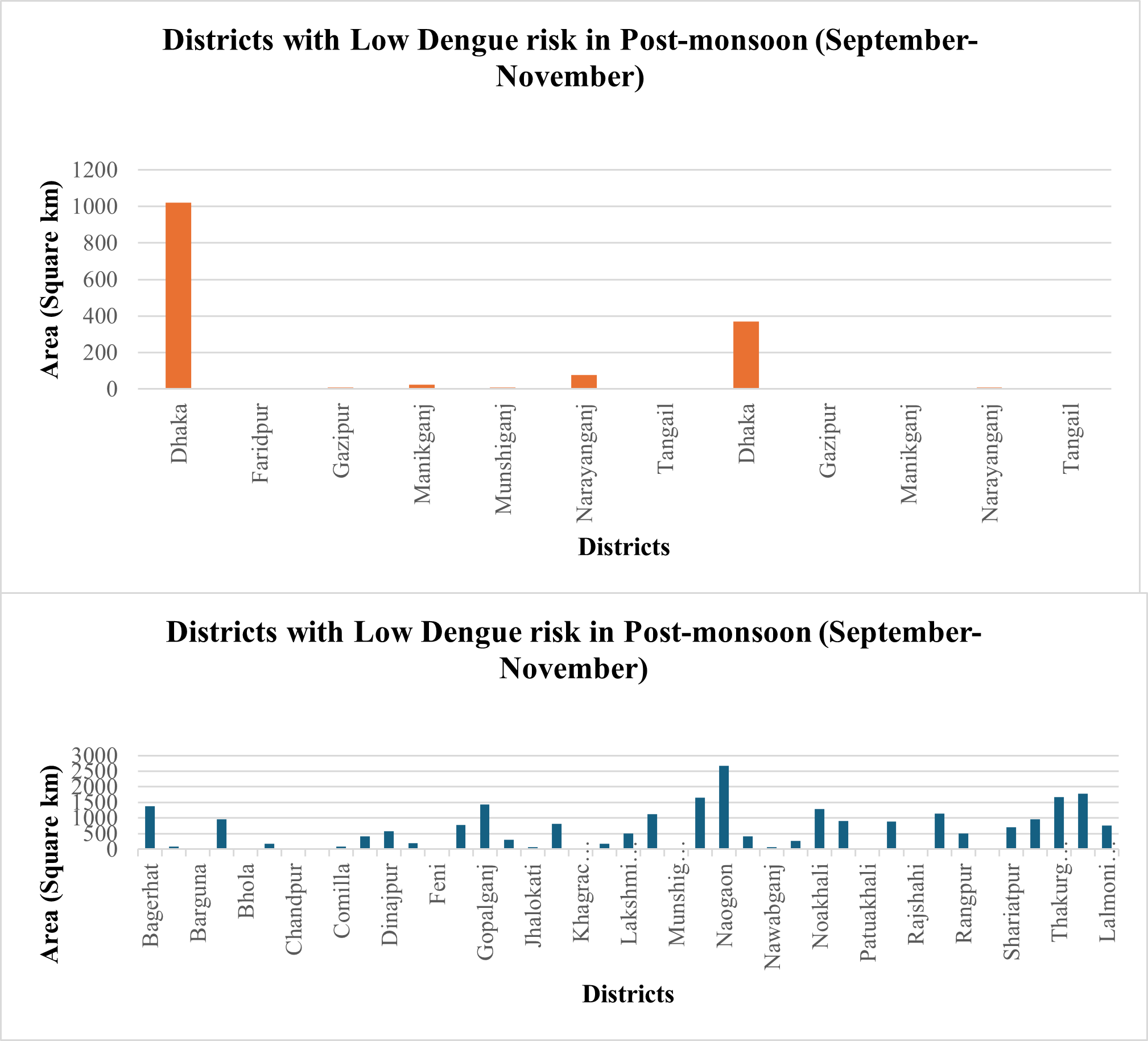


**Figure S29: Districts with high and low dengue risk (area basis) during September-November 2023**

**
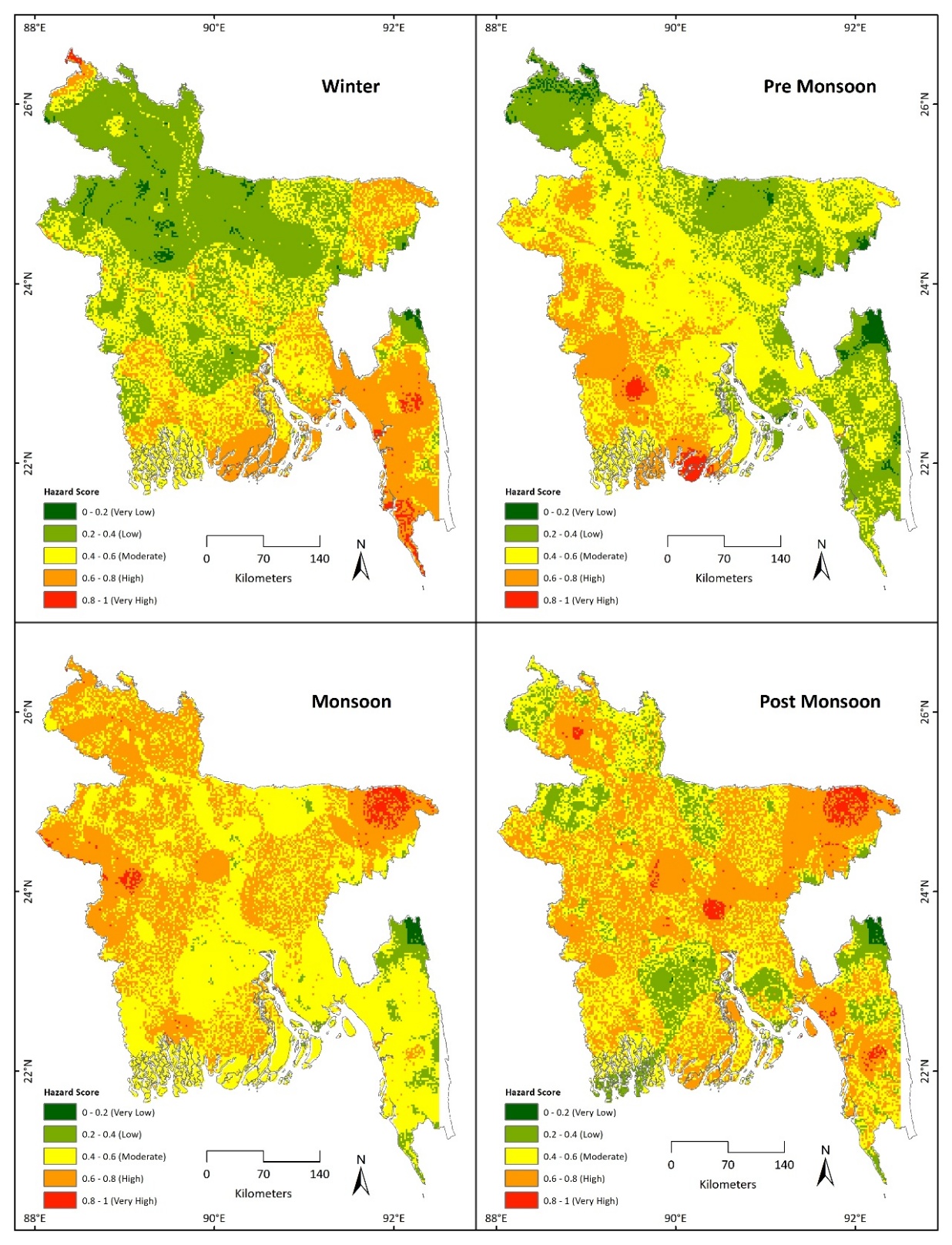
Unweighted Dengue Hazard, Vulnerability and Risk**

**Figure S30: Dengue seasonal hazard map with unweighted model (factors contributing equally) shows the less localized pattern of dengue hazard**

**
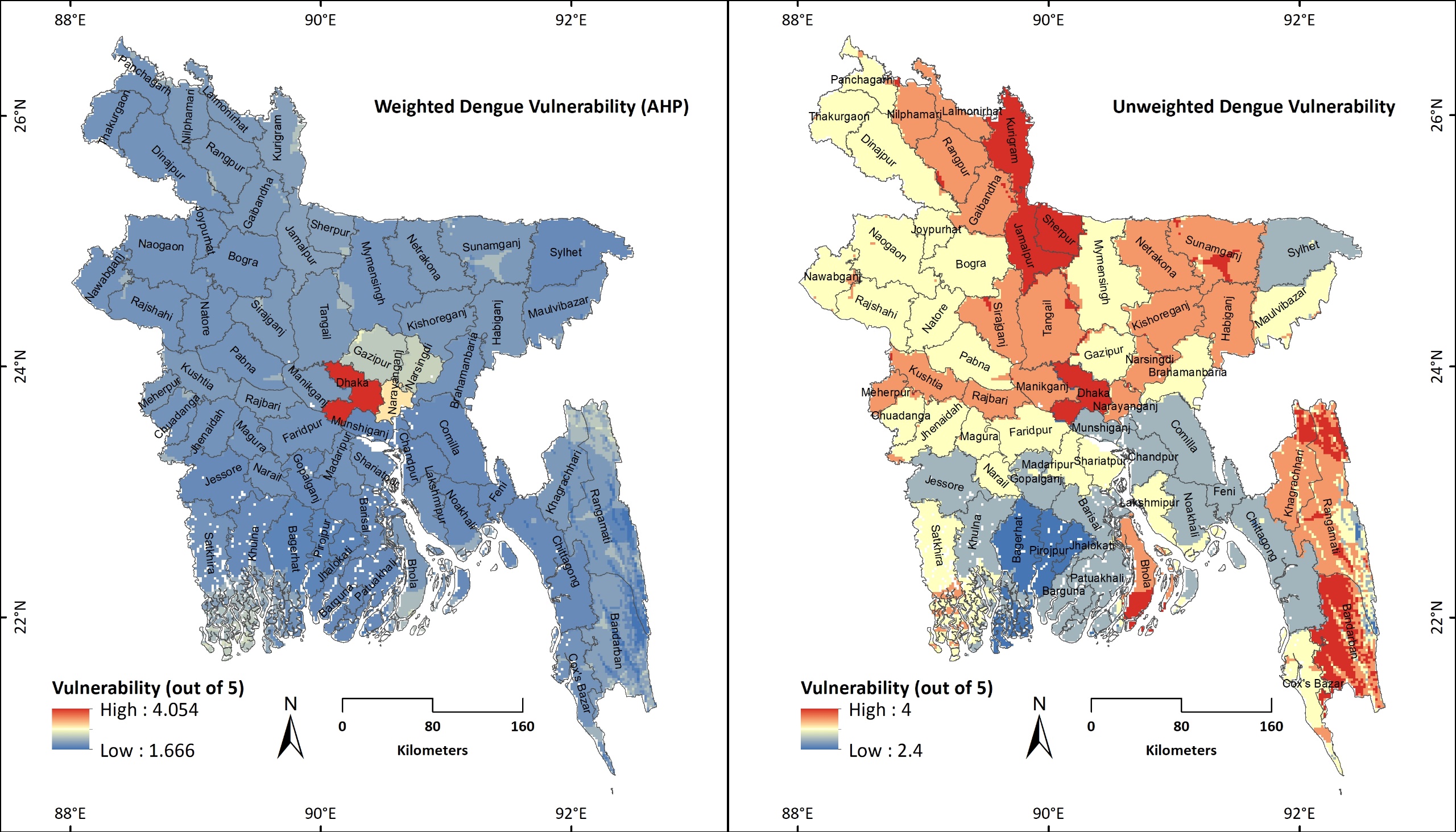
**

**Figure S31: Dengue vulnerability map with unweighted model shows higher vulnerability than the AHP weighted method**

***
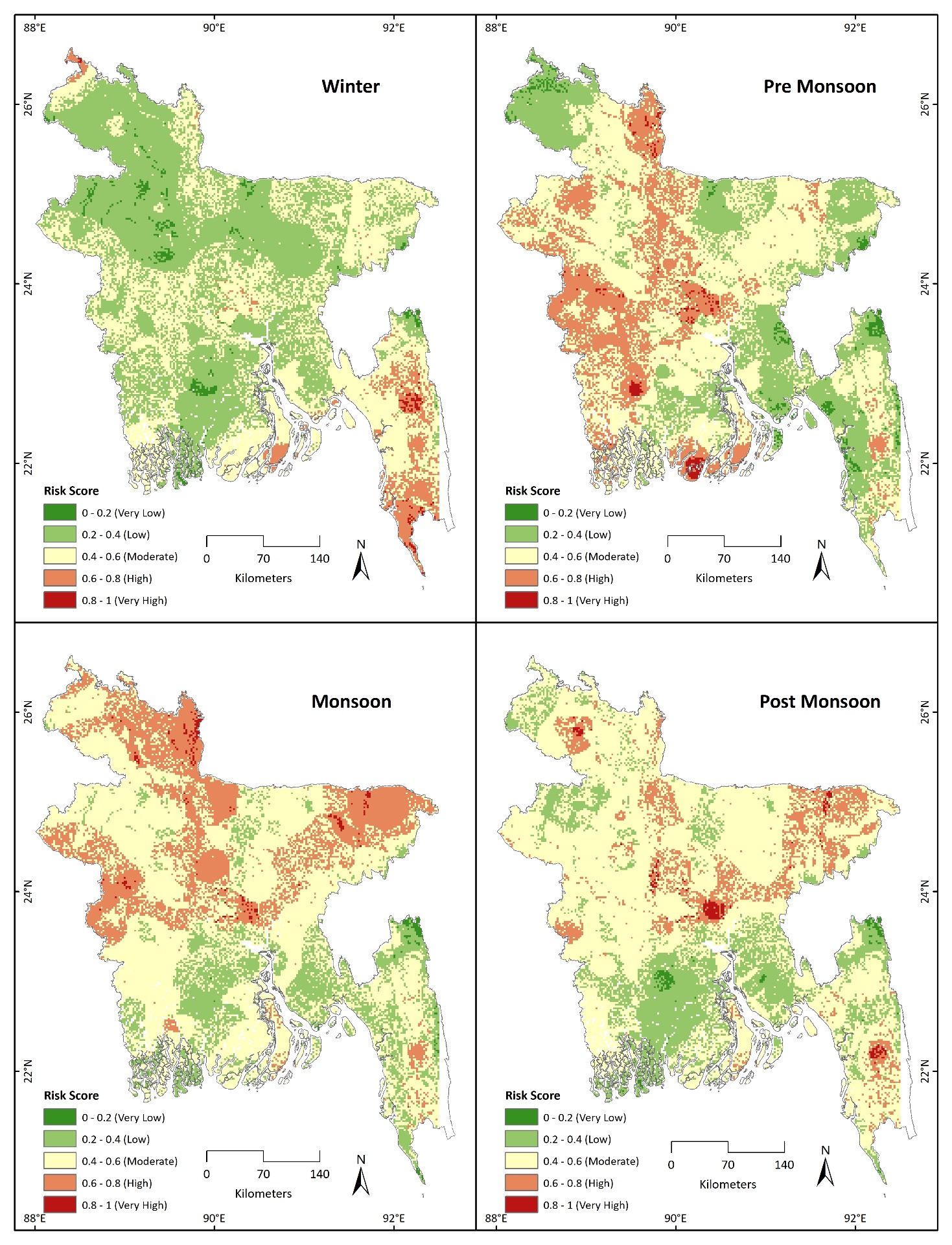
***

**Figure S32: Dengue risk with unweighted model shows more uniform dengue risk throughout the country**


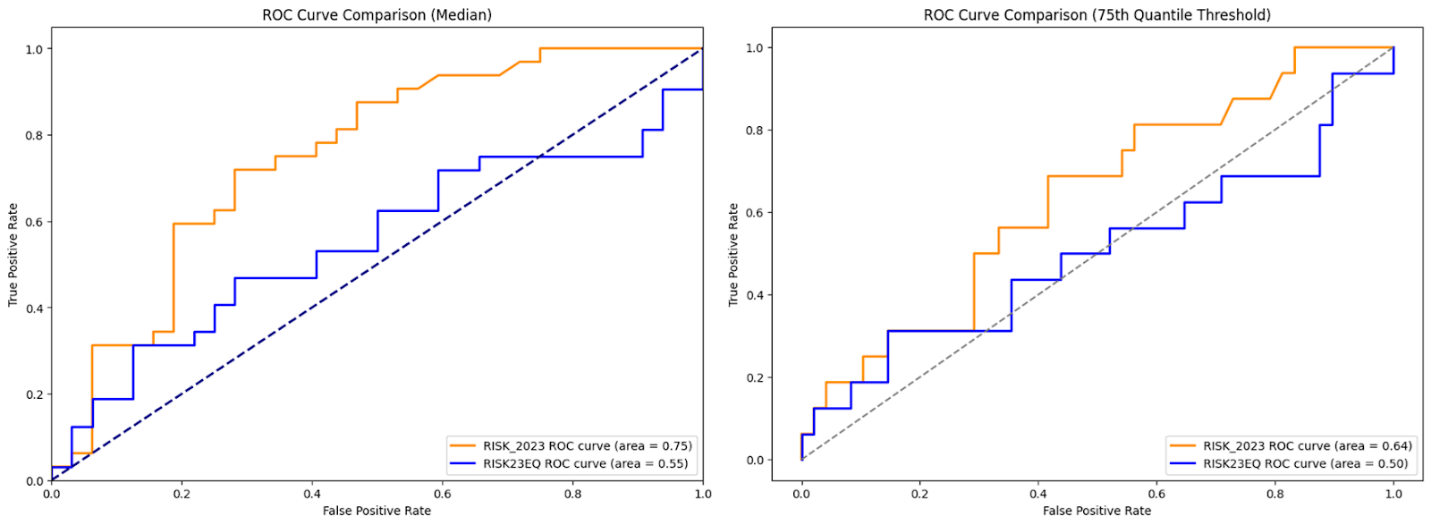
***Validation***

**Figure S33: Validation and comparison of weighted and unweighted models using a median and 75^th^ threshold**


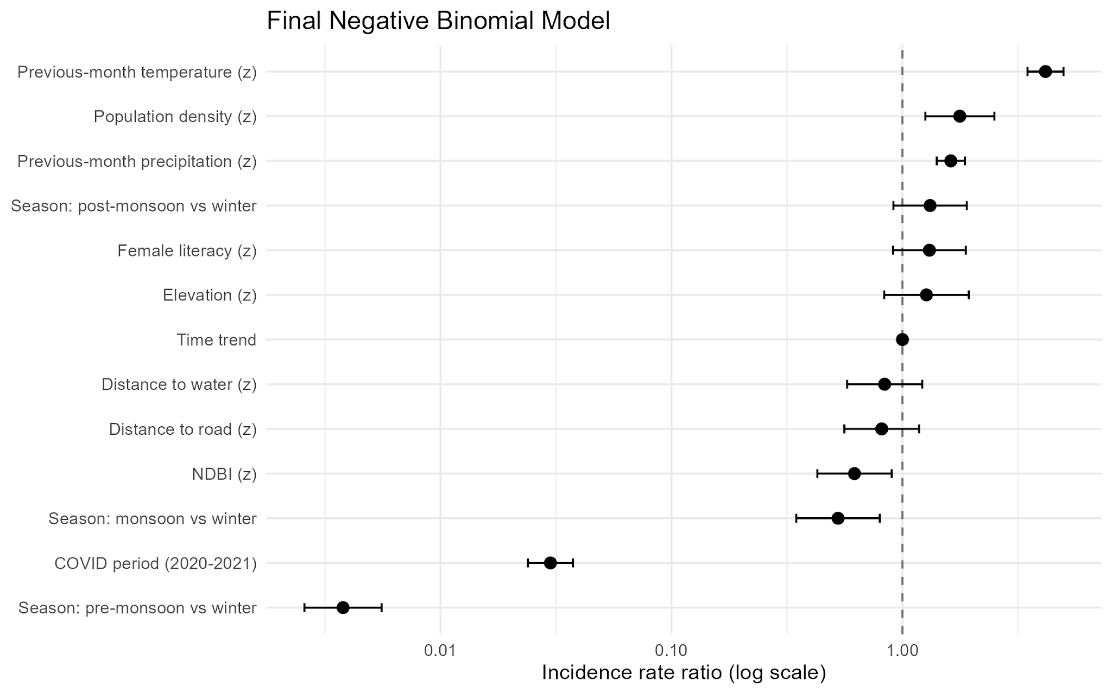
***Correlation Results***

**Figure S34: Incidence Rate Ratio (IRR) for dengue incidence and covariates in negative binomial mixed-effect model**


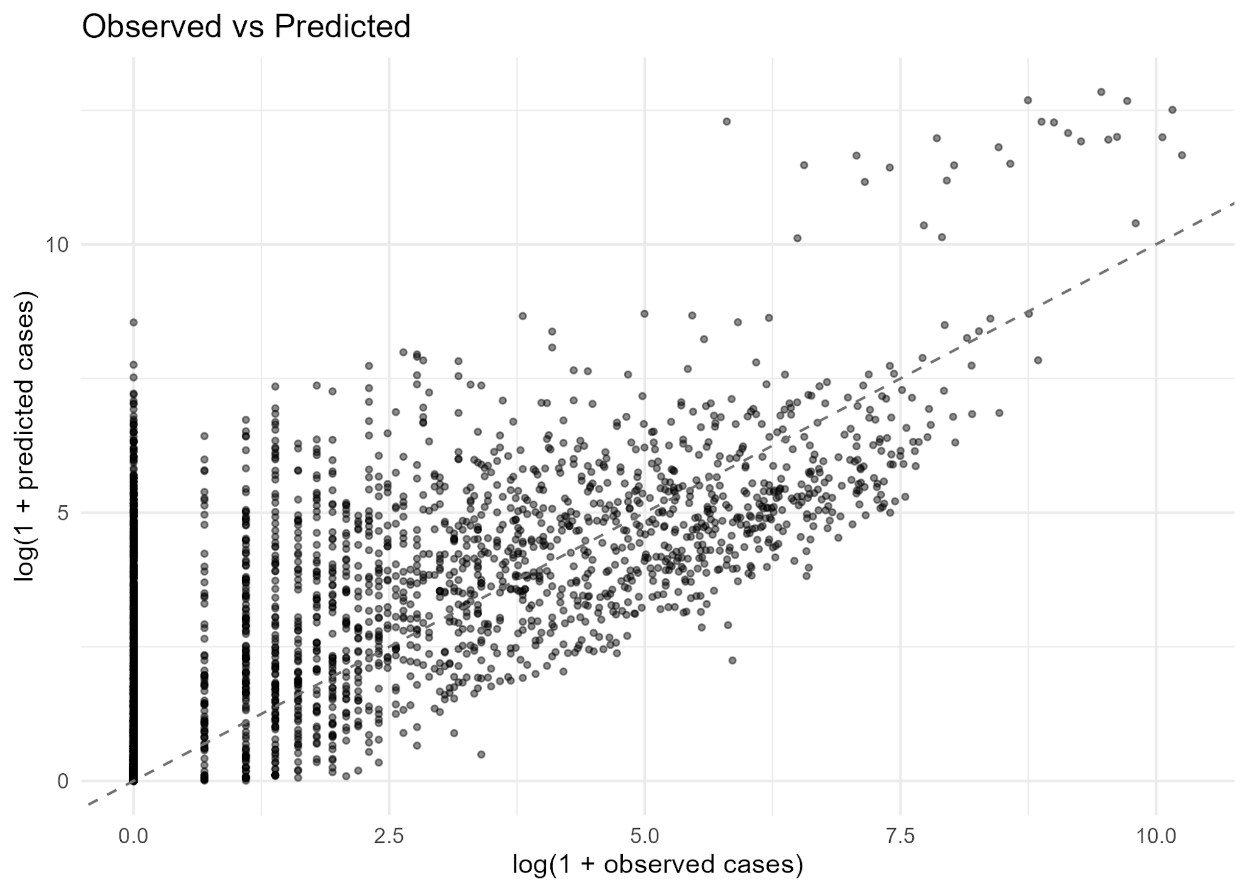


**Figure S35: Observed vs predicted dengue case counts for the negative binomial mixed effect model**

**Table S1: Data and factors of dengue risk with their relationship with dengue overall risk**

| **Data type** | **Data source** | **Pre-processing** | **Relationship with Dengue Risk** | **Risk Element** |
| --- | --- | --- | --- | --- |
| Dengue Daily Incidence, death and admitted patient (2019-2023) | Directorate Geneal Health Services (DGHS) | Tabulated, cleaned to district level and translated | Validation layer |  |
| Maximum monthly average temperature (station) | Bangladesh Meteorological Department (95 station data) | Tabulated 12 months of Maximum daily temperature ( December, 2022 to December, 2023 and monthly maximum average temperatures were calculated. at the point data was rasterized at 30 meter spatial resolution. | Positive linear (Lai et al., 2018) | Hazard |
| Precipitation | Maximum rainfall from Bangladesh Meteorological department (BMD) | 12 months of precipitation (mm) data was tabulated and rasterized from December, 2023 to December, 2024 at 30 meter spatial resolution. | Positive linear (Malik et al.,2017) | Hazard |

***Table S1 Continued***

| **Data type** | **Data source** | **Pre-processing** | **Relationship with dengue risk** | **Risk element** |
| --- | --- | --- | --- | --- |
| Population Density | Bangladesh Bureau of Statistics, 2022 census | A raster layer for population 2022 was created using conversion tool in ArcGIS Pro at 30 meter spatial resolution | Positive linear (Yue et al.,2018) | Vulnerability |
| Normalized Difference Built up Index (NDBI) | Landsat 8 Collection 2 Tier 1 TOA (Top of Atmosphere) reflectance  Spatial resolution: 30 meter  Temporal resolution: 16 days | Using Shortwave infrared band and Near infrared band, the normalized built up index was calculated using the following formula :  NDBI=  SWIR+NIR  SWIR−NIR | Positive linear (Romero et al.,2019) | Hazard |
| Elevation | NASA SRTM  (spatial resolution: 30 meter) | Symbolized in ArcPro | Negative linear, Istiqamah et al.,2020 | Vulnerability |
| Distance from hospitals | DGHS | The layer is projected and Euclidean distance to hospitals network is considered at 30 meter spatial resolution | Negative linear | Vulnerability |

***Table S1 Continued***

| **Data type** | **Data source** | **Pre-processing** | **Relationship with dengue risk** | **Risk element** |
| --- | --- | --- | --- | --- |
| Open waterbody | Bangladesh Bureau of Statistics | The layer is projected and Euclidean distance to open waterbody is considered at 30 meter spatial resolution | Negative linear (Russel et al.,1993) | Hazard |
| Female literacy rate | BBS Census | Rasterized using ArcGIS pro at 30 meter spatial resolution | Negative linear  (Zafar et al., 2021) | Vulnerability |

***Table S2: Saaty's pairwise comparison matrix (Saaty, 1990; Ali & Ahmed, 2018)***

| **Verbal judgement** | **Numeric Value** |
| --- | --- |
| Extremely important | 9, 8 |
| Very strongly more important | 7, 6 |
| Strongly more important | 5, 4 |
| Moderately more important | 3, 2 |
| Equally important | 1 |

***Table S3: Random Index Table, n denotes number of components (Saaty,1980; Ali & Ahmed, 2018)***

| n | 1 | 2 | 3 | 4 | 5 | 6 | 7 | 8 | 9 | 10 |
| --- | --- | --- | --- | --- | --- | --- | --- | --- | --- | --- |
| RI | 0.00 | 0.00 | 0.58 | 0.90 | 1.12 | 1.24 | 1.32 | 1.41 | 1.45 | 1.49 |

***Table S4: Pairwise comparison of variables to calculate dengue hazard***

| **Components** | *Temperature* | *Precipitation* | *NDBI* | *Distance to waterbody* | *Weight* | *CR* |
| --- | --- | --- | --- | --- | --- | --- |
| *Temperature* | 1.00 | 2.00 | 5.00 | 6.00 | 0.505 | 0.05 |
| *Precipitation* | 0.50 | 1.00 | 4.00 | 5.00 | 0.322 |  |
| *NDBI* | 0.20 | 0.25 | 1.00 | 3.00 | 0.113 |  |
| *Distance to Waterbody* | 0.17 | 0.20 | 0.33 | 1.00 | 0.059 |  |

***Table S5: Pairwise comparison of variables used to calculate dengue vulnerability***

| **Components** | *Population Density* | *Distance to hospital* | *Elevation* | *Female Literacy* | *Distance to major roads* | *Weight* | *CR* |
| --- | --- | --- | --- | --- | --- | --- | --- |
| *Population Density* | 1.00 | 3.00 | 2.00 | 7.00 | 3.00 | 0.423 | 0.04 |
| *Distance to hospital* | 0.33 | 1.00 | 2.00 | 4.00 | 2.00 | 0.224 |  |
| *Elevation* | 0.50 | 0.50 | 1.00 | 3.00 | 2.00 | 0.174 |  |
| *Female Literacy* | 0.14 | 0.25 | 0.33 | 1.00 | 0.25 | 0.05 |  |
| *Distance to major roads* | 0.33 | 0.50 | 0.50 | 4.00 | 1.00 | 0.129 |  |

***Table S6: Seasonal Global Moran's I result***

| **Year and season** | **Pattern** | **Moran’s Index** | **Z score** | **P Value** | **Probability of random choices** |
| --- | --- | --- | --- | --- | --- |
| 2019-postmonsoon | Clustered | 0.29 | 3.22 | 0.001 | <1% |
| 2020-winter | Clustered | 0.19 | 2.21 | 0.027 | <5% |
| 2020-premonsoon | Clustered | 0.23 | 2.74 | 0.006 | <1% |
| 2020-monsoon | Clustered | 0.23 | 2.75 | 0.006 | <1% |
| 2020-postmonsoon | Clustered | 0.24 | 2.80 | 0.005 | <1% |
| 2021-winter | Clustered | 0.30 | 3.35 | 0.001 | <1% |
| 2021-premonsoon | Clustered | 0.24 | 2.82 | 0.004 | <1% |
| 2021-monsoon | Random | -0.02 | -.0.09 | 0.925 | Not significantly different |
| 2021-postmonsoon | Random | -0.03 | -0.59 | 0.555 | Not significantly different |
| 2022-winter | Random | -0.04 | -0.48 | 0.628 | Not significantly different |
| 2022-premonsoon | Random | -0.03 | -0.32 | 0.750 | Not significantly different |
| 2022-monsoon | Random | -0.03 | -0.31 | 0.759 | Not significantly different |
| 2022-postmonsoon | Random | 0.03 | 0.75 | 0.453 | Not significantly different |
| 2023-winter | Clustered | 0.18 | 2.42 | 0.015 | <5% |
| 2023-premonsoon | Random | -0.02 | -0.12 | 0.906 | Not significantly different |
| 2023-monsoon | Random | -0.05 | -0.40 | 0.690 | Not significantly different |
| 2023-postmonsoon | Random | -0.03 | -0.20 | 0.839 | Not significantly different |
| 2024 Winter | Clustered | 0.43 | 4.63 | 0.000004 | <1% |
| 2024-premonsoon | Random | 0.01 | 0.29 | 0.77 | Not significantly different |
| 2024-monsoon | Random | -0.05 | -0.55 | 0.58 | Not significantly different |
| 2024-postmonsoon | Random | -0.01 | 0.11 | 0.91 | Not significantly different |

***Table S7: Yearly Global Moran's I result***

| **Year** | **Pattern** | **Moran’s Index** | **Z score** | **P Value** | **Probability of random choices** |
| --- | --- | --- | --- | --- | --- |
| 2019 | Clustered | 0.29 | 3.21 | 0.001 | <1% |
| 2020 | Random | 0.04 | 1.05 | 0.294 | Not significantly different |
| 2021 | Clustered | 0.04 | 1.95 | 0.051 | <10% |
| 2022 | Clustered | 0.11 | 1.92 | 0.055 | <10% |
| 2023 | Clustered | 0.43 | 4.71 | 0.0002 | <1% |
| 2024 | Clustered | 0.22 | 2.55 | 0.010 | <5% |

***Table S8: Multicollinearity assessment of predictors included in the final negative binomial mixed effect model using generalized variance inflation factors (GVIF)***

| **Variable** | **Generalized Variance Inflation factor (GVIF)** | **Degree of freedom (df)** | **Adjusted GVIF** |
| --- | --- | --- | --- |
| Season | 4.808 | 3 | 1.299 |
| Time trend (t) | 1.702 | 1 | 1.304 |
| Previous-month lagged temperature (z) | 2.386 | 1 | 1.545 |
| Previous-month lagged precipitation (z) | 2.543 | 1 | 1.595 |
| COVID period (2020-2021) | 1.677 | 1 | 1.295 |
| NDBI (z) | 1.778 | 1 | 1.334 |
| Elevation (z) | 2.301 | 1 | 1.517 |
| Distance to water (z) | 1.81 | 1 | 1.345 |
| Distance to road (z) | 1.801 | 1 | 1.342 |
| Female literacy (z) | 1.705 | 1 | 1.306 |
| Population density (z) | 1.539 | 1 | 1.241 |

***Table S9: Jaccard Similarity Index for same-season consecutive-year hotspot comparisons (2019–2024). Jaccard = Shared hotspots / Either hotspot. (Winter: win, Pre-monsoon: PreM, Monsoon: Mon, Post-monsoon: PostM).***

| ***Season*** | ***Comparison*** | ***Shared Hotspots (Both = 1)*** | ***Either Hotspot (≥1 = 1)*** | ***Year 1 Only*** | ***Year 2 Only*** | ***Neither (Both = 0)*** | ***Total Districts*** | ***Jaccard Similarity Index*** |
| --- | --- | --- | --- | --- | --- | --- | --- | --- |
| ***Winter*** | Win_2020 vs Win_2021 | 20 | 28 | 8 | 0 | 36 | 64 | 0.71 |
| ***Winter*** | Win_2021 vs Win_2022 | 0 | 23 | 20 | 3 | 41 | 64 | 0.00 |
| ***Winter*** | Win_2022 vs Win_2023 | 3 | 4 | 0 | 1 | 60 | 64 | 0.75 |
| ***Winter*** | Win_2023 vs Win_2024 | 0 | 11 | 4 | 7 | 53 | 64 | 0.00 |
| ***Pre-monsoon*** | PreM_2020 vs PreM_2021 | 10 | 27 | 7 | 10 | 37 | 64 | 0.37 |
| ***Pre-monsoon*** | PreM_2021 vs PreM_2022 | 8 | 20 | 12 | 0 | 44 | 64 | 0.40 |
| ***Pre-monsoon*** | PreM_2022 vs PreM_2023 | 8 | 8 | 0 | 0 | 56 | 64 | 1.00 |
| ***Pre-monsoon*** | PreM_2023 vs PreM_2024 | 3 | 15 | 5 | 7 | 49 | 64 | 0.20 |
| ***Monsoon*** | Mon_2020 vs Mon_2021 | 1 | 24 | 16 | 7 | 40 | 64 | 0.04 |
| ***Monsoon*** | Mon_2021 vs Mon_2022 | 8 | 10 | 0 | 2 | 54 | 64 | 0.80 |
| ***Monsoon*** | Mon_2022 vs Mon_2023 | 4 | 16 | 6 | 6 | 48 | 64 | 0.25 |
| ***Monsoon*** | Mon_2023 vs Mon_2024 | 6 | 11 | 4 | 1 | 53 | 64 | 0.55 |
| ***Post-monsoon*** | PostM_2019 vs PostM_2020 | 25 | 30 | 3 | 2 | 34 | 64 | 0.83 |
| ***Post-monsoon*** | PostM_2020 vs PostM_2021 | 6 | 29 | 21 | 2 | 35 | 64 | 0.21 |
| ***Post-monsoon*** | PostM_2021 vs PostM_2022 | 0 | 12 | 8 | 4 | 52 | 64 | 0.00 |
| ***Post-monsoon*** | PostM_2022 vs PostM_2023 | 1 | 17 | 3 | 13 | 47 | 64 | 0.06 |
| ***Post-monsoon*** | PostM_2023 vs PostM_2024 | 2 | 19 | 12 | 5 | 45 | 64 | 0.11 |

***Table S10: Regional distribution of dengue hotspot districts by season and year (2019–2024). Values show number of hotspot districts and percentage of total hotspot districts for that season-year. Bold values indicate dominant region (≥50%).***

| ***Season*** | ***Year*** | ***Total Hotspot Districts*** | ***Coastal/South*** | ***South-central Delta*** | ***Central/Peri-Dhaka*** | ***Western*** | ***Eastern*** |
| --- | --- | --- | --- | --- | --- | --- | --- |
| ***Post-monsoon*** | 2019 | 28 | 11 (39.3%) | 7 (25.0%) | 3 (10.7%) | 6 (21.4%) | 1 (3.6%) |
|  | 2020 | 27 | 9 (33.3%) | 7 (25.9%) | 3 (11.1%) | 8 (29.6%) | 0 (0.0%) |
|  | 2021 | 8 | 0 (0.0%) | 3 (37.5%) | **5 (62.5%)** | 0 (0.0%) | 0 (0.0%) |
|  | 2022 | 4 | **3 (75.0%)** | 1 (25.0%) | 0 (0.0%) | 0 (0.0%) | 0 (0.0%) |
|  | 2023 | 14 | **13 (92.9%)** | 0 (0.0%) | 0 (0.0%) | 0 (0.0%) | 1 (7.1%) |
|  | 2024 | 7 | 1 (14.3%) | **4 (57.1%)** | 1 (14.3%) | 0 (0.0%) | 1 (14.3%) |
| ***Winter*** | 2020 | 28 | 10 (35.7%) | 7 (25.0%) | 3 (10.7%) | 8 (28.6%) | 0 (0.0%) |
|  | 2021 | 20 | 6 (30.0%) | 7 (35.0%) | 3 (15.0%) | 4 (20.0%) | 0 (0.0%) |
|  | 2022 | 3 | **3 (100.0%)** | 0 (0.0%) | 0 (0.0%) | 0 (0.0%) | 0 (0.0%) |
|  | 2023 | 4 | **4 (100.0%)** | 0 (0.0%) | 0 (0.0%) | 0 (0.0%) | 0 (0.0%) |
|  | 2024 | 7 | 1 (14.3%) | **4 (57.1%)** | 1 (14.3%) | 0 (0.0%) | 1 (14.3%) |
| ***Pre-monsoon*** | 2020 | 17 | 4 (23.5%) | 4 (23.5%) | 1 (5.9%) | 8 (47.1%) | 0 (0.0%) |
|  | 2021 | 20 | 1 (5.0%) | 7 (35.0%) | 5 (25.0%) | 4 (20.0%) | 3 (15.0%) |
|  | 2022 | 8 | 0 (0.0%) | 3 (37.5%) | **5 (62.5%)** | 0 (0.0%) | 0 (0.0%) |
|  | 2023 | 8 | 0 (0.0%) | 3 (37.5%) | **5 (62.5%)** | 0 (0.0%) | 0 (0.0%) |
|  | 2024 | 10 | 1 (10.0%) | **6 (60.0%)** | 0 (0.0%) | 2 (20.0%) | 1 (10.0%) |
| ***Monsoon*** | 2020 | 17 | 5 (29.4%) | 4 (23.5%) | 0 (0.0%) | 8 (47.1%) | 0 (0.0%) |
|  | 2021 | 8 | 0 (0.0%) | 3 (37.5%) | **5 (62.5%)** | 0 (0.0%) | 0 (0.0%) |
|  | 2022 | 10 | 2 (20.0%) | 3 (30.0%) | **5 (50.0%)** | 0 (0.0%) | 0 (0.0%) |
|  | 2023 | 10 | 2 (20.0%) | **5 (50.0%)** | 2 (20.0%) | 0 (0.0%) | 1 (10.0%) |
|  | 2024 | 7 | 1 (14.3%) | **4 (57.1%)** | 1 (14.3%) | 0 (0.0%) | 1 (14.3%) |

***Table S11: Model comparison (based on AIC)***

| **Model** | **df** | **AIC** |
| --- | --- | --- |
| Poisson | 15 | 780436.4 |
| Negative binomial | 16 | 21549.28 |
| Zero-inflated negative binomial | 17 | 21551.28 |

***Table S12: Incidence Rate Ratio (IRR) table for negative binomial mixed effect regression model (winter is the reference season, continuous predictors are standardized)***

| **Variable** | **IRR** | **95% CI lower** | **95% CI upper** | **p-value** |
| --- | --- | --- | --- | --- |
| Season: pre-monsoon vs winter | 0.004 | 0.003 | 0.006 | <0.001 |
| Season: monsoon vs winter | 0.527 | 0.347 | 0.798 | 0.003 |
| Season: post-monsoon vs winter | 1.319 | 0.914 | 1.903 | 0.139 |
| Time trend | 1.001 | 0.995 | 1.006 | 0.767 |
| Previous-month temperature (z) | 4.168 | 3.485 | 4.985 | <0.001 |
| Previous-month precipitation (z) | 1.621 | 1.408 | 1.866 | <0.001 |
| COVID period (2020-2021) | 0.030 | 0.024 | 0.037 | <0.001 |
| NDBI (z) | 0.620 | 0.428 | 0.899 | 0.012 |
| Elevation (z) | 1.271 | 0.834 | 1.938 | 0.264 |
| Distance to water (z) | 0.838 | 0.576 | 1.219 | 0.355 |
| Distance to road (z) | 0.814 | 0.561 | 1.182 | 0.279 |
| Female literacy (z) | 1.310 | 0.911 | 1.882 | 0.145 |
| Population density (z) | 1.773 | 1.256 | 2.502 | 0.001 |

**References**

Anselin, L. (1995). Local indicators of spatial association—LISA. *Geographical analysis*, *27*(2), 93-115.

ArcGIS pro. (n.d.). How Cluster and Outlier Analysis (Anselin Local Moran’s I) works— ArcGIS Pro | Documentation. Retrieved March 14, 2024, from pro.arcgis.com website: <https://pro.arcgis.com/en/pro-app/3.1/tool-reference/spatial-statistics/hhow-cluster-and-outlier-analysis-anselin-local-m.htm>

Mitchell, A. (2005). *The ESRI guide to GIS analysis* (Vol. 2). ESRI Press.

Ord, J. K., & Getis, A. (1995). Local spatial autocorrelation statistics: distributional issues and an application. *Geographical analysis*, *27*(4), 286-306.

Pallathadka, A., Chang, H., & Han, D. (2023). What explains spatial variations of COVID-19 vaccine hesitancy?: A social-ecological-technological systems approach. *Environmental Research: Health*, *1*(1), 011001.

Saaty, T. L. (1990). How to make a decision: the analytic hierarchy process. *European journal of operational research*, *48*(1), 9-26.

Ajim Ali, S., & Ahmad, A. (2018). Using analytic hierarchy process with GIS for Dengue risk mapping in Kolkata Municipal Corporation, West Bengal, India. *Spatial Information Research*, *26*(4), 449-469.
